# Supplementary material for: Use of sediment dwelling bivalves to biomonitor plastic particle pollution in intertidal regions; A review and study
Source: PLoS One. 2020 May 22;15(5):e0232879. doi: 10.1371/journal.pone.0232879 (PMC7244099; doi:10.1371/journal.pone.0232879)

Polyethylene

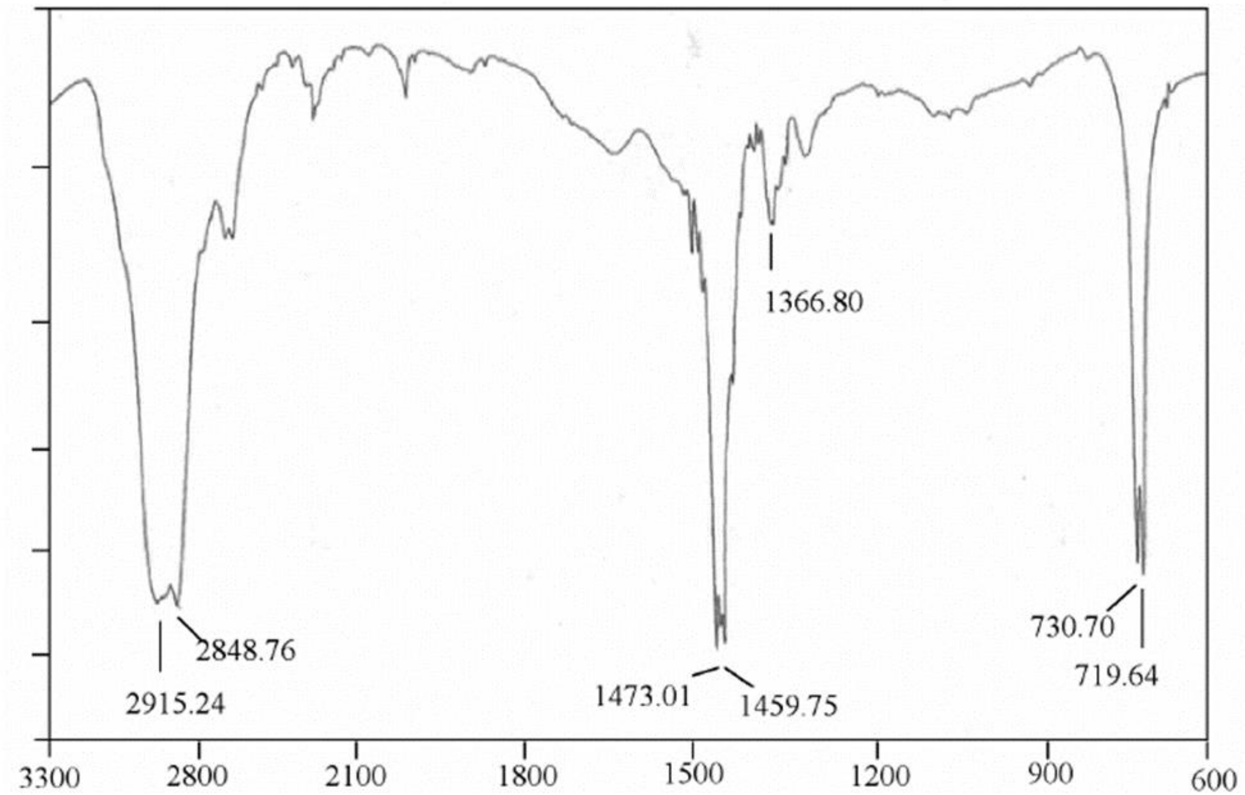

Polypropylene

HIT-NO=1992	SCORE= ( )	SDBS-NO=2720	IR-NIDA-00755 : LIQUID FILM
POLYPROPYLENE GLYCOL			
(C_3H_8O)			

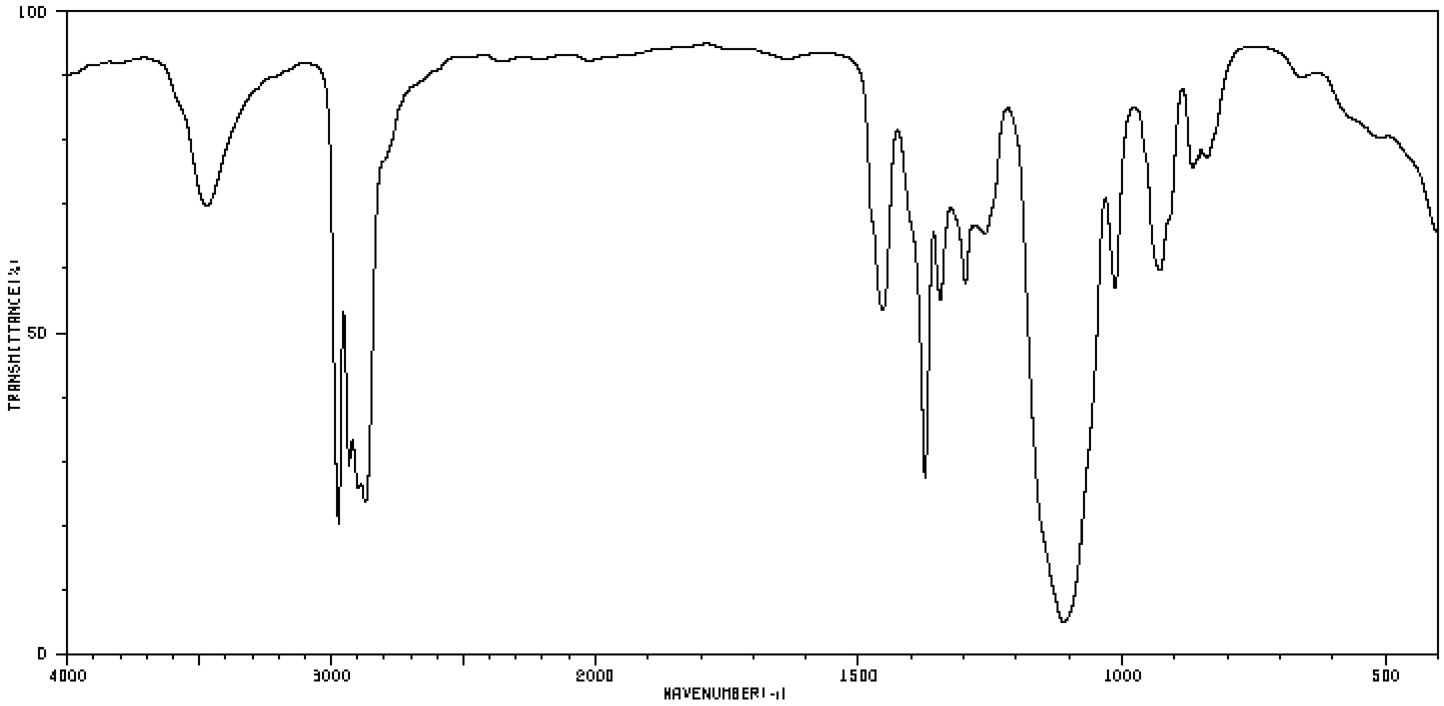

3472	66	1374	26	867	72
2972	19	1345	53	854	74
2932	27	1297	55	839	74
2897	24	1260	62		
2887	25	1109	4		
2870	22	1014	55		
1464	62	929	57		

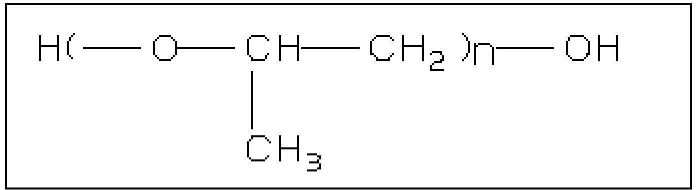

Polypropylene composite

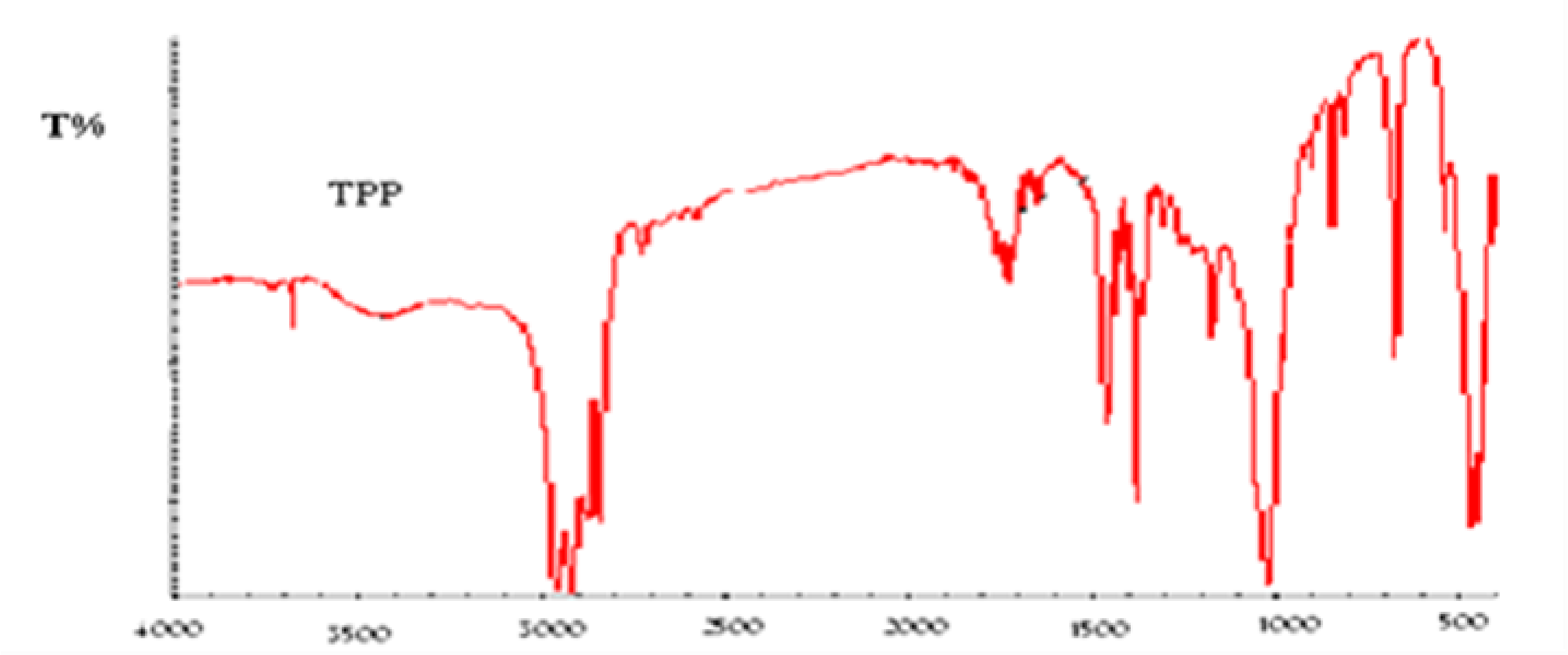

Nylon

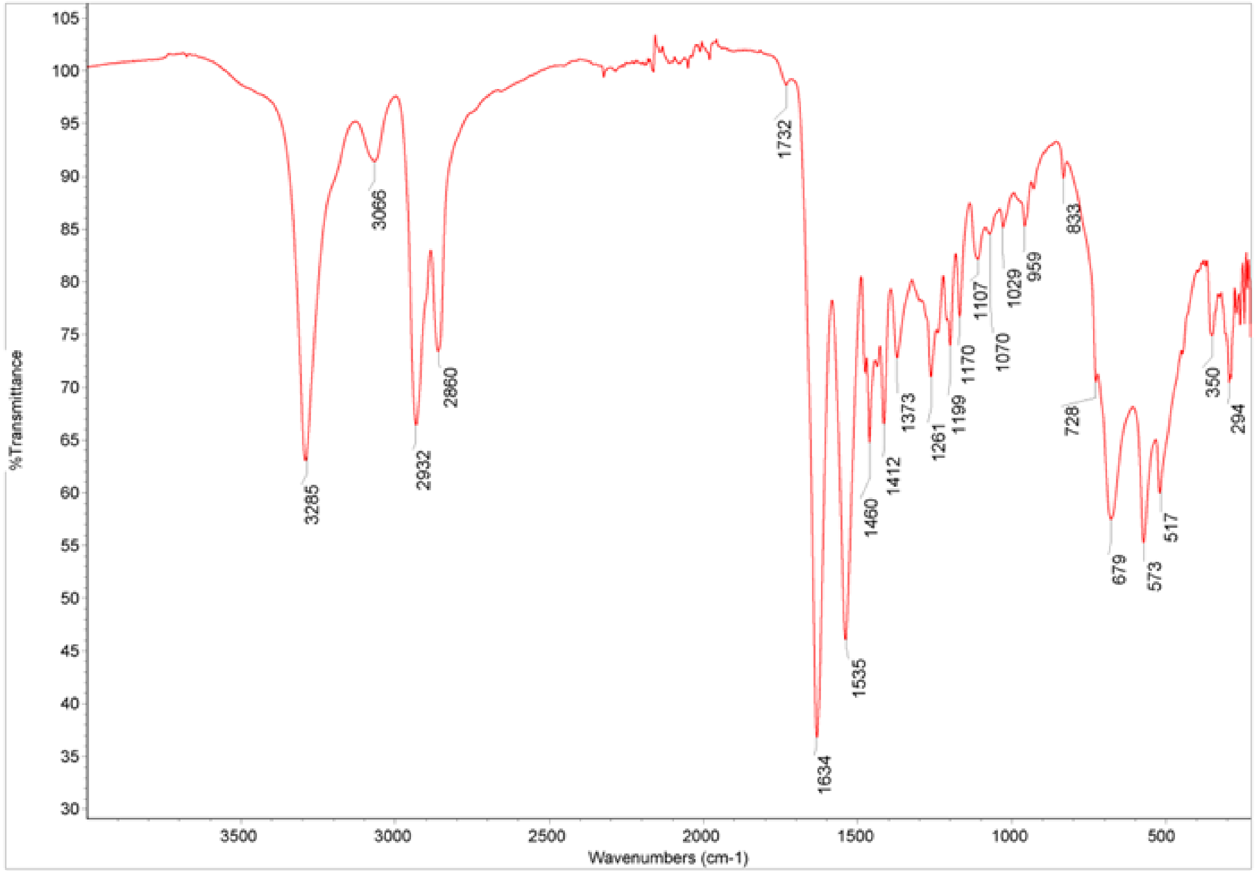

%Transmittance
105
100
95
90
85
80
75
70
65
60
55
50
45
40
35
30
3500
3000
2500
2000
1500
1000
500
Wavenumbers (cm-1)
3285
3066
2932
2860
1732
1634
1535
1460
1412
1373
1261
1199
1170
1107
1070
1029
959
833
728
679
573
517
350
294

Polystyrene

HIT-NO=1720	SCORE= ( )	SDBS-NO=1957	IR-NIDA-05943 : KBR DISC

POLYSTYRENE

(C_8H_8)

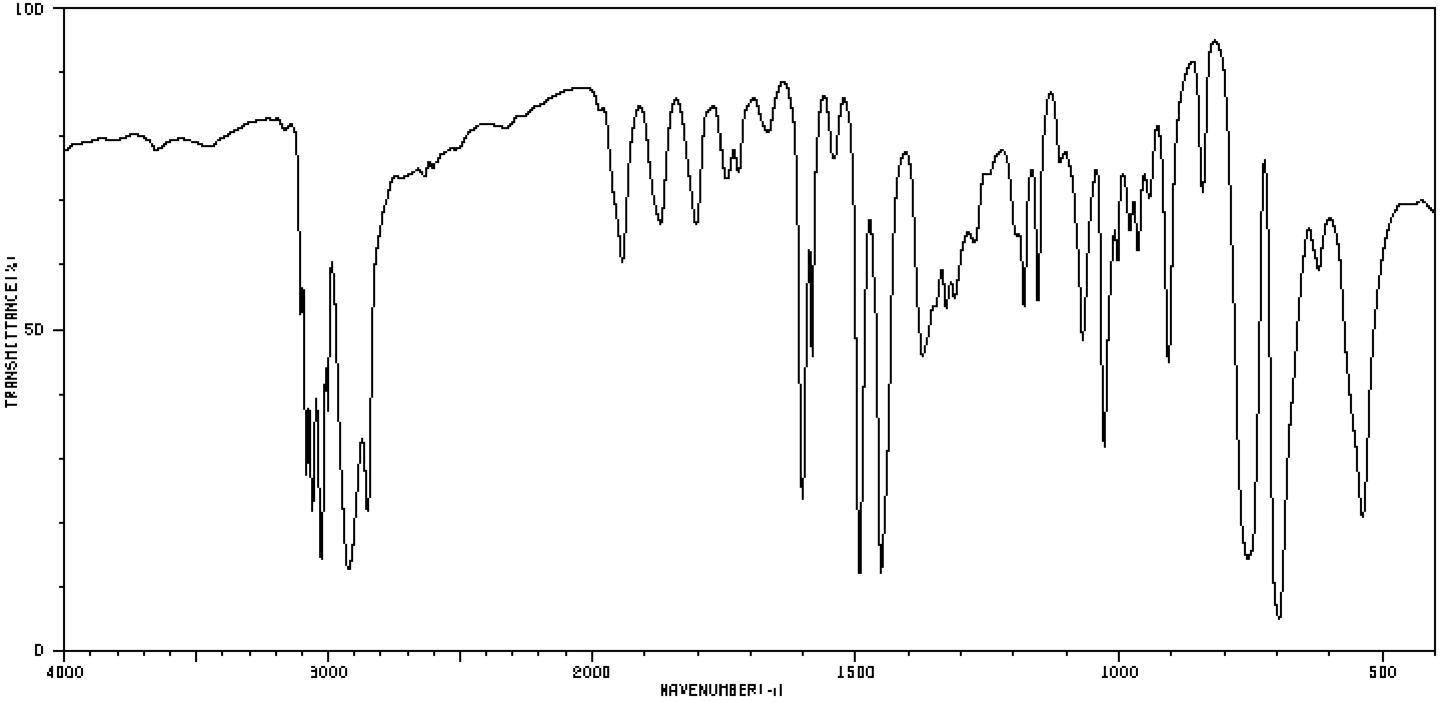

3103	60	2634	70	1493	11	1181	62	943	68
3083	26	1942	58	1453	11	1155	52	906	43
3060	21	1870	64	1374	43	1069	46	841	68
3026	13	1802	64	1329	62	1028	30	756	13
3002	36	1745	70	1313	52	1003	58	697	4
2924	12	1601	29	1275	80	980	62	622	57
2850	21	1583	43	1196	62	964	60	538	20

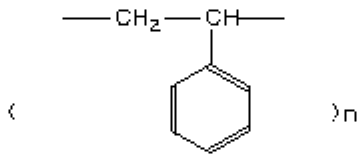

Polyurethane

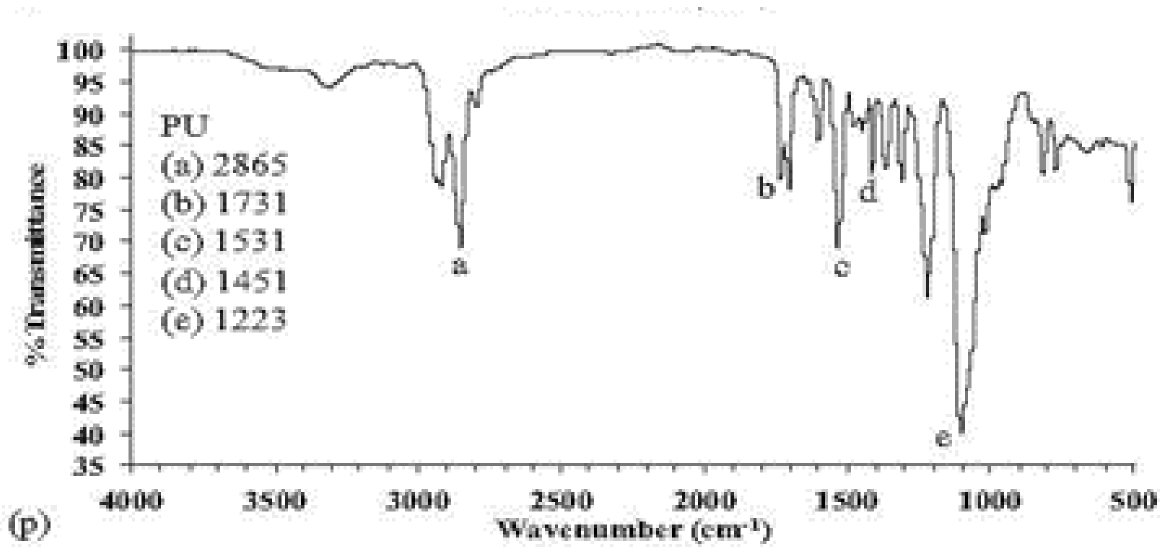

Poly(methyl methacrylate)

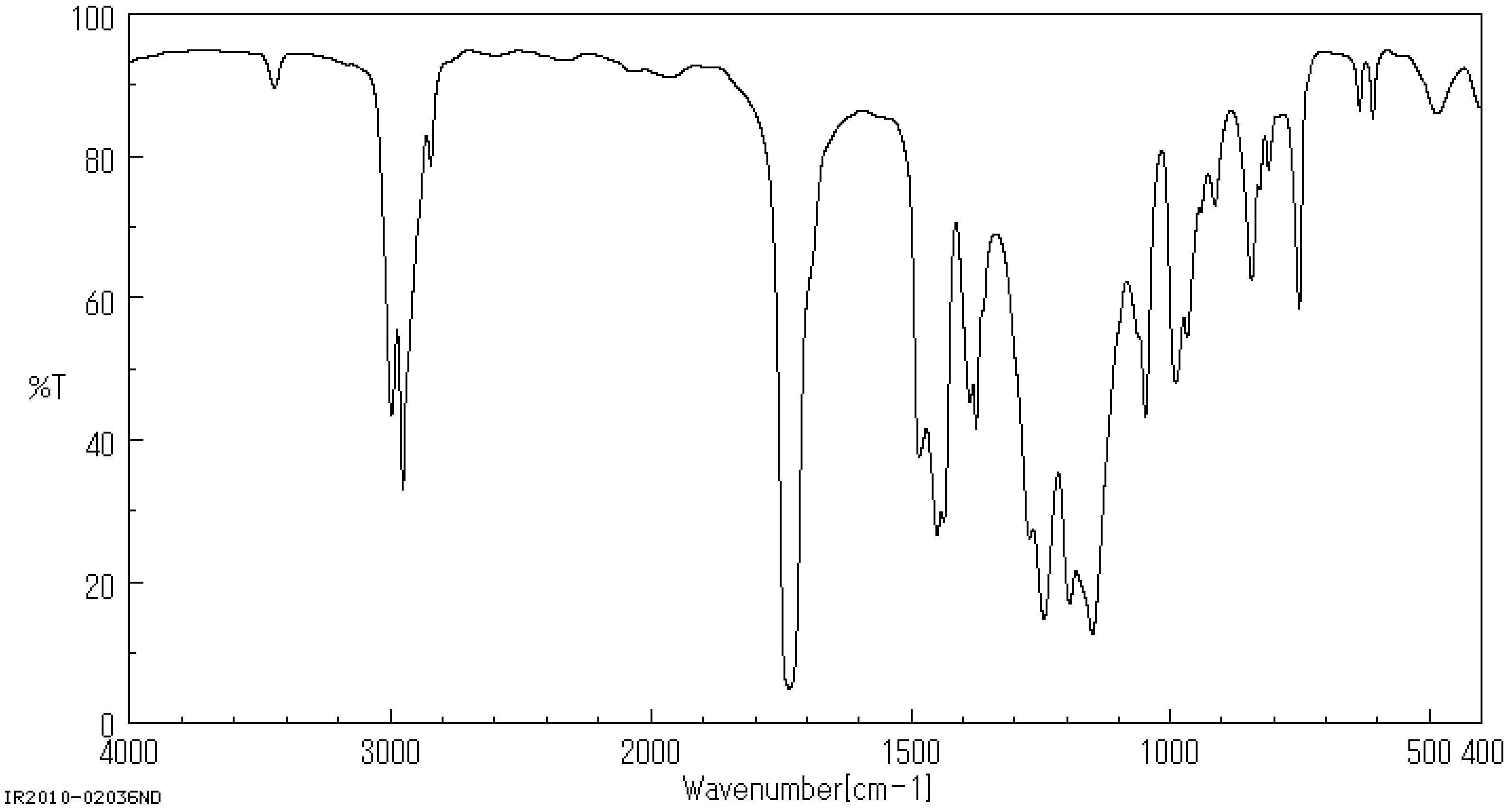

Cyanox

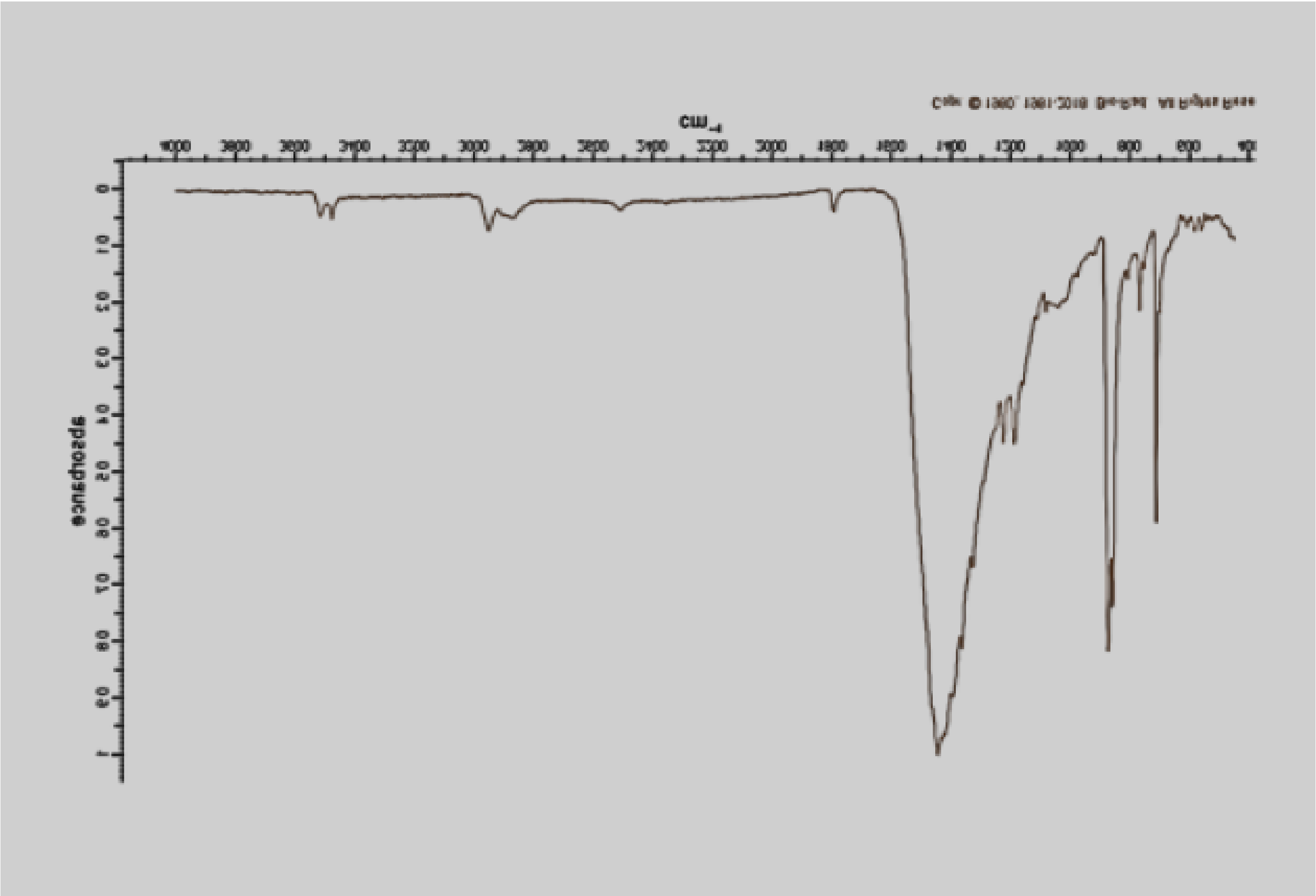

Bio-Rad Laboratories, Inc. SpectraBase; SpectraBase Compound ID=DaF9ALNWyoD
SpectraBase Spectrum ID=D4wMn8dQ8OL
http://spectrabase.com/spectrum/D4wMn8dQ8OL?a=SPECTRUM_D4wMn8dQ8OL (accessed Dec 01, 2019).

Sand (Si02)

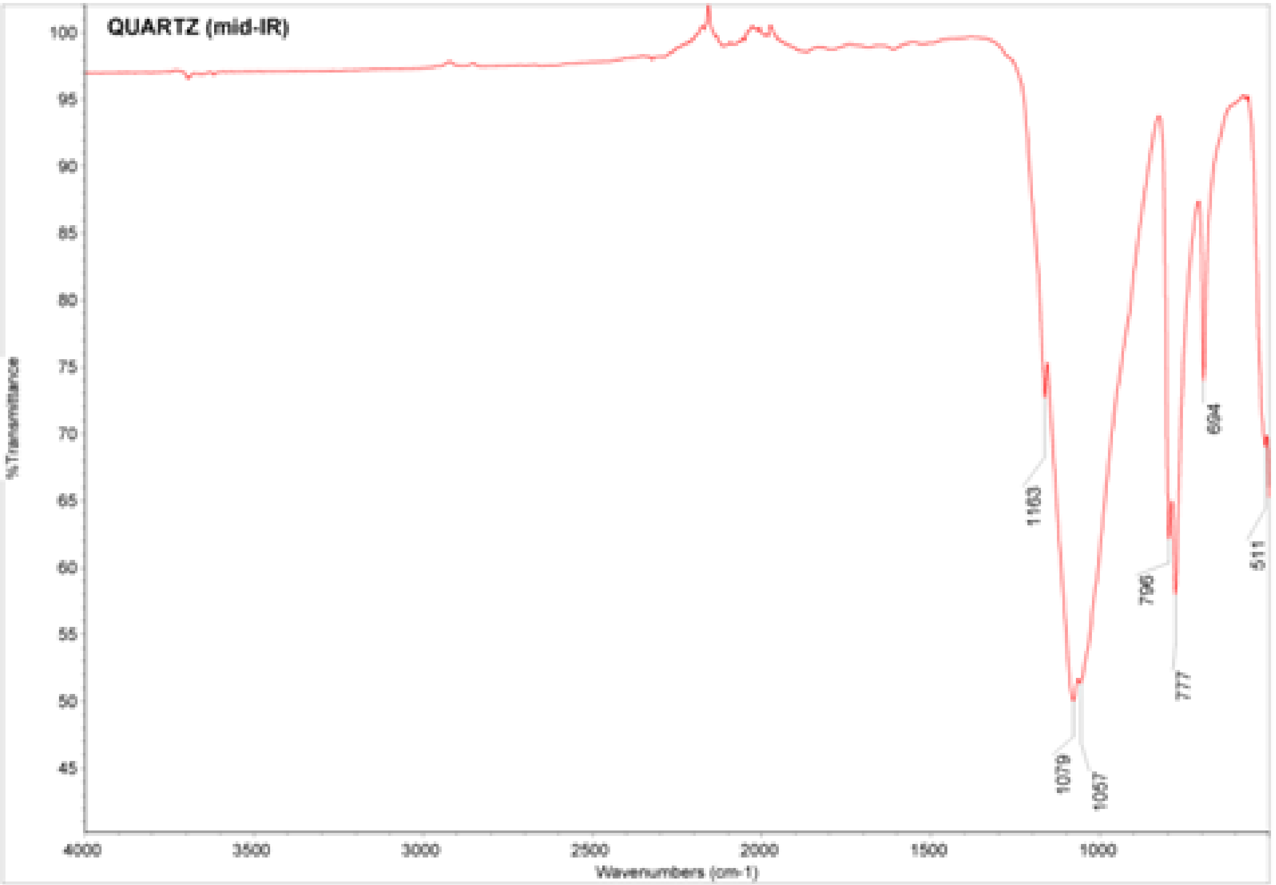

QUARTZ (mid-IR)
%Transmittance
100
95
90
85
80
75
70
65
60
55
50
45
4000
3500
3000
2500
2000
1500
1000
Wavenumbers (cm-1)
1163
1079
1057
796
777
694
511

Calcium carbonate

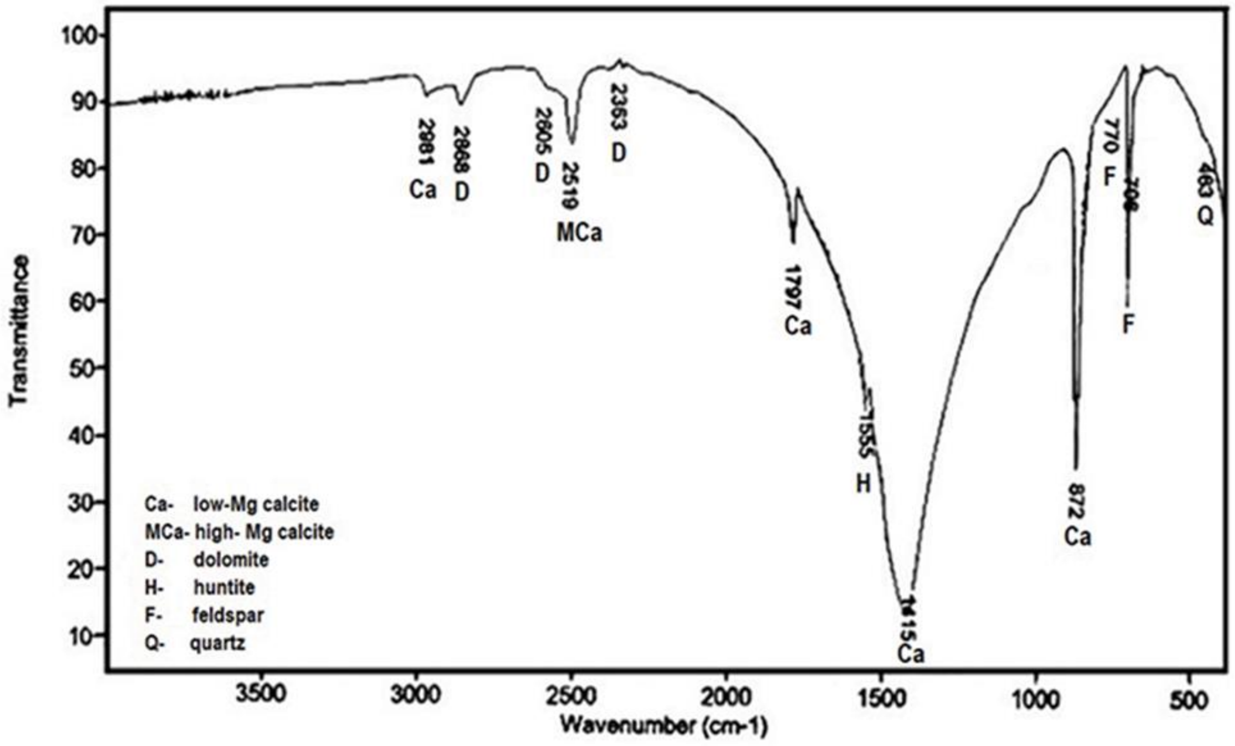

Supplement: S1 Fig — Supplementary material. (PDF) [file pone.0232879.s001.pdf]
