# Supplementary material for: Use of sediment dwelling bivalves to biomonitor plastic particle pollution in intertidal regions; A review and study
Source: PLoS One. 2020 May 22;15(5):e0232879. doi: 10.1371/journal.pone.0232879 (PMC7244099; doi:10.1371/journal.pone.0232879)

BMPC1bbis-1,2,3,4

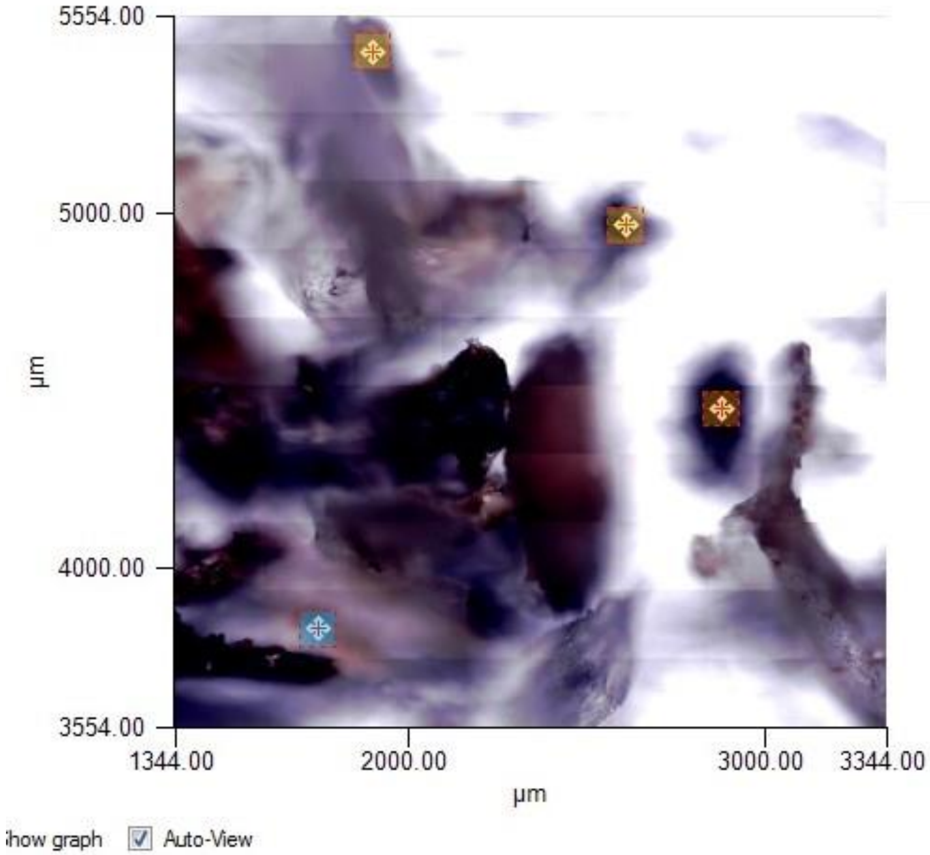

BMPCG1-1,2

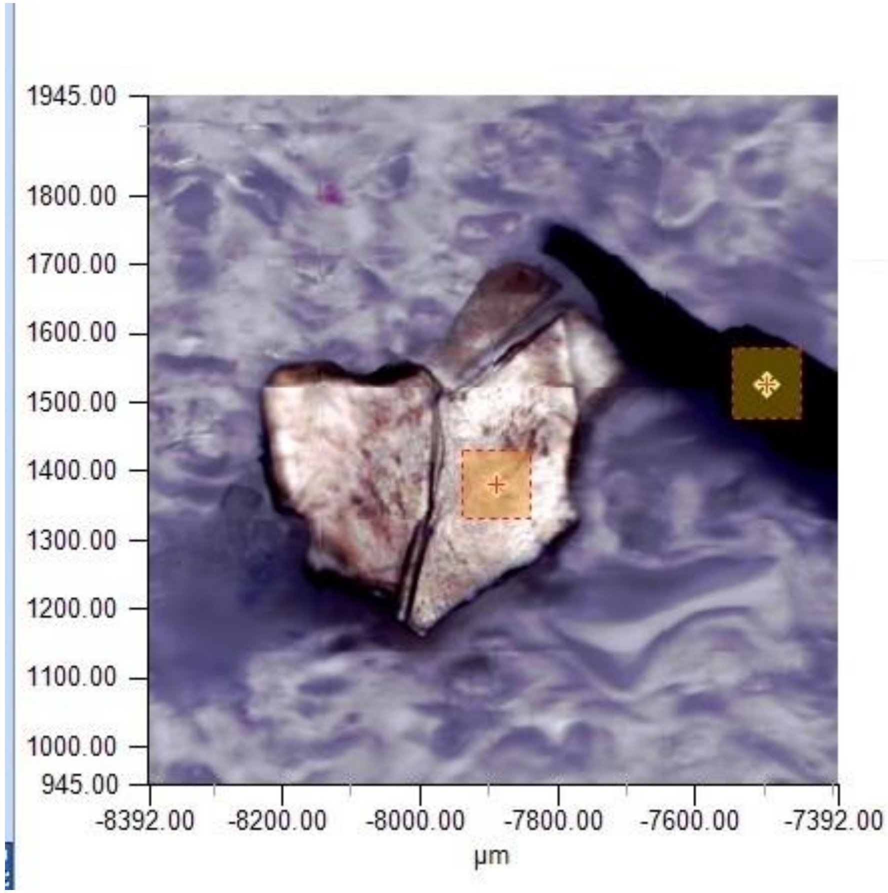

1945.00
1800.00
1700.00
1600.00
1500.00
1400.00
1300.00
1200.00
1100.00
1000.00
945.00
-8392.00
-8200.00
-8000.00
-7800.00
-7600.00
-7392.00
µm

BMPC1-1,2,3,4,5

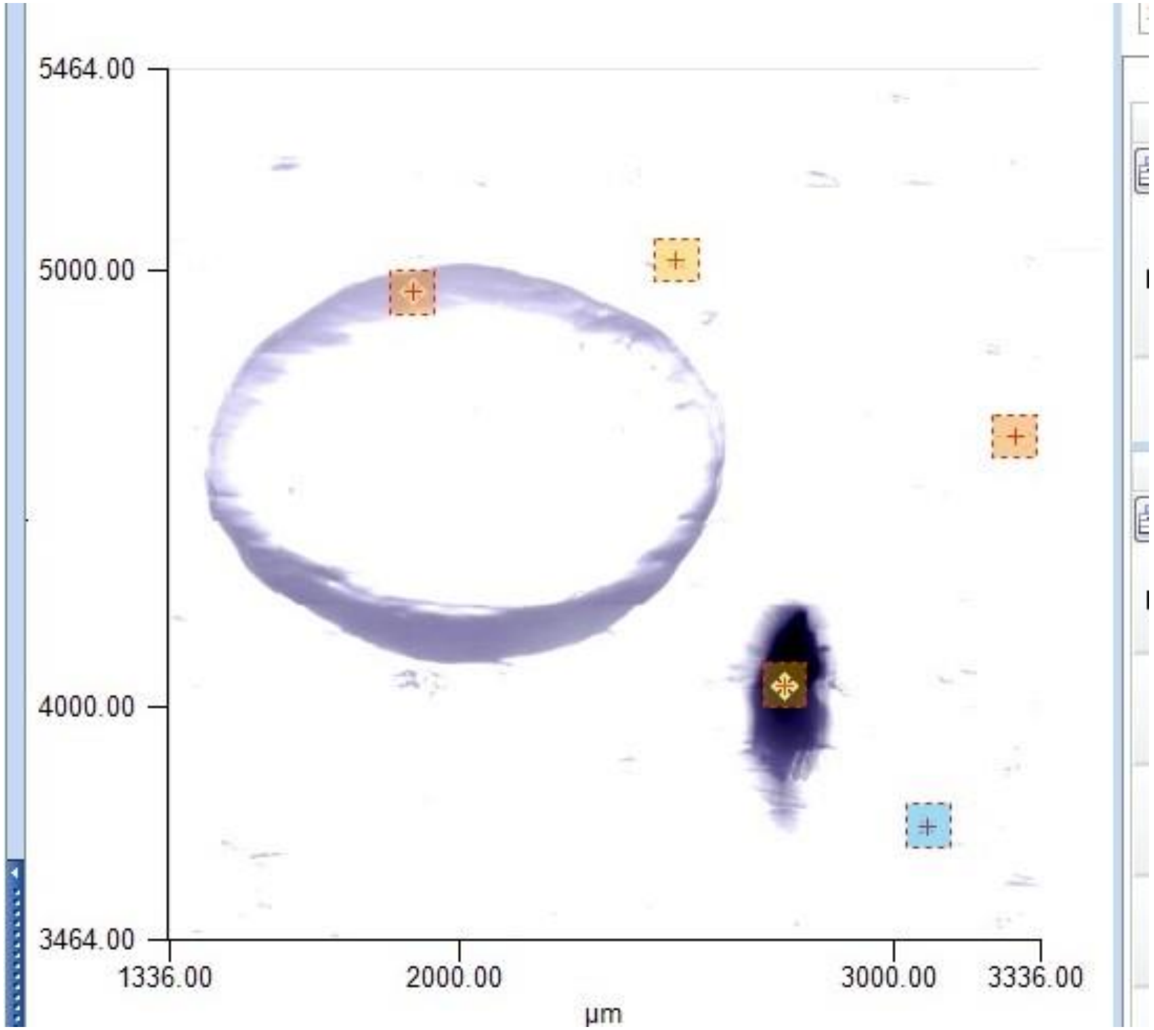
5464.00
5000.00
4000.00
3464.00
1336.00
2000.00
3000.00
3336.00
µm

BMPC1BBisSEC-1,2

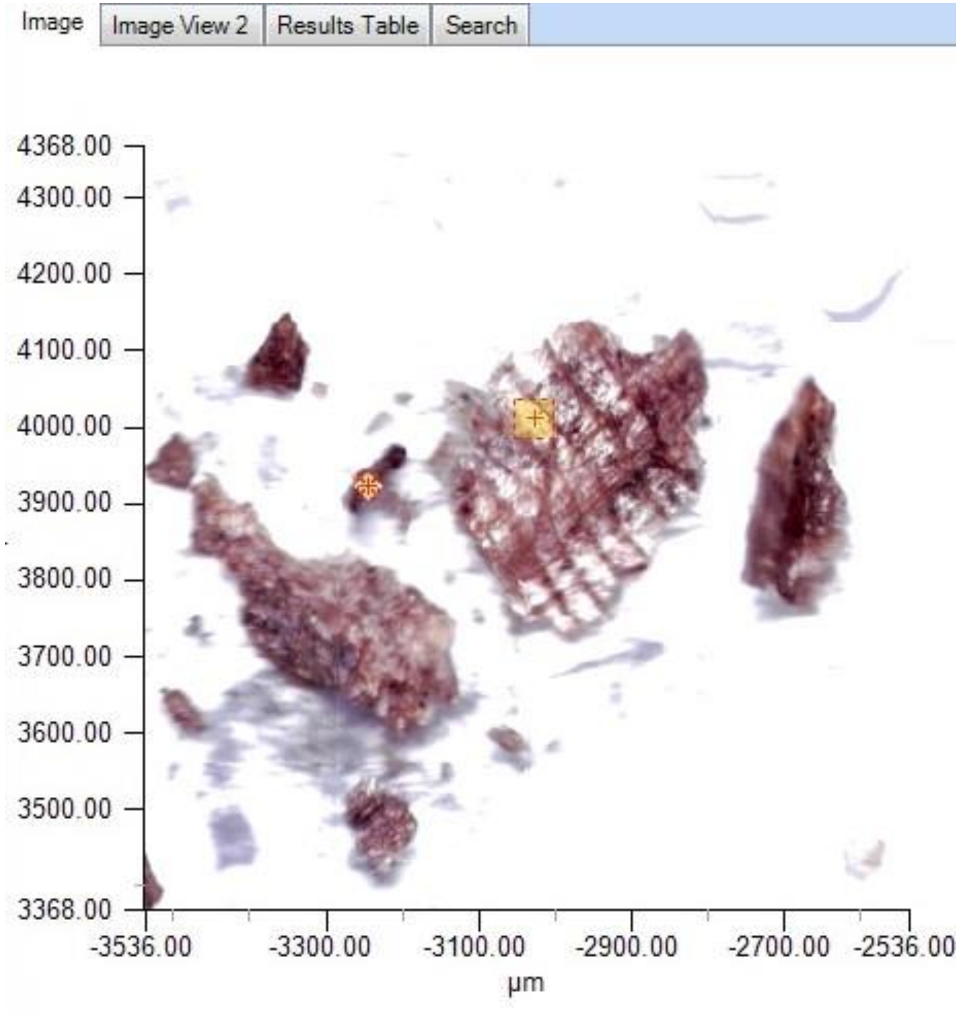

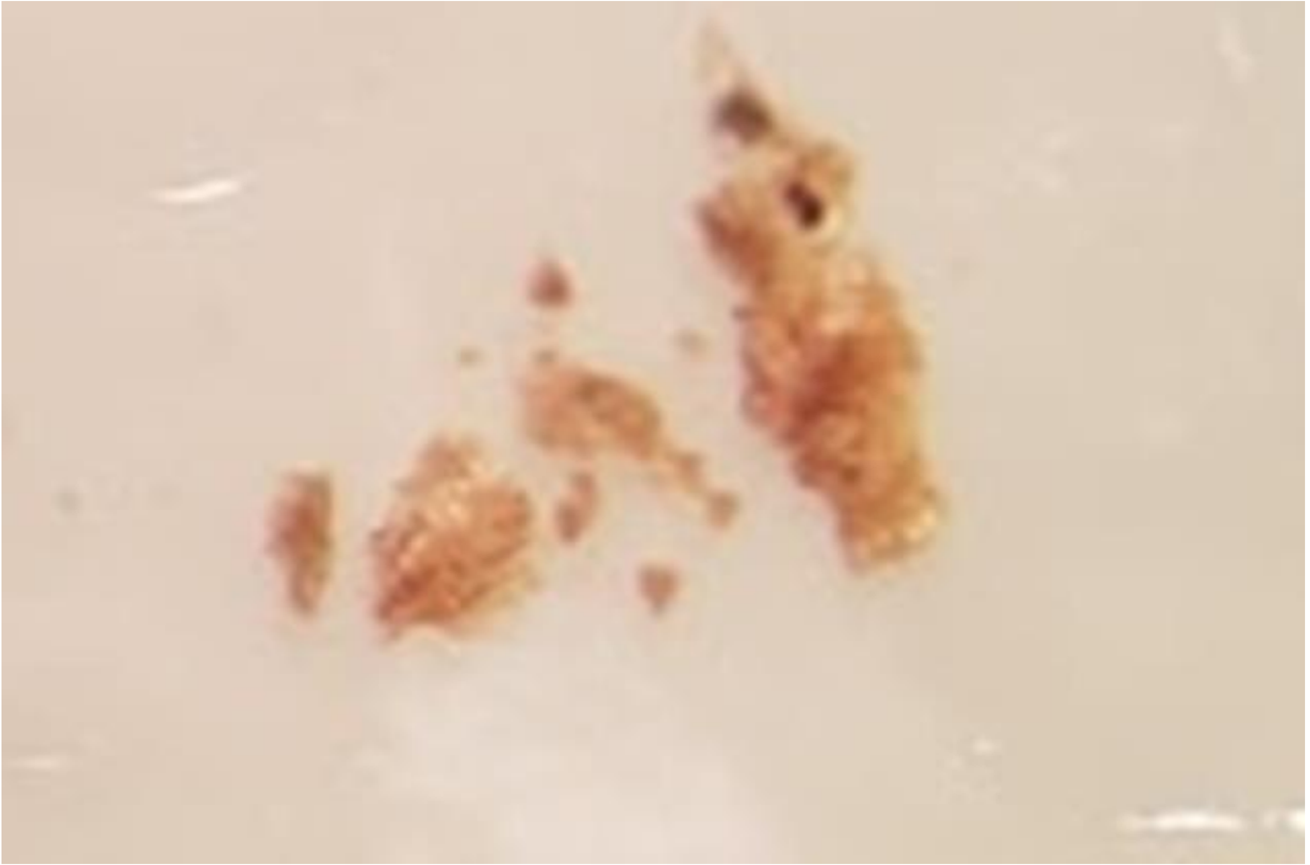

BMPC15; 1,2

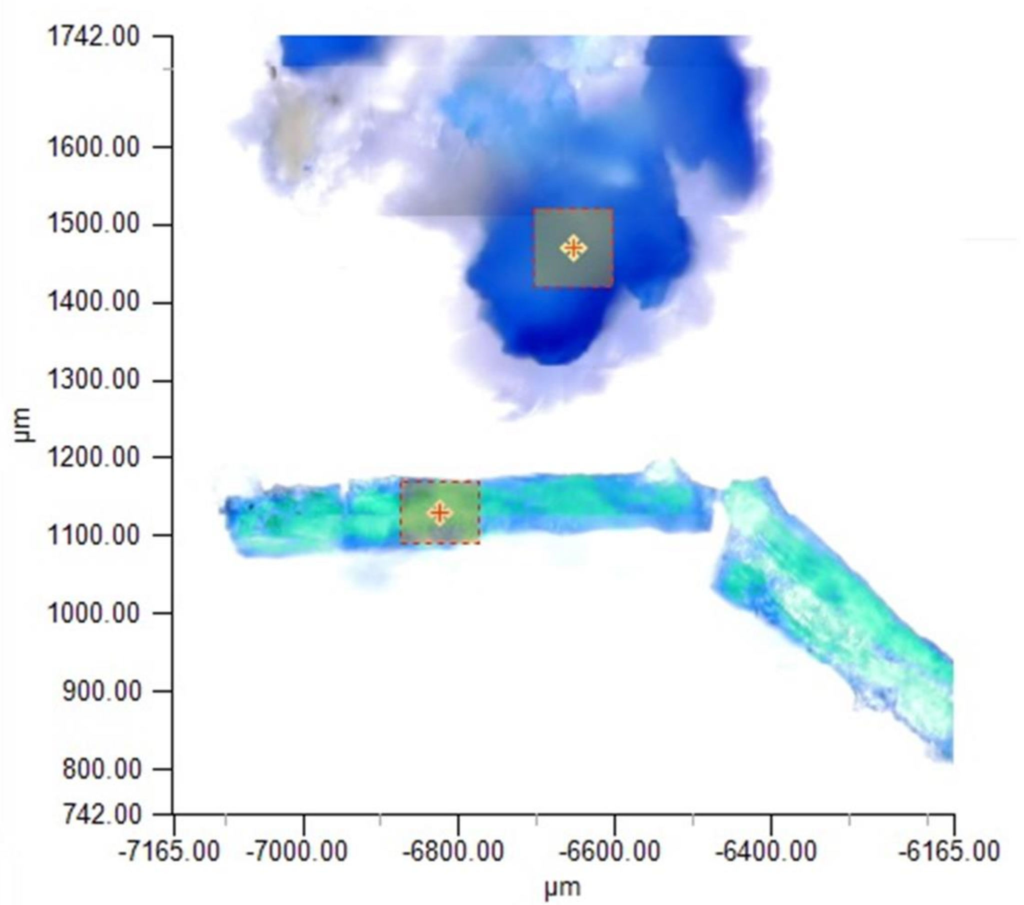

1742.00
1600.00
1500.00
1400.00
1300.00
1200.00
1100.00
1000.00
900.00
800.00
742.00
µm
-7165.00
-7000.00
-6800.00
-6600.00
-6400.00
-6165.00
µm

BMPCG2-1,2

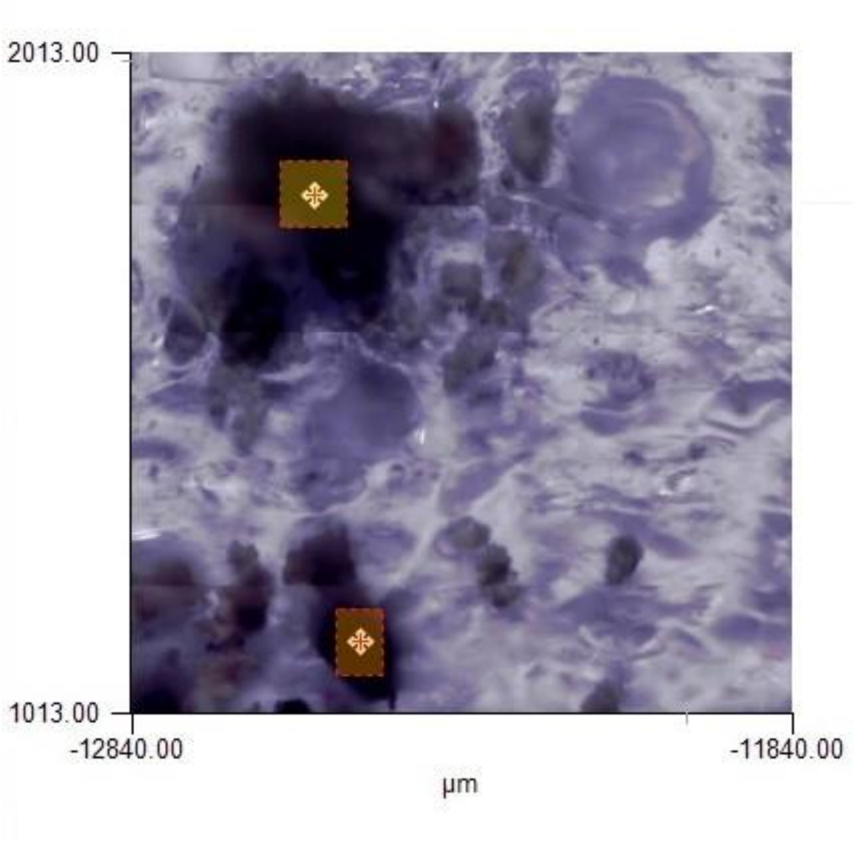

BMPCG3-1,2,3

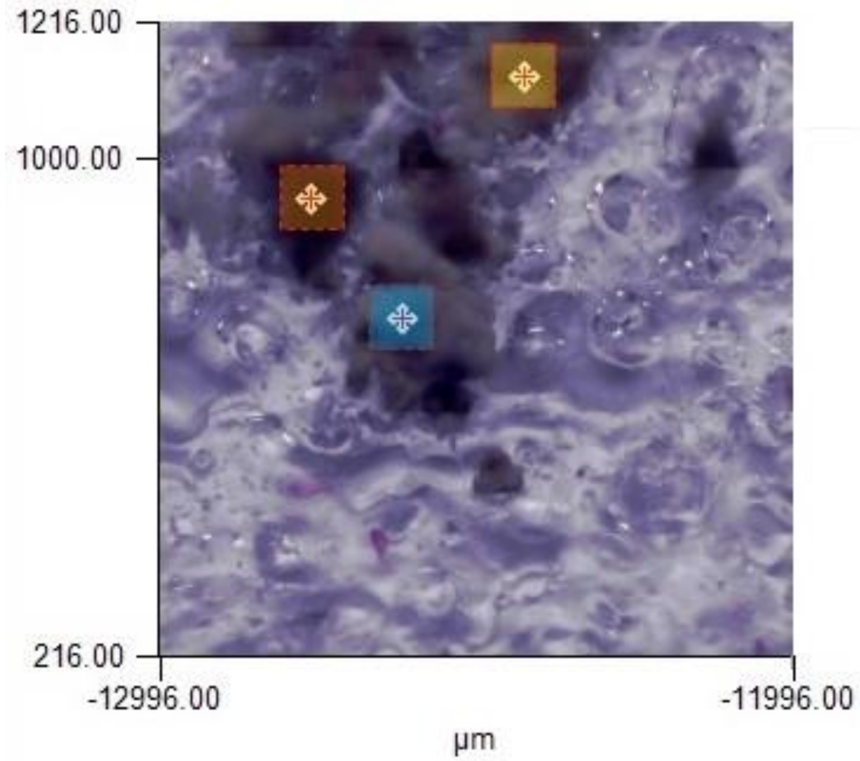

BMPC4

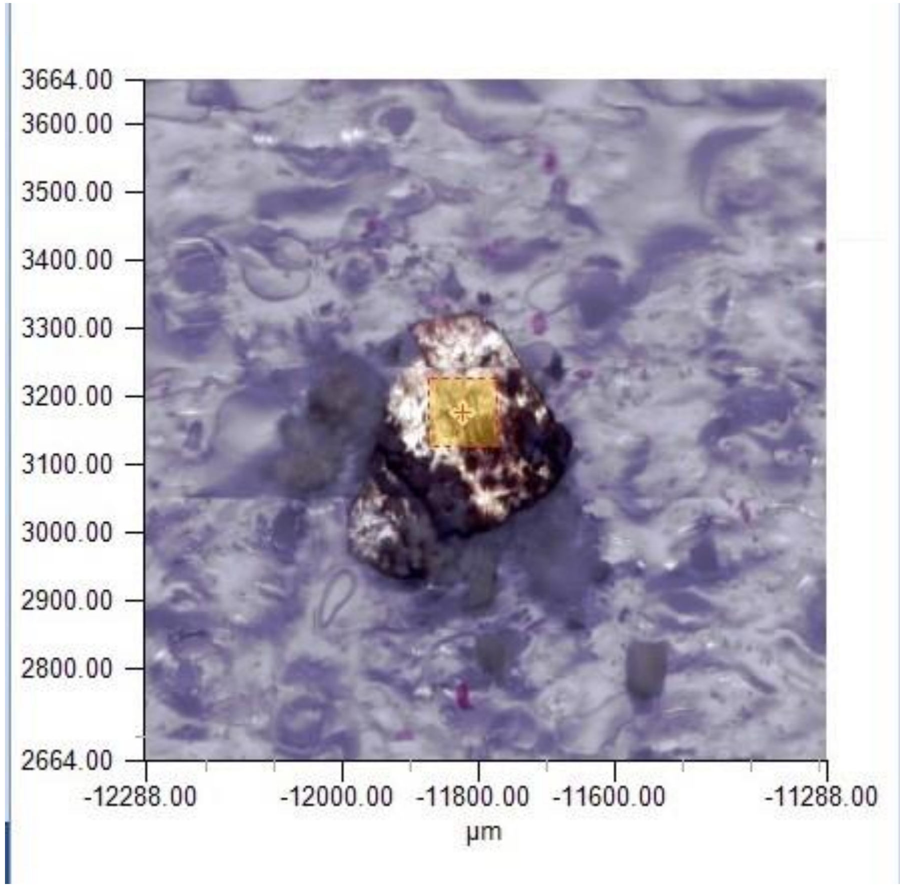

3664.00
3600.00
3500.00
3400.00
3300.00
3200.00
3100.00
3000.00
2900.00
2800.00
2664.00
-12288.00
-12000.00
-11800.00
-11600.00
-11288.00
µm

BMPCG5-1,2

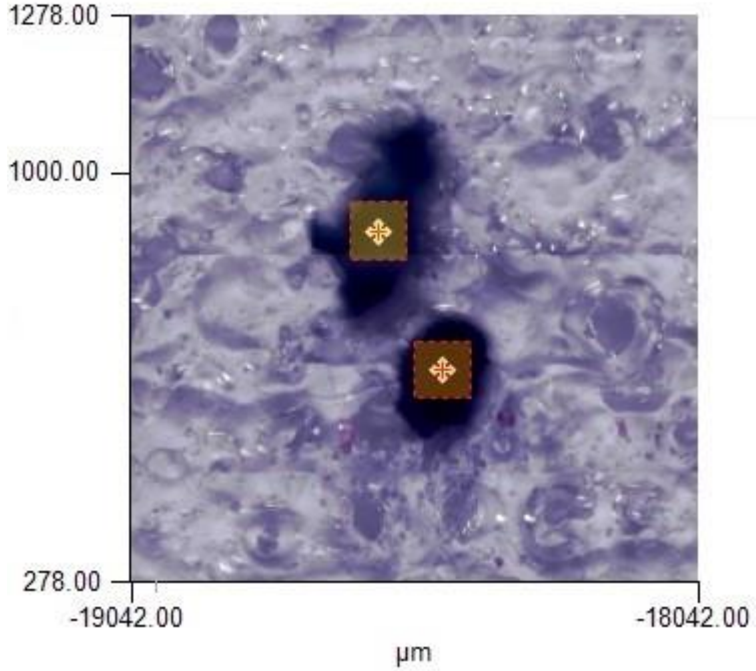

BMPVC7-1,2

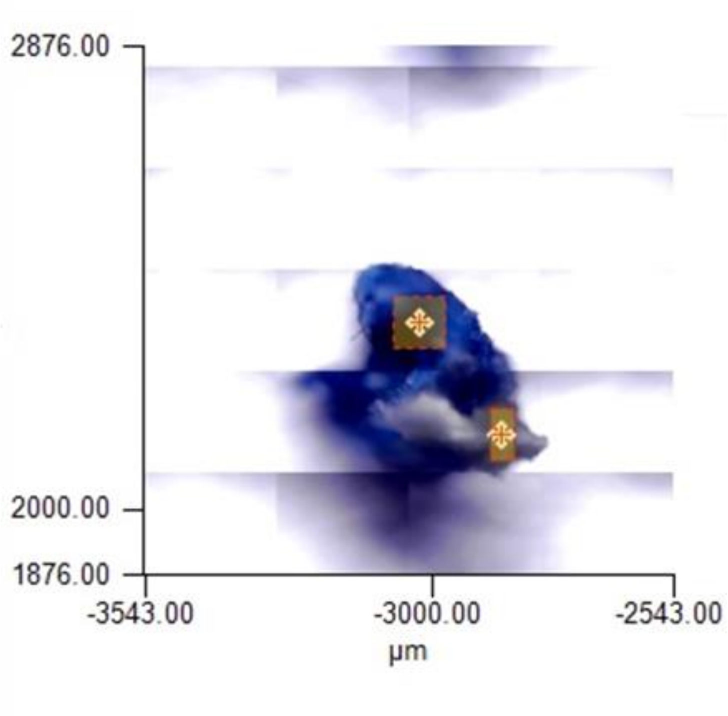

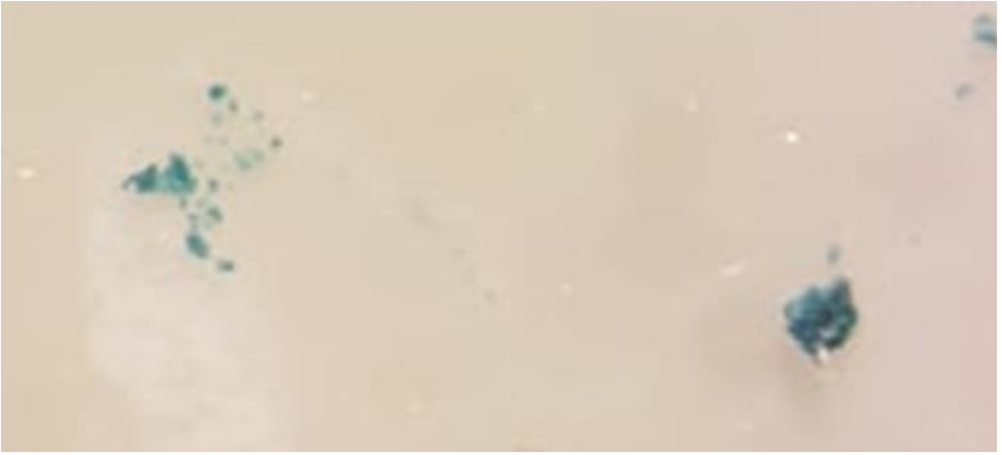

BMPVC8-1,2

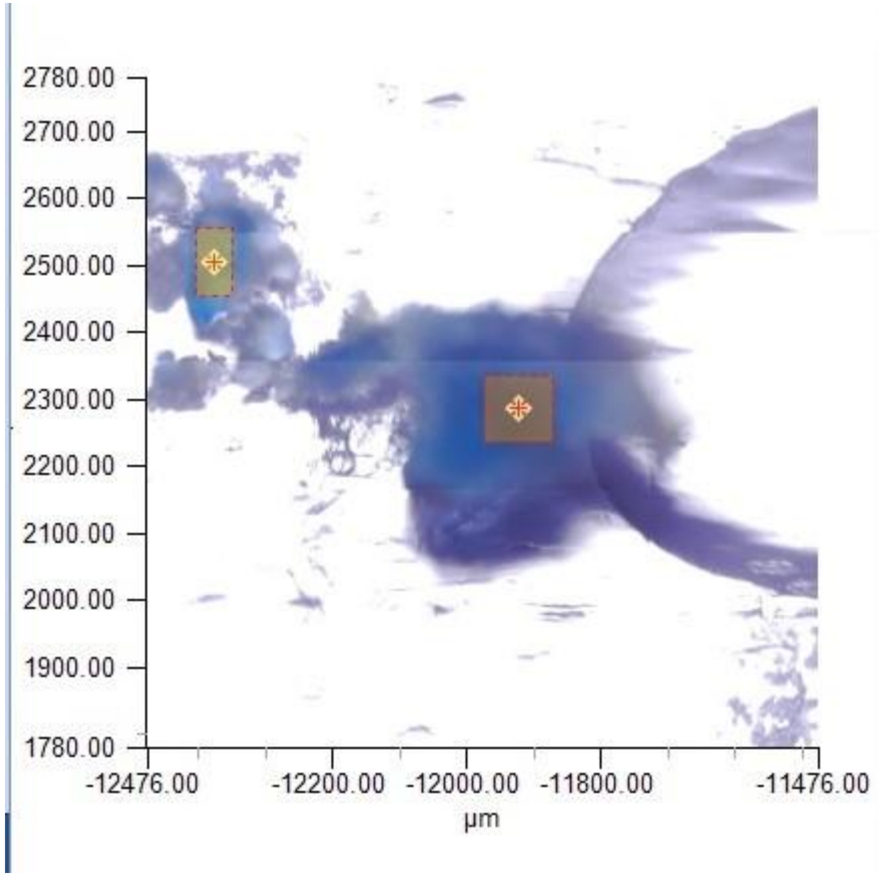

2780.00
2700.00
2600.00
2500.00
2400.00
2300.00
2200.00
2100.00
2000.00
1900.00
1780.00
-12476.00
-12200.00
-12000.00
-11800.00
-11476.00
µm

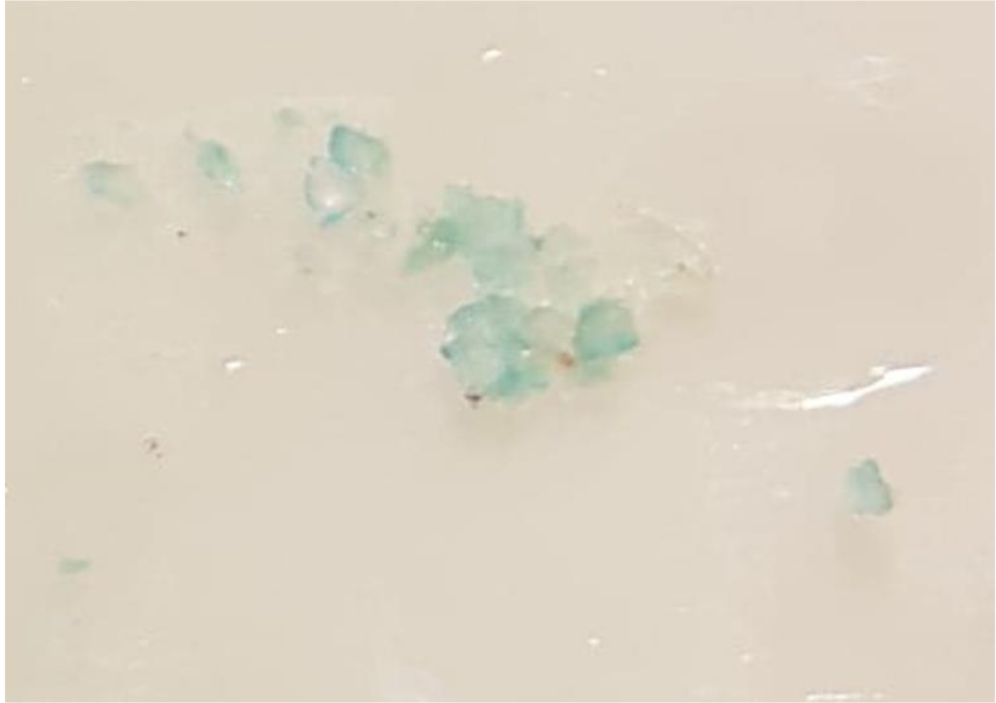

BMPALL 1,2,3,4,5,6

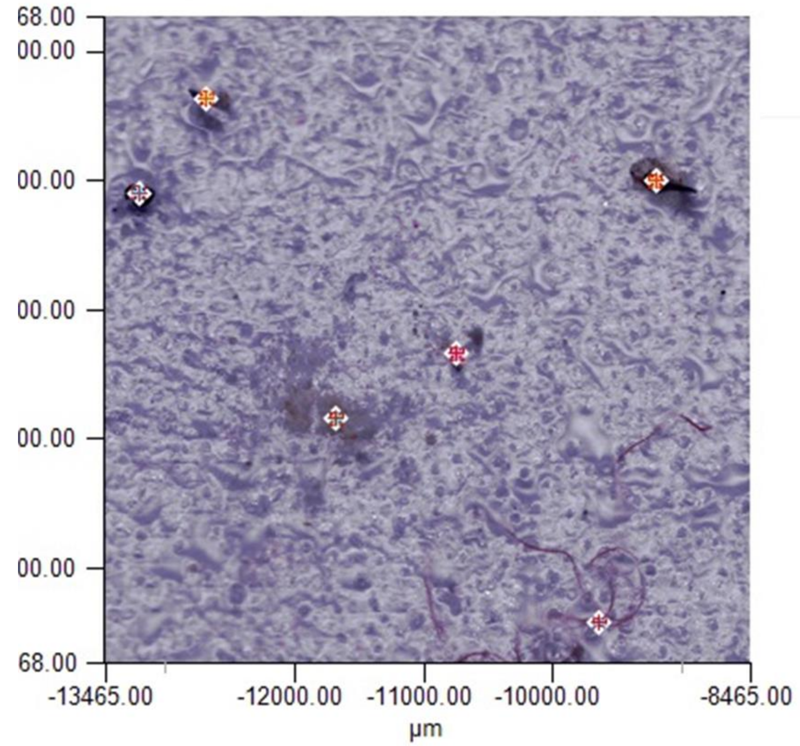

CpC52a-1,2,3

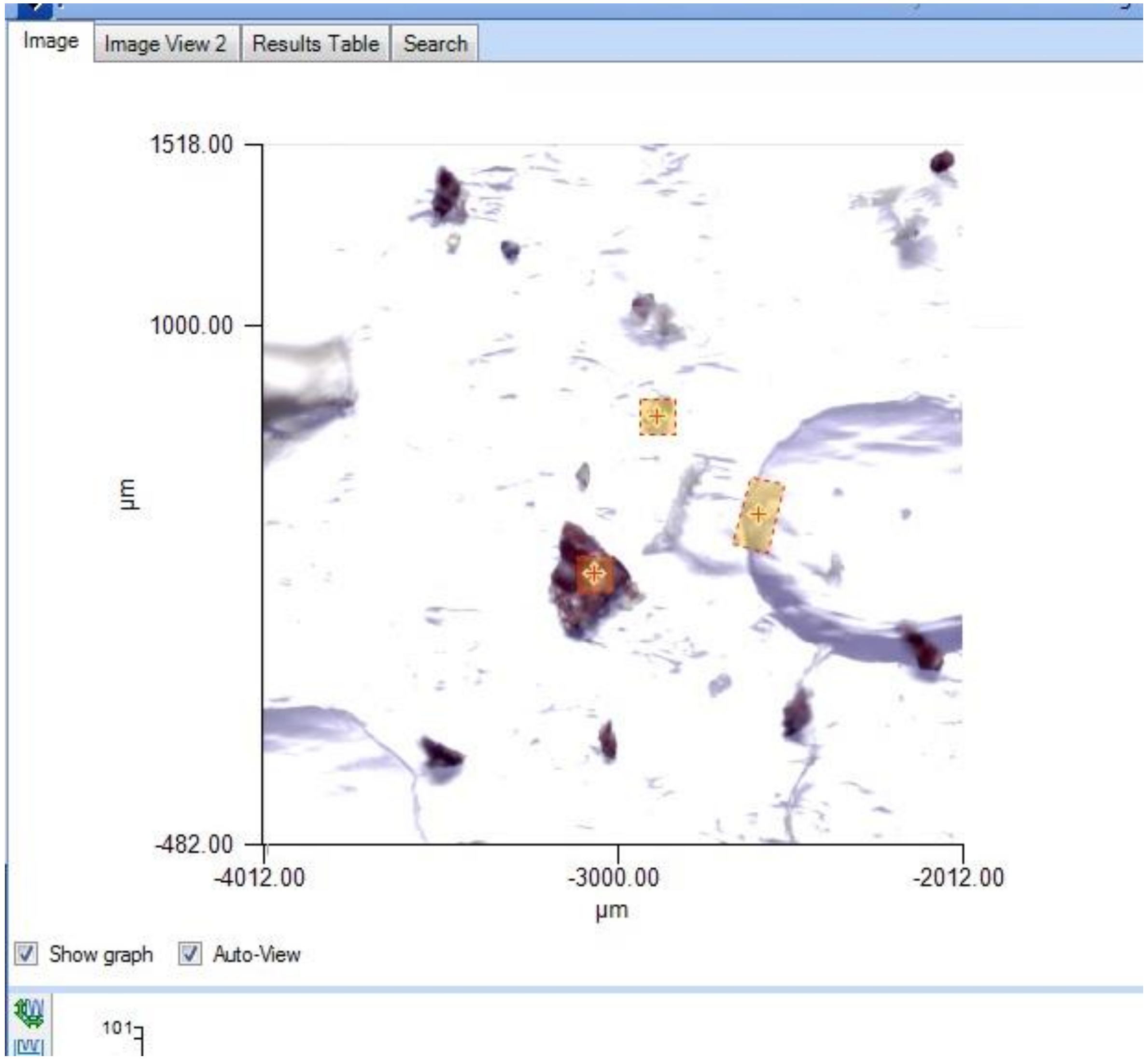

Image
Image View 2
Results Table
Search
1518.00
1000.00
-482.00
µm
-4012.00
-3000.00
-2012.00
µm
Show graph
Auto-View
101

EB1

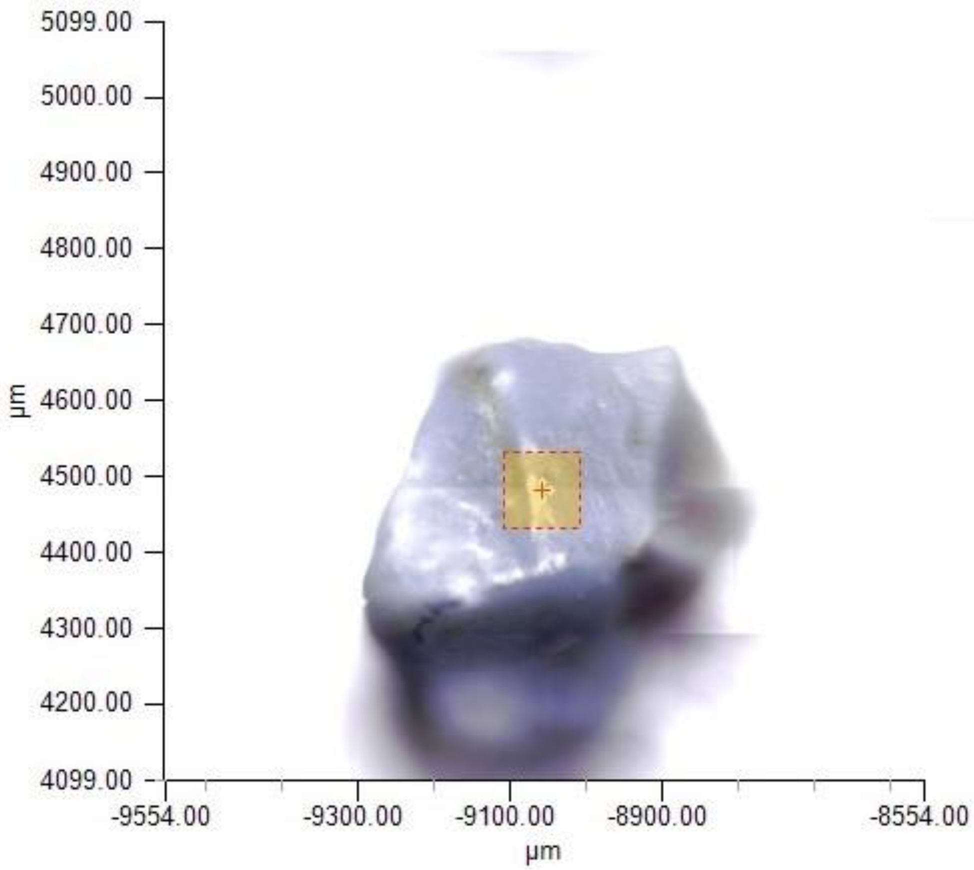
5099.00
5000.00
4900.00
4800.00
4700.00
4600.00
4500.00
4400.00
4300.00
4200.00
4099.00
µm
-9554.00
-9300.00
-9100.00
-8900.00
-8554.00
µm

EB2-1,2

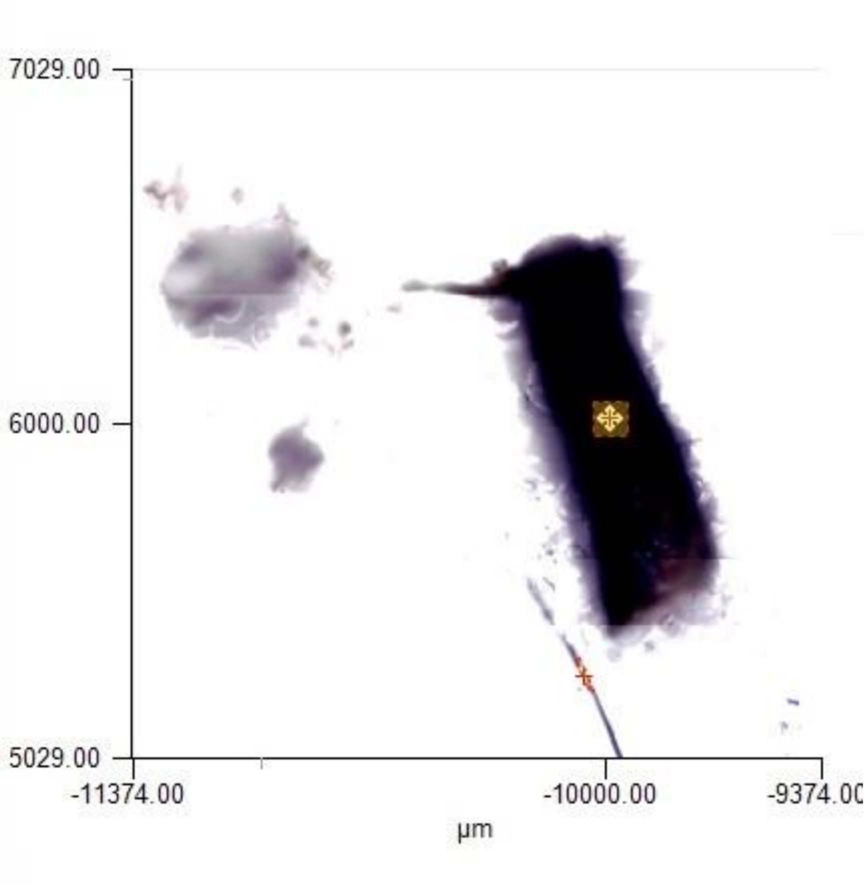

EB3

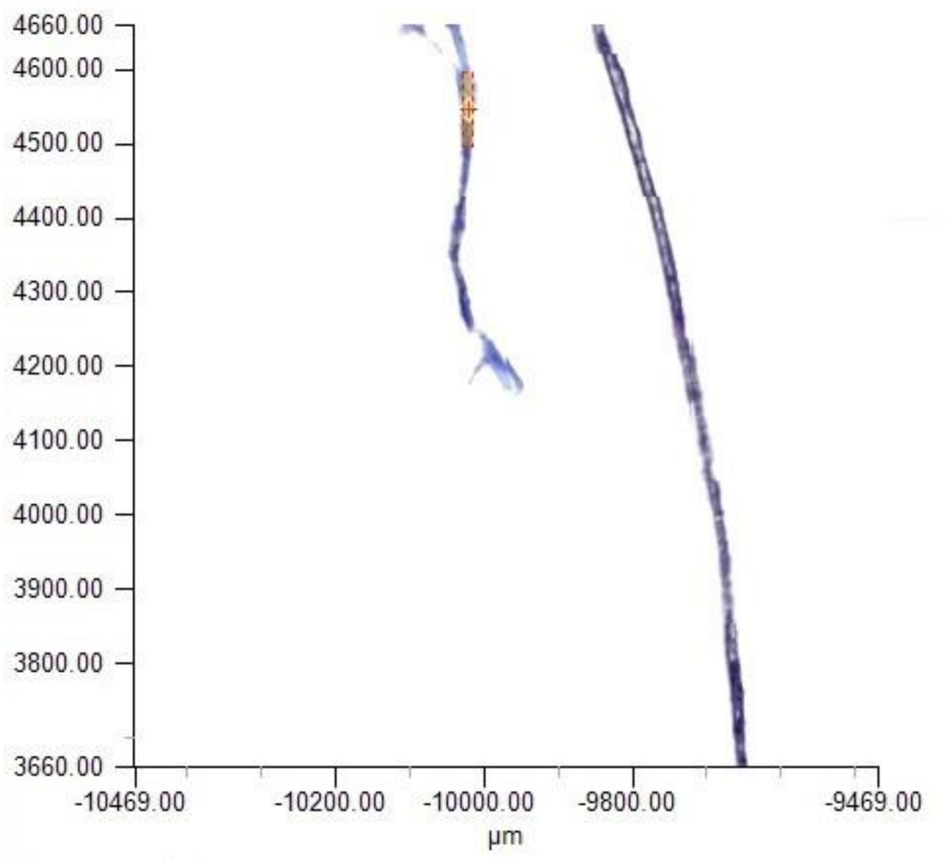

4660.00
4600.00
4500.00
4400.00
4300.00
4200.00
4100.00
4000.00
3900.00
3800.00
3660.00
-10469.00
-10200.00
-10000.00
-9800.00
-9469.00
µm

EB4

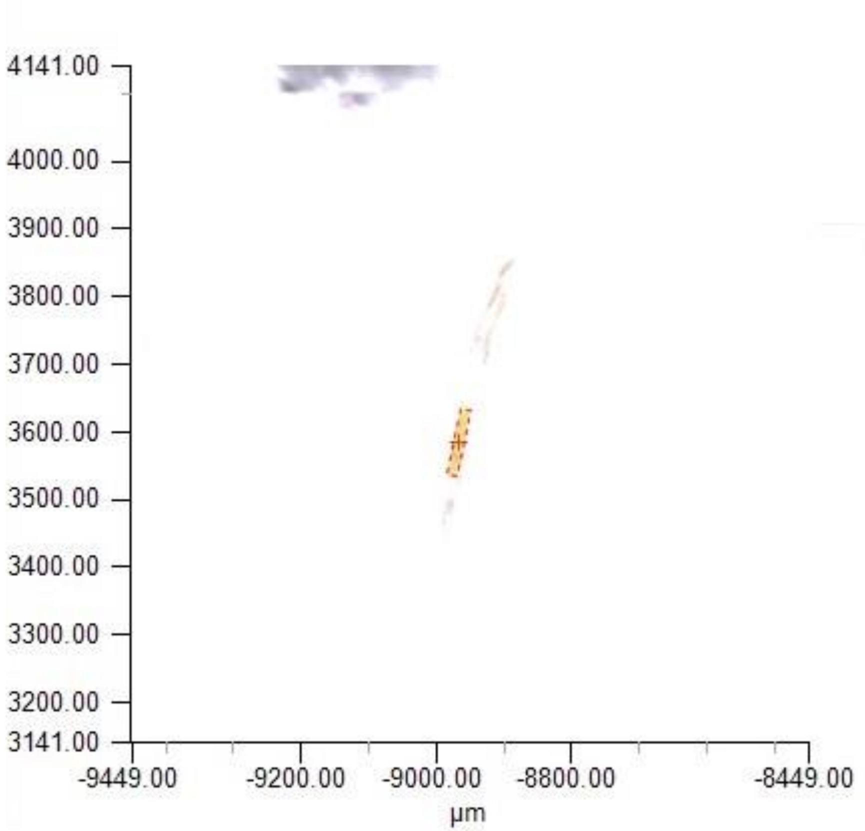
4141.00
4000.00
3900.00
3800.00
3700.00
3600.00
3500.00
3400.00
3300.00
3200.00
3141.00
-9449.00
-9200.00
-9000.00
-8800.00
-8449.00
μm

EB6-1

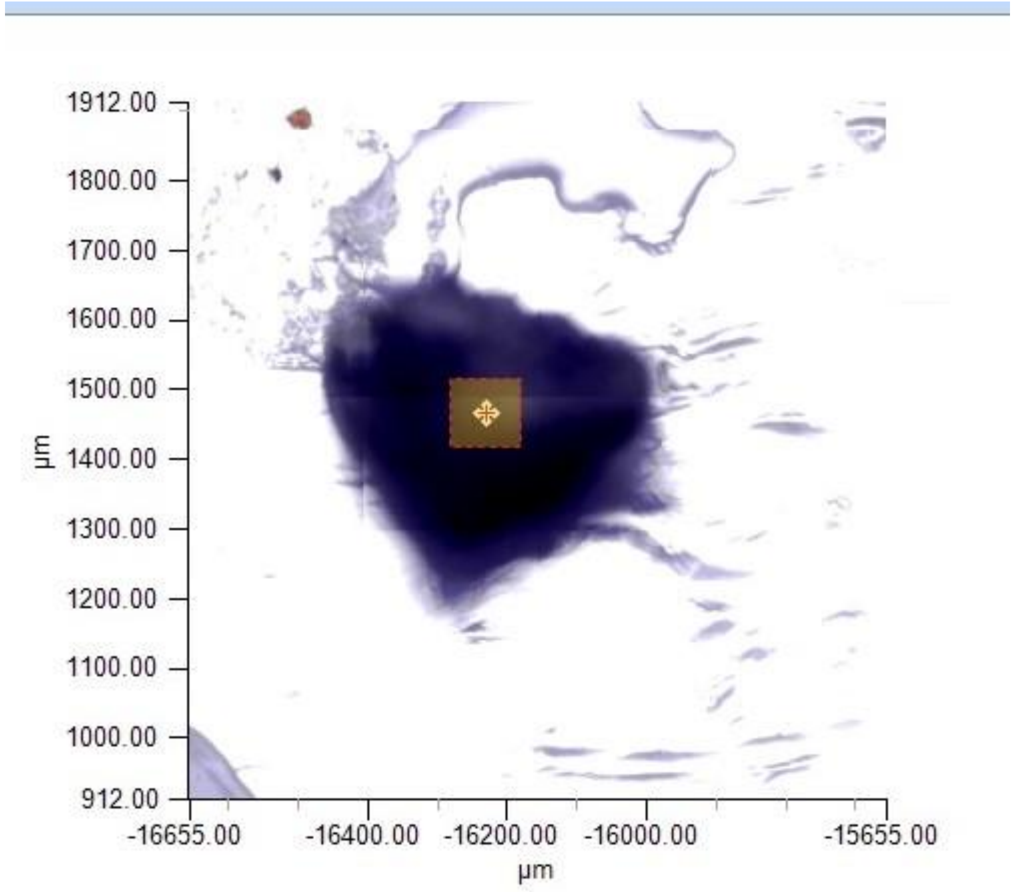
1912.00
1800.00
1700.00
1600.00
1500.00
1400.00
1300.00
1200.00
1100.00
1000.00
912.00
µm
-16655.00
-16400.00
-16200.00
-16000.00
-15655.00
µm

EB7-1

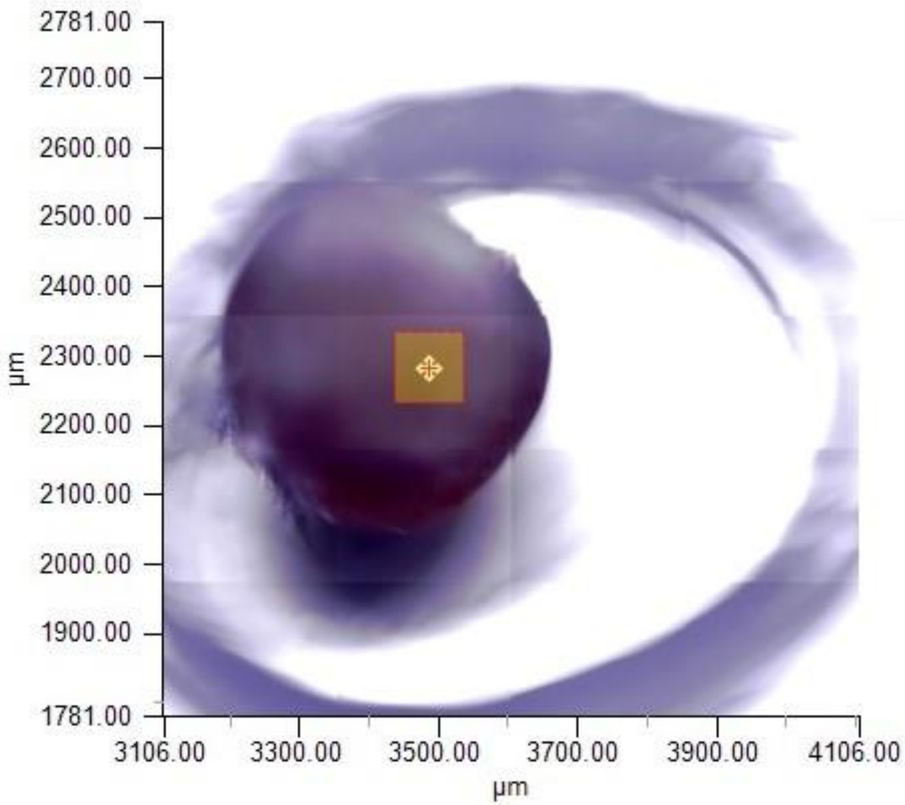
2781.00
2700.00
2600.00
2500.00
2400.00
2300.00
2200.00
2100.00
2000.00
1900.00
1781.00
µm
3106.00
3300.00
3500.00
3700.00
3900.00
4106.00
µm

EB7-2

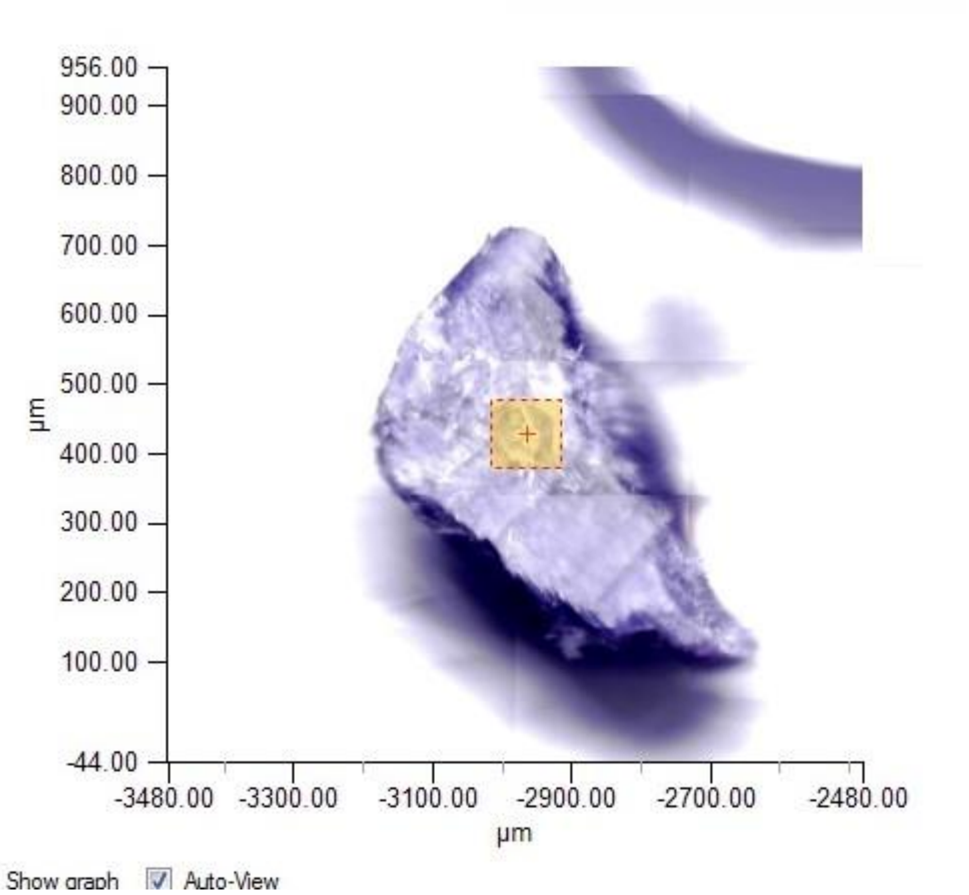
956.00
900.00
800.00
700.00
600.00
500.00
400.00
300.00
200.00
100.00
-44.00
µm
-3480.00
-3300.00
-3100.00
-2900.00
-2700.00
-2480.00
µm
Show graph
Auto-View

EB7-3

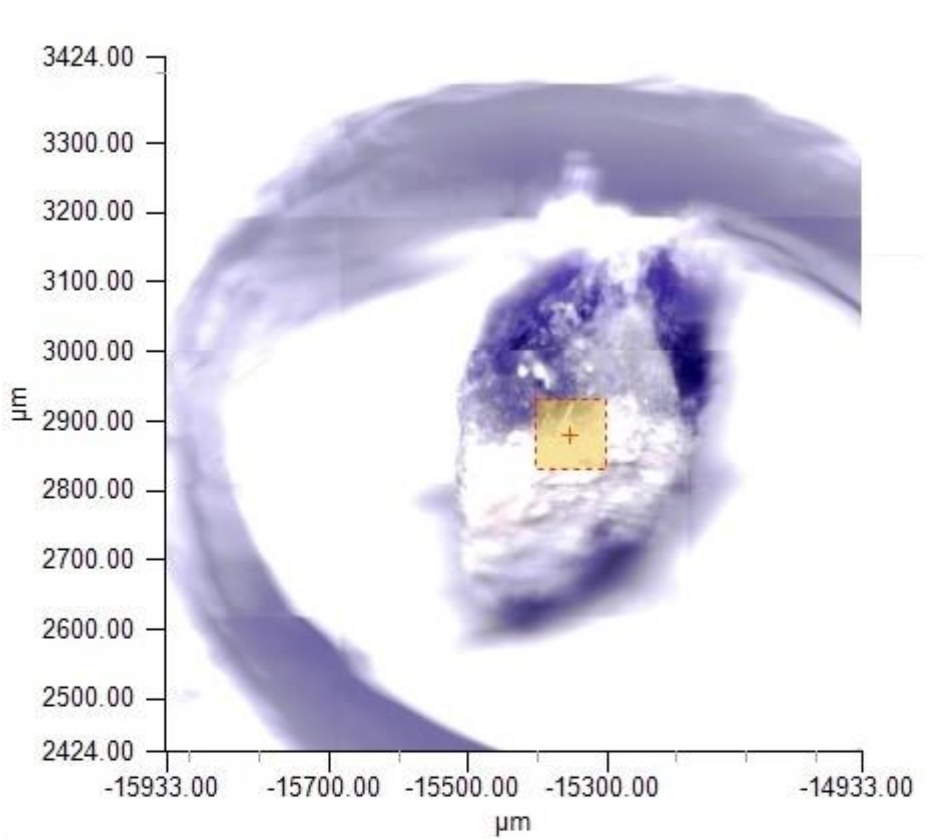

3424.00
3300.00
3200.00
3100.00
3000.00
2900.00
2800.00
2700.00
2600.00
2500.00
2424.00
µm
-15933.00
-15700.00
-15500.00
-15300.00
-14933.00
µm

EB7-4

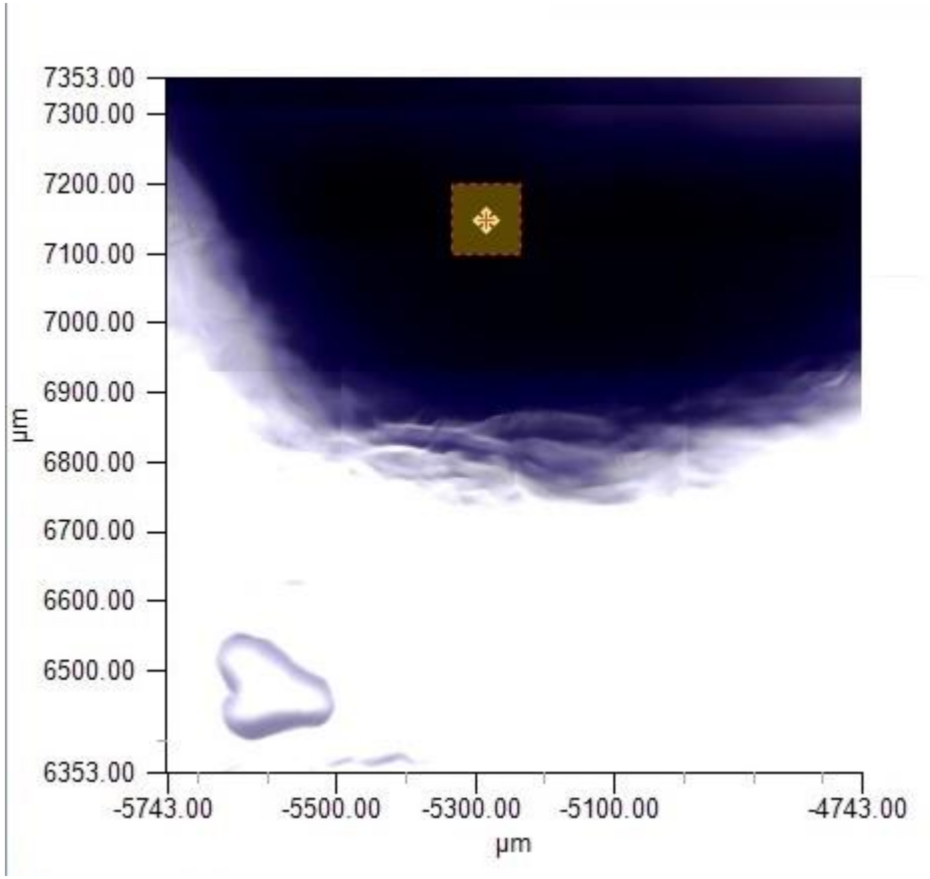

7353.00
7300.00
7200.00
7100.00
7000.00
6900.00
6800.00
6700.00
6600.00
6500.00
6353.00
µm
-5743.00
-5500.00
-5300.00
-5100.00
-4743.00
µm

EB7-5,1,2,3

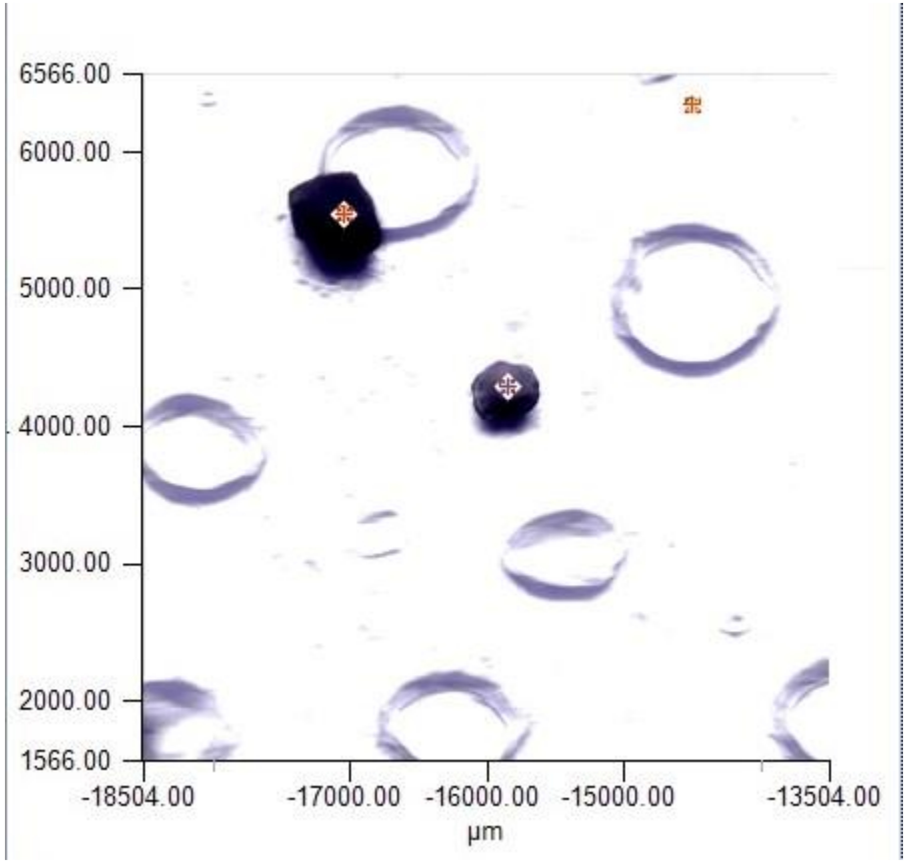
6566.00
6000.00
5000.00
4000.00
3000.00
2000.00
1566.00
-18504.00
-17000.00
-16000.00
-15000.00
-13504.00
µm

EB7-6

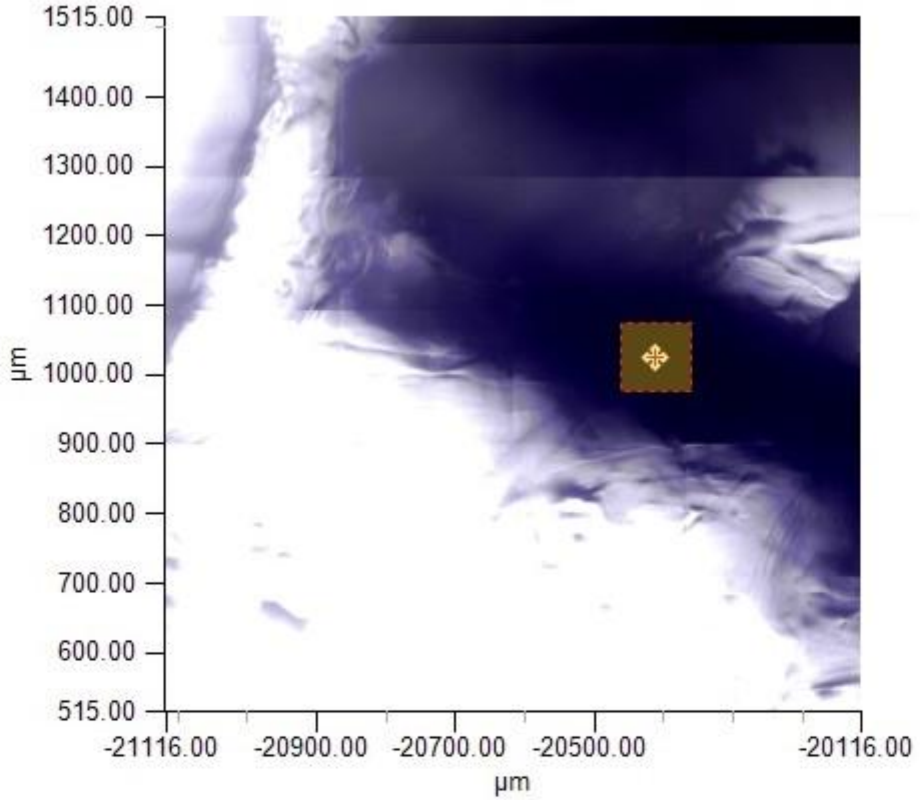
1515.00
1400.00
1300.00
1200.00
1100.00
1000.00
900.00
800.00
700.00
600.00
515.00
µm
-21116.00
-20900.00
-20700.00
-20500.00
-20116.00
µm

EB8-1,1,2

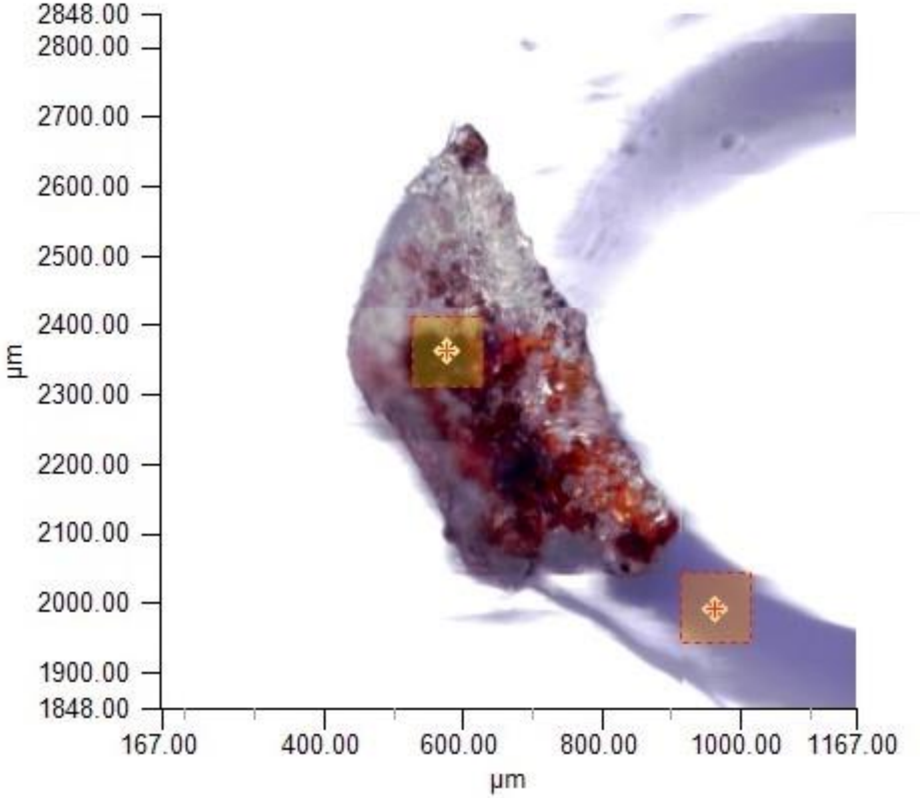

2848.00
2800.00
2700.00
2600.00
2500.00
2400.00
2300.00
2200.00
2100.00
2000.00
1900.00
1848.00
µm
167.00
400.00
600.00
800.00
1000.00
1167.00
µm

EB8-2,1,2,3,4

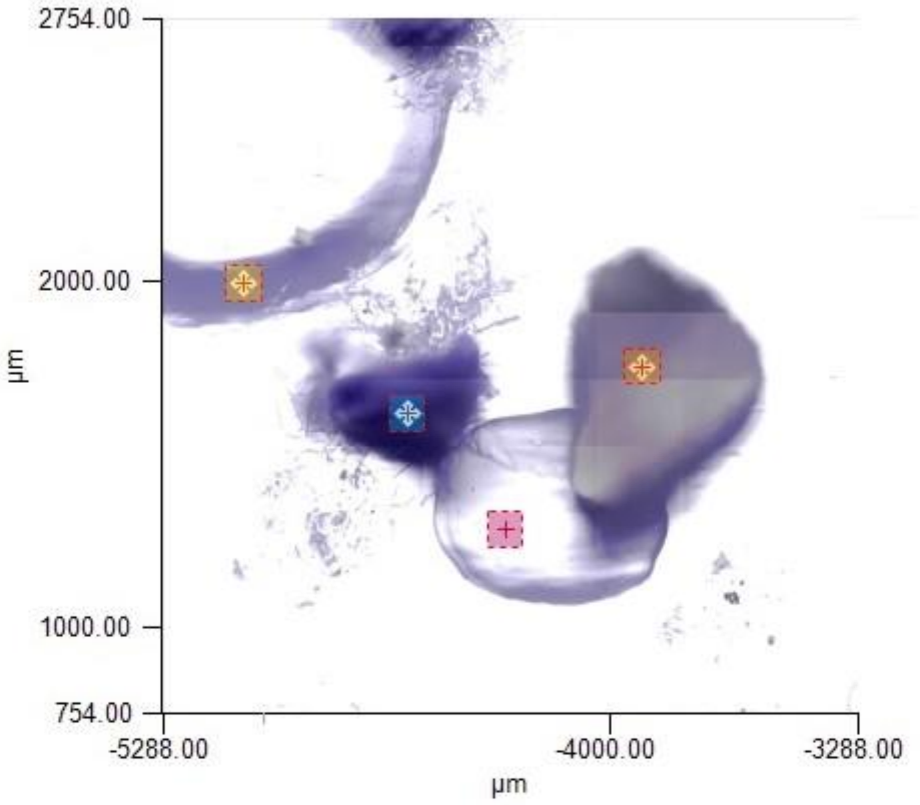

EB8-3,1,2

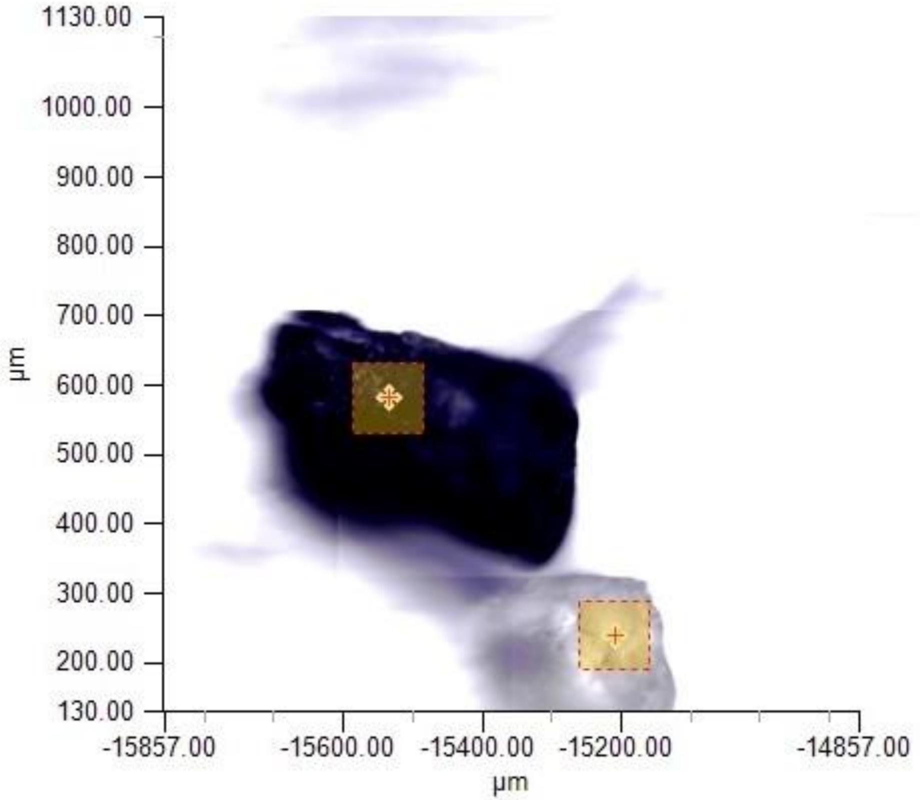
1130.00
1000.00
900.00
800.00
700.00
600.00
500.00
400.00
300.00
200.00
130.00
µm
-15857.00
-15600.00
-15400.00
-15200.00
-14857.00
µm

EB9,1

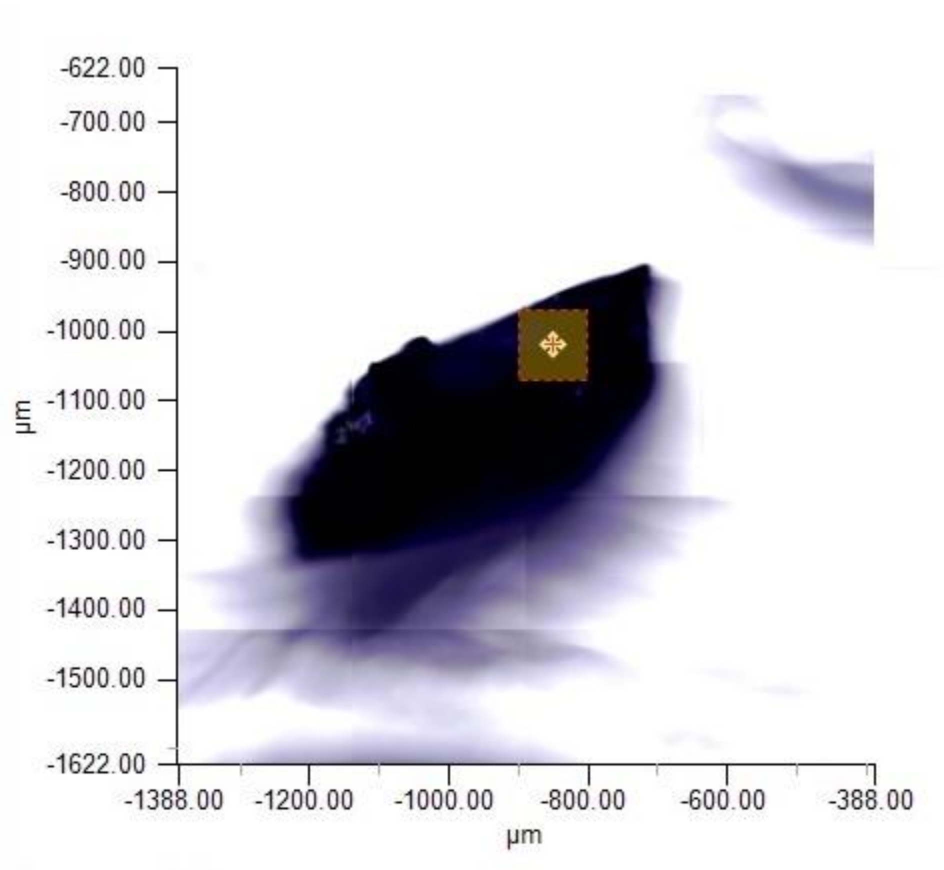

-622.00
-700.00
-800.00
-900.00
-1000.00
-1100.00
-1200.00
-1300.00
-1400.00
-1500.00
-1622.00
µm
-1388.00
-1200.00
-1000.00
-800.00
-600.00
-388.00
µm

EB9,2

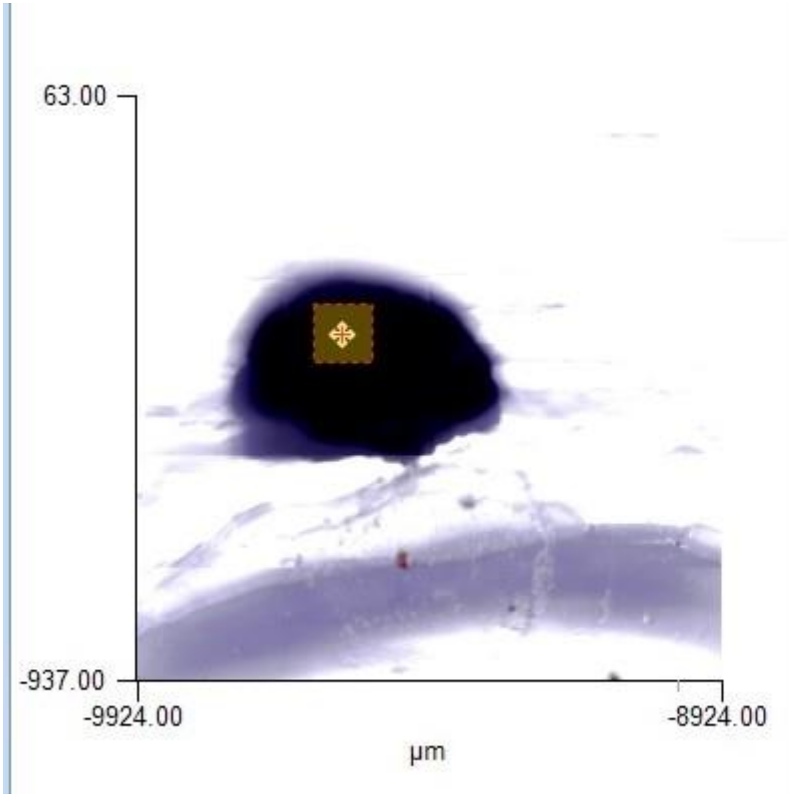

EB9,3

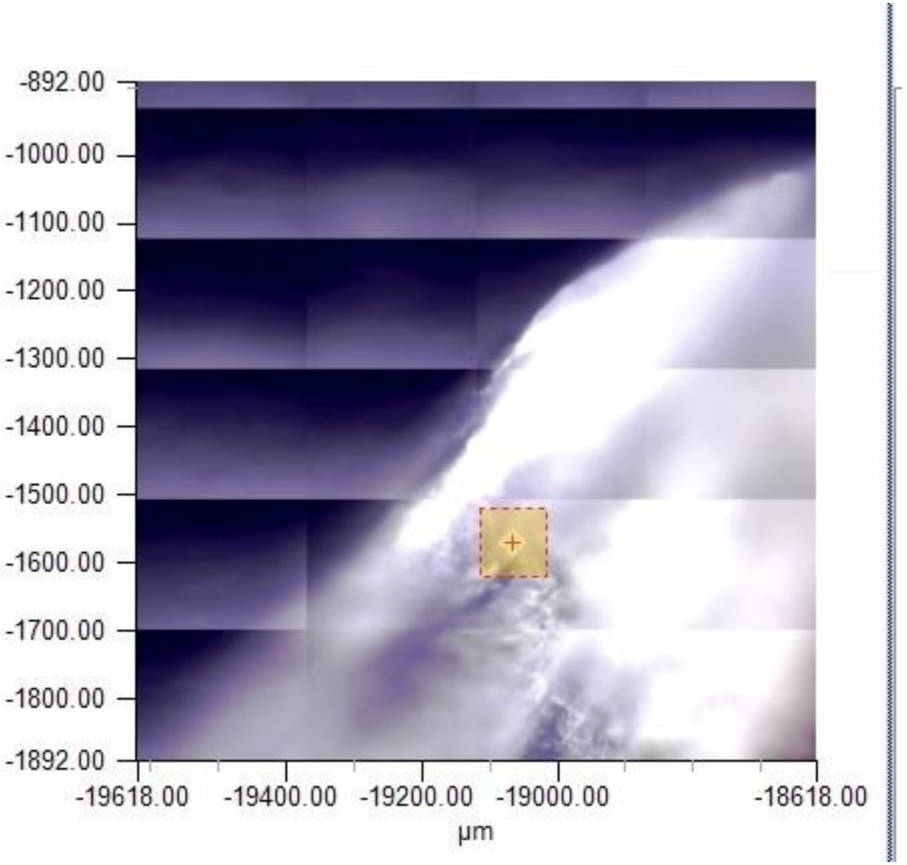

-892.00
-1000.00
-1100.00
-1200.00
-1300.00
-1400.00
-1500.00
-1600.00
-1700.00
-1800.00
-1892.00
-19618.00
-19400.00
-19200.00
-19000.00
-18618.00
µm

Jericho 1-1,2,3,4,5,6

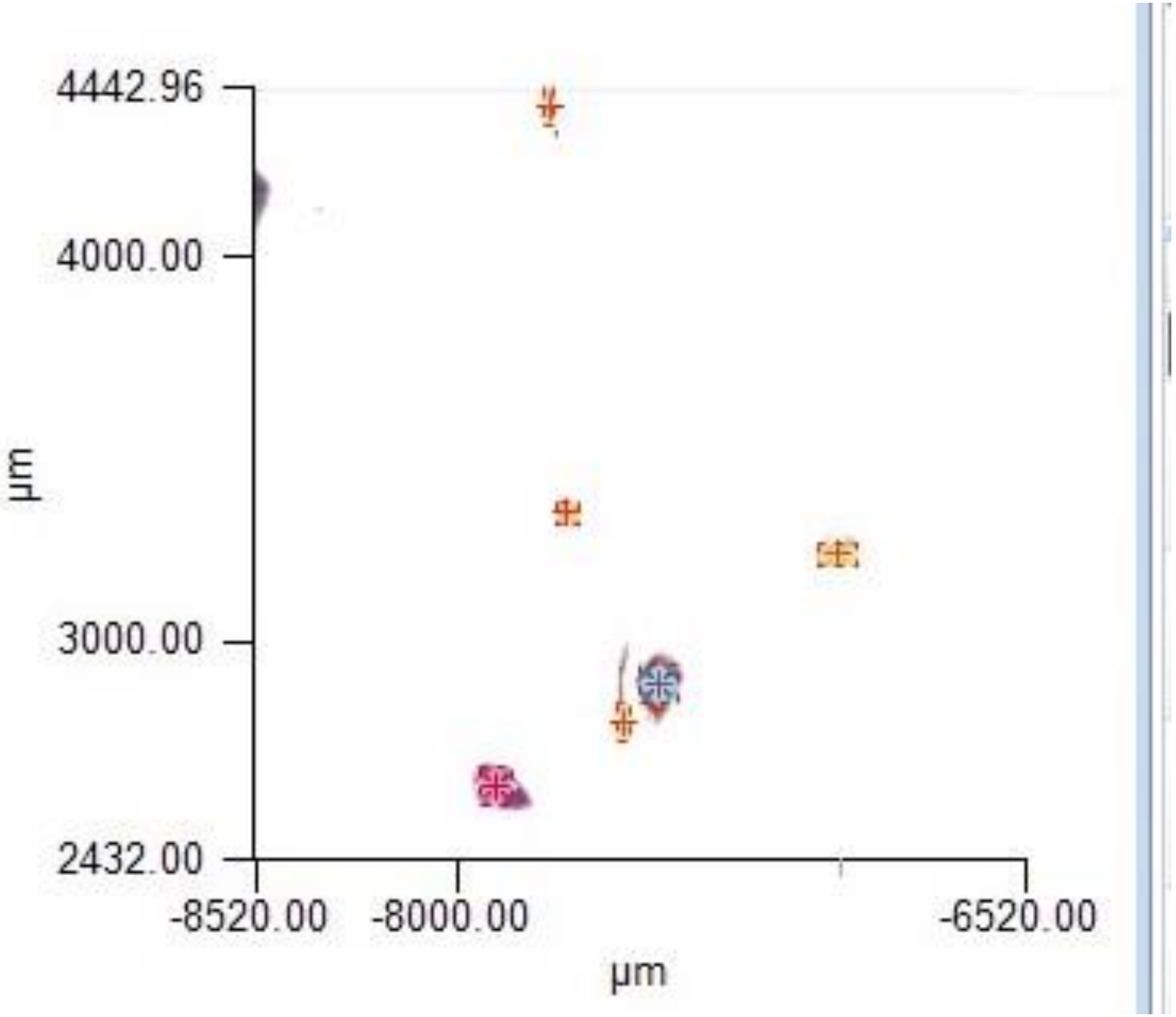

JVC1-21,22

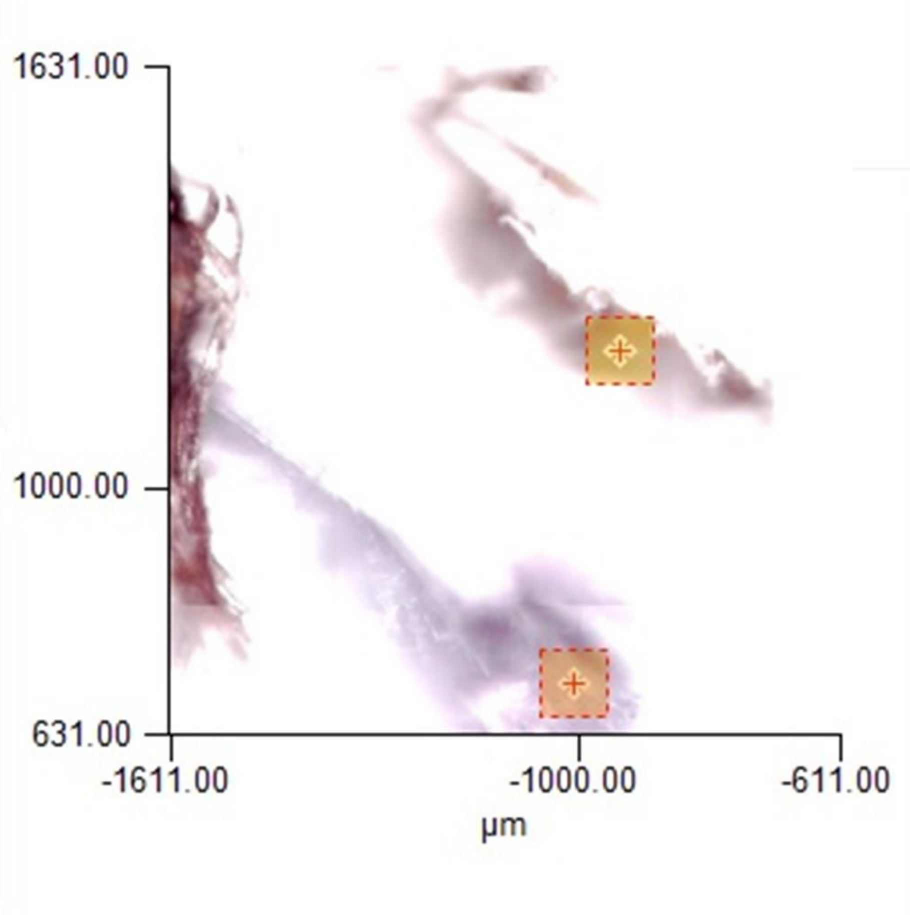

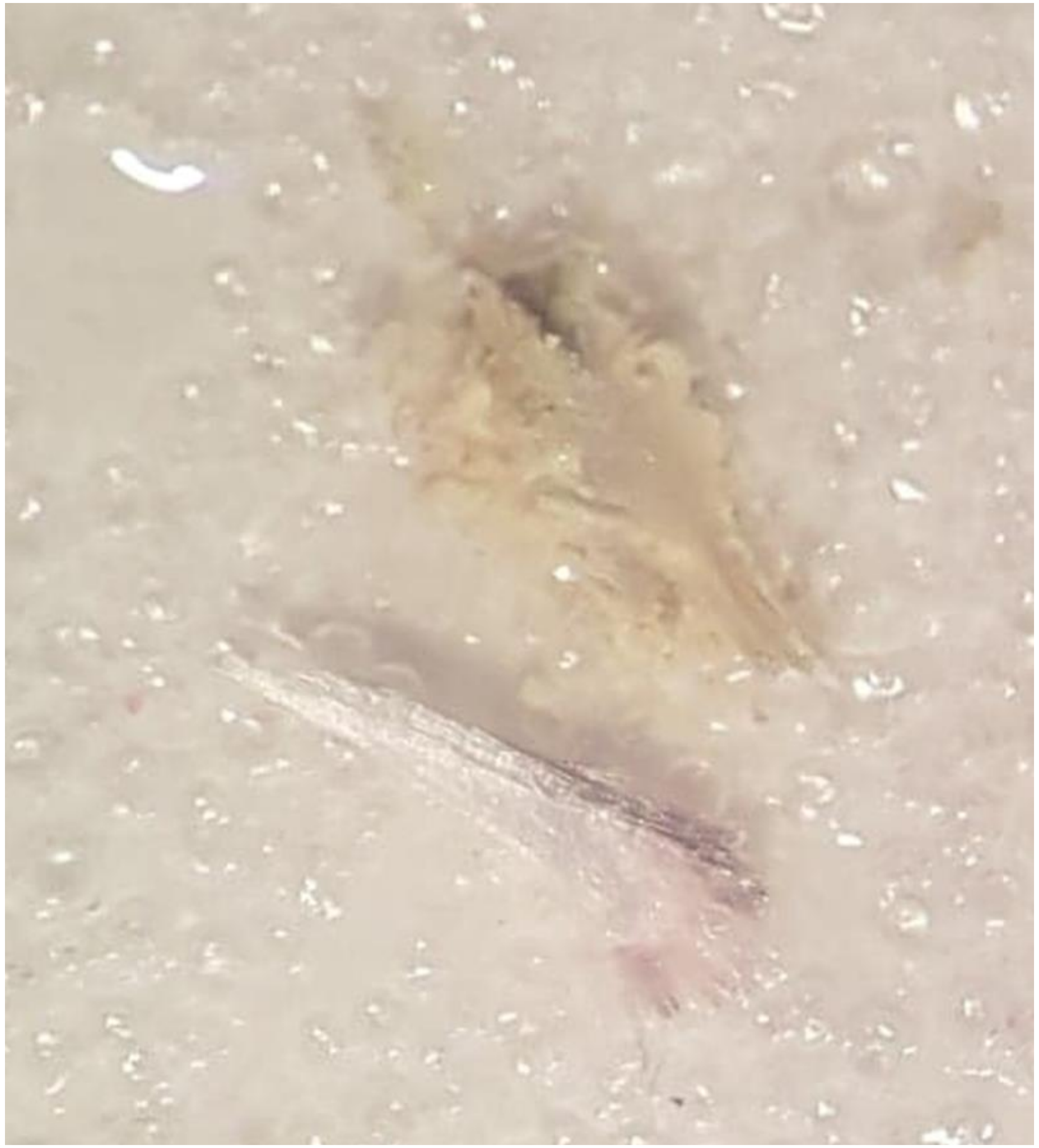

JVC1a

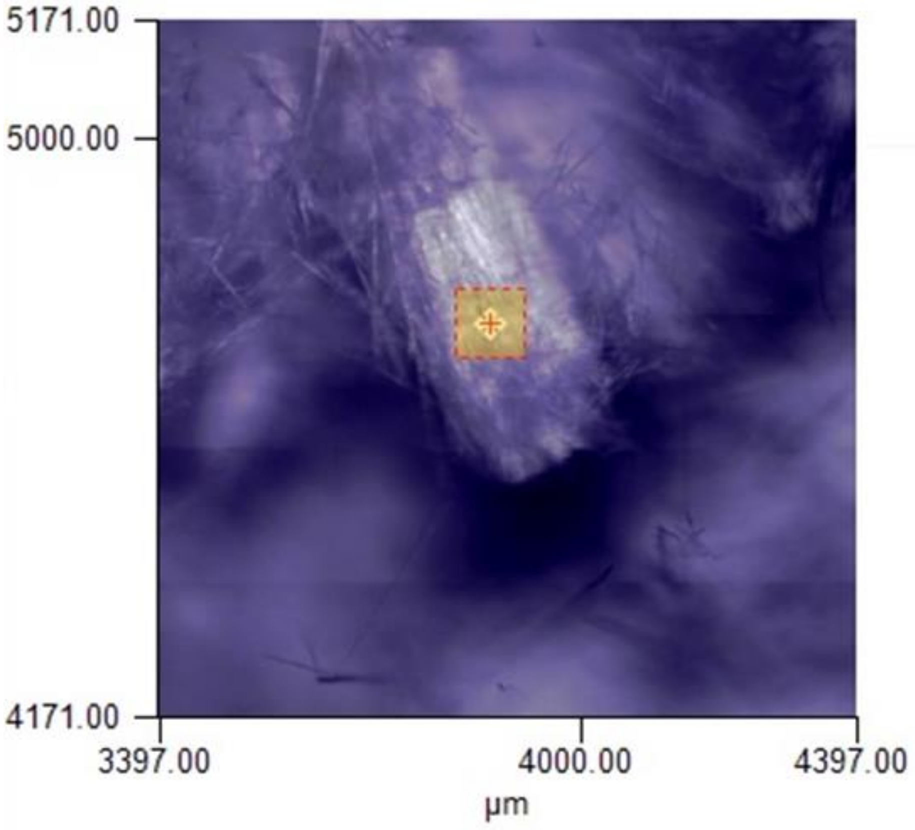

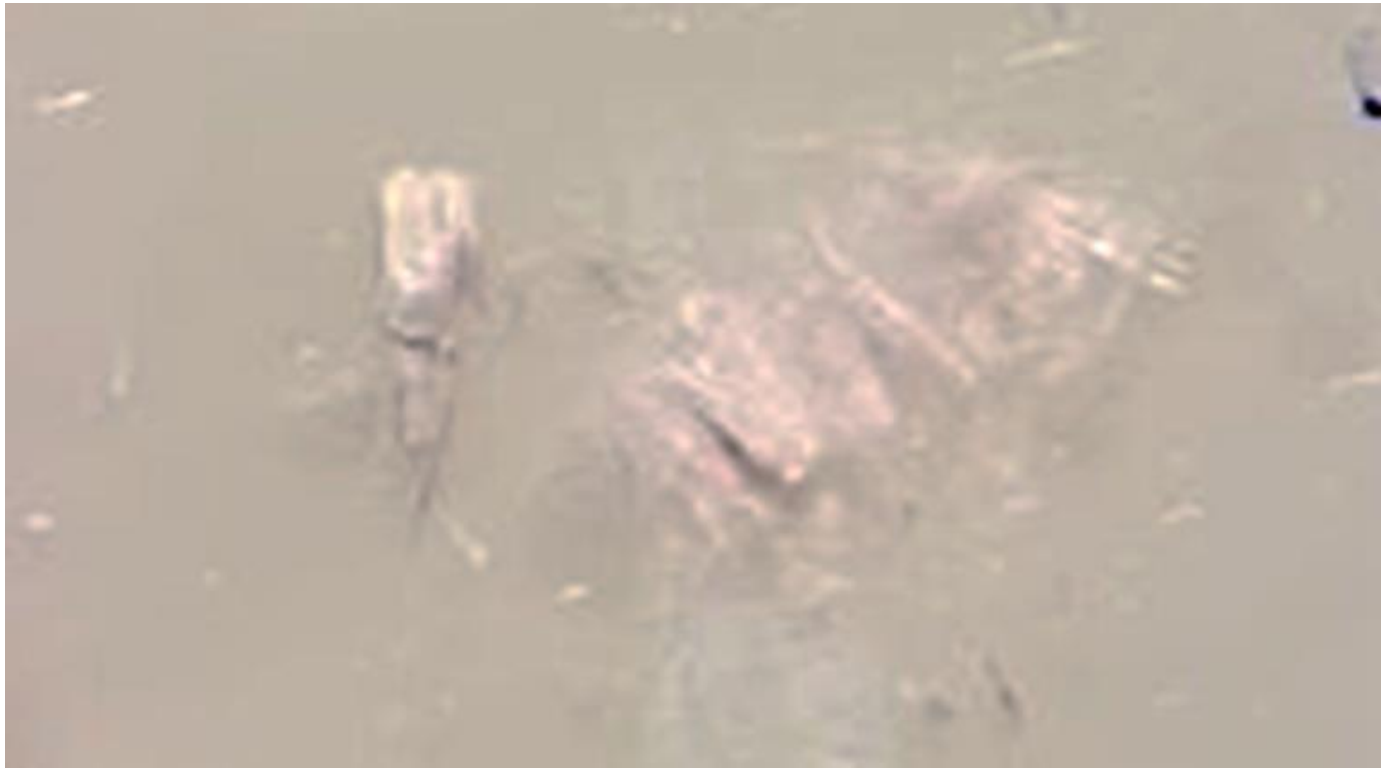

JVC-1-31,32

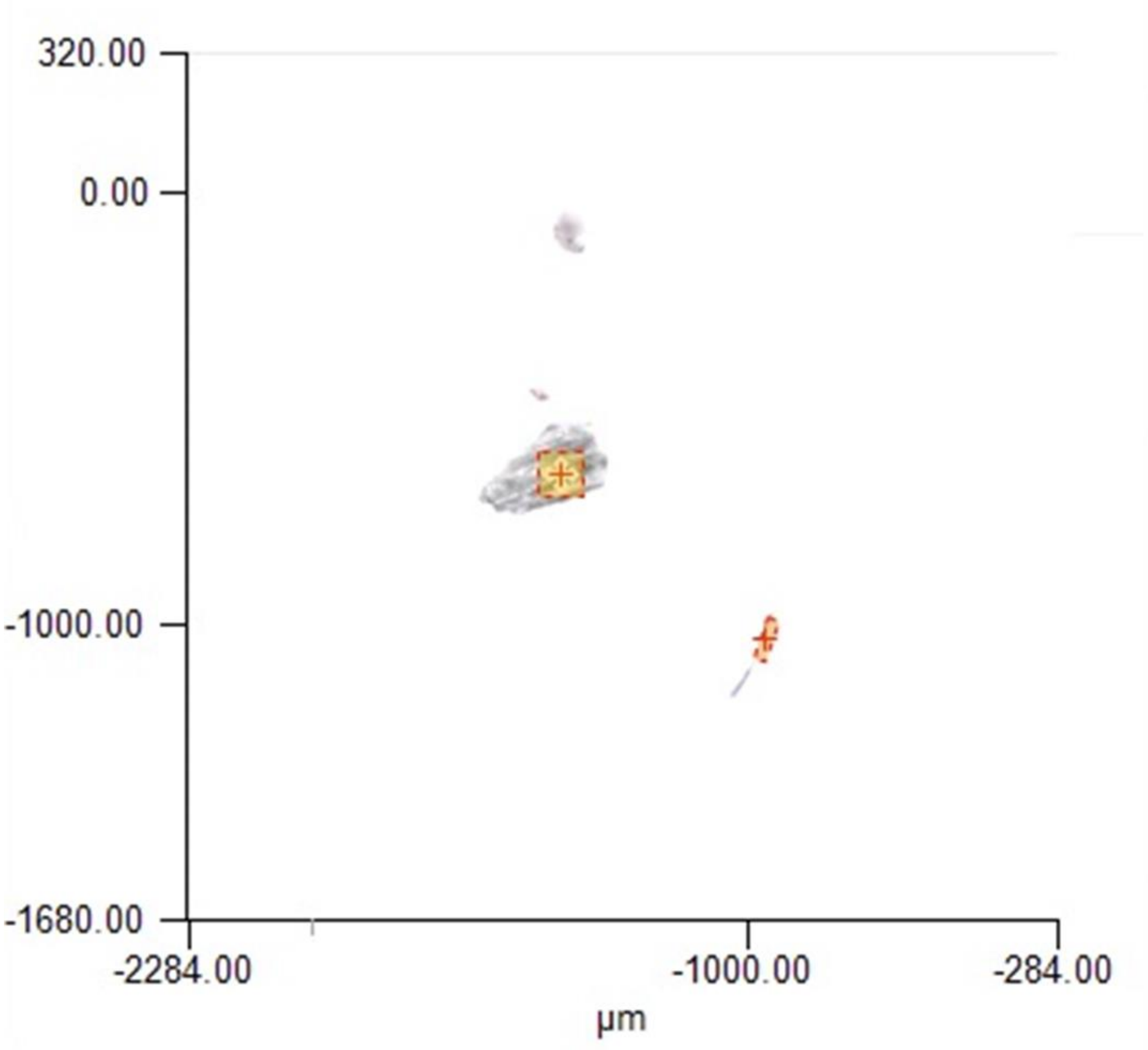

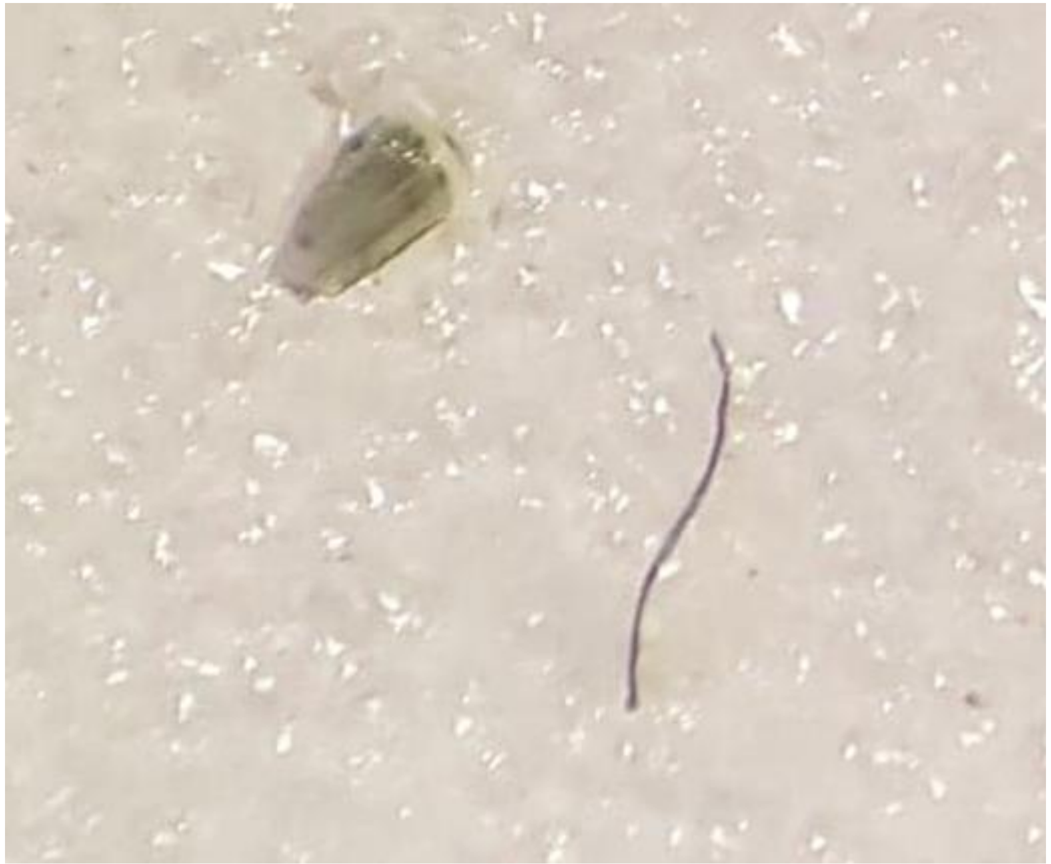

Jericho 2-1,2

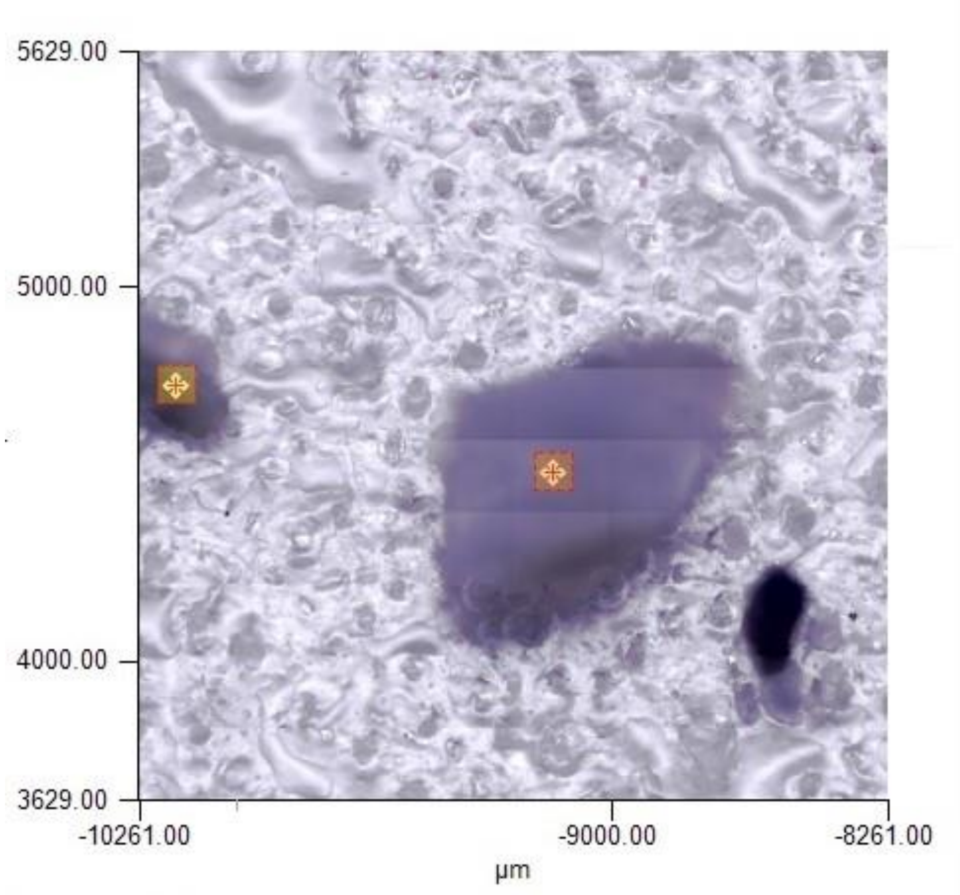

JMC3-1,2,3,4,5

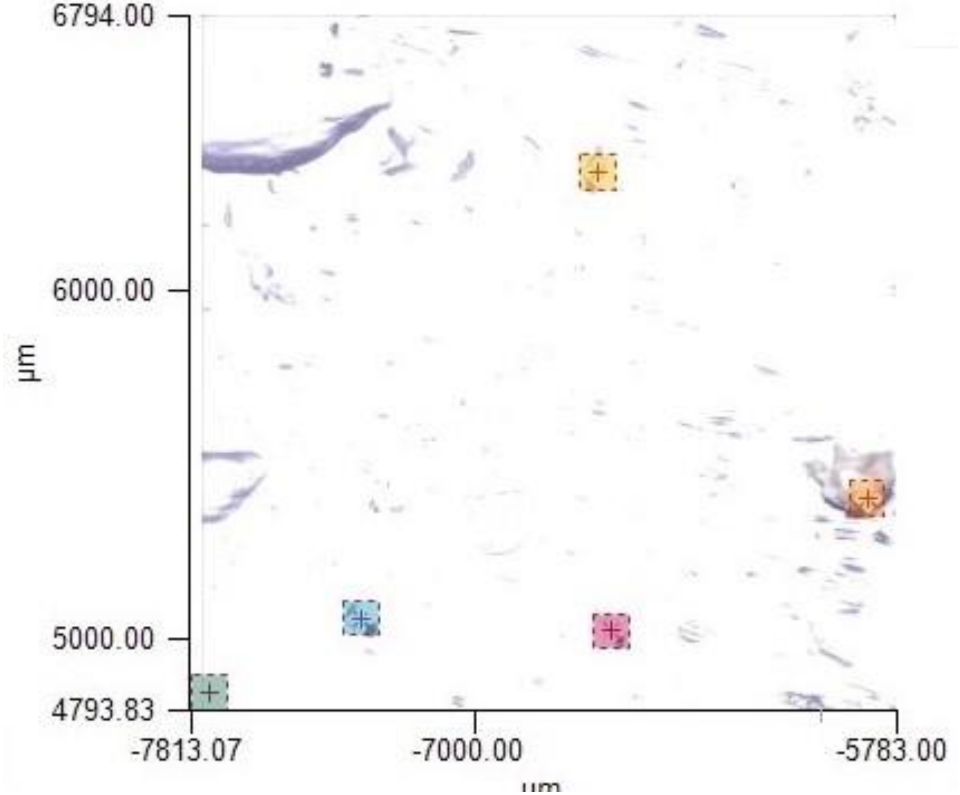

JMC4-1,2

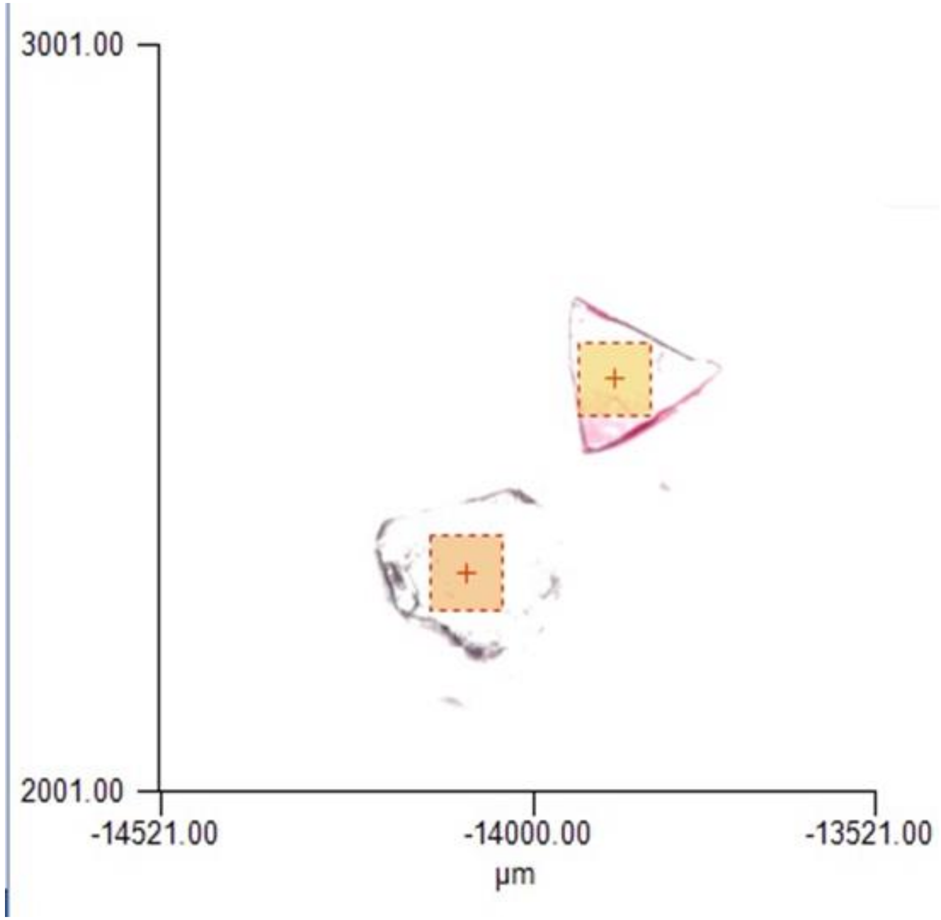

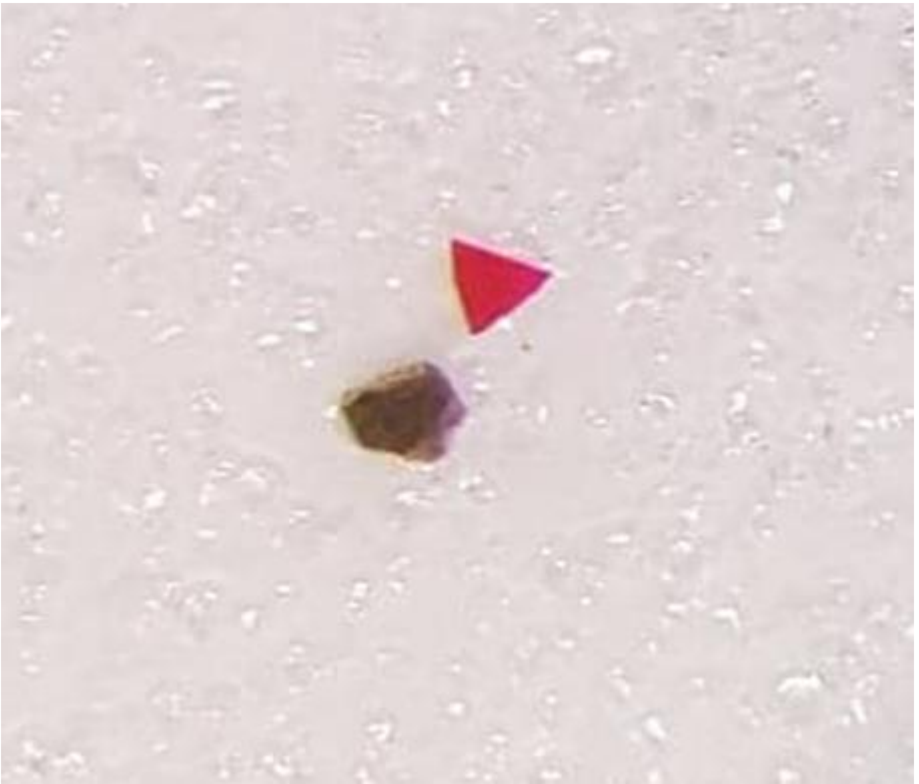

JC72-1,2,3,4,5,6

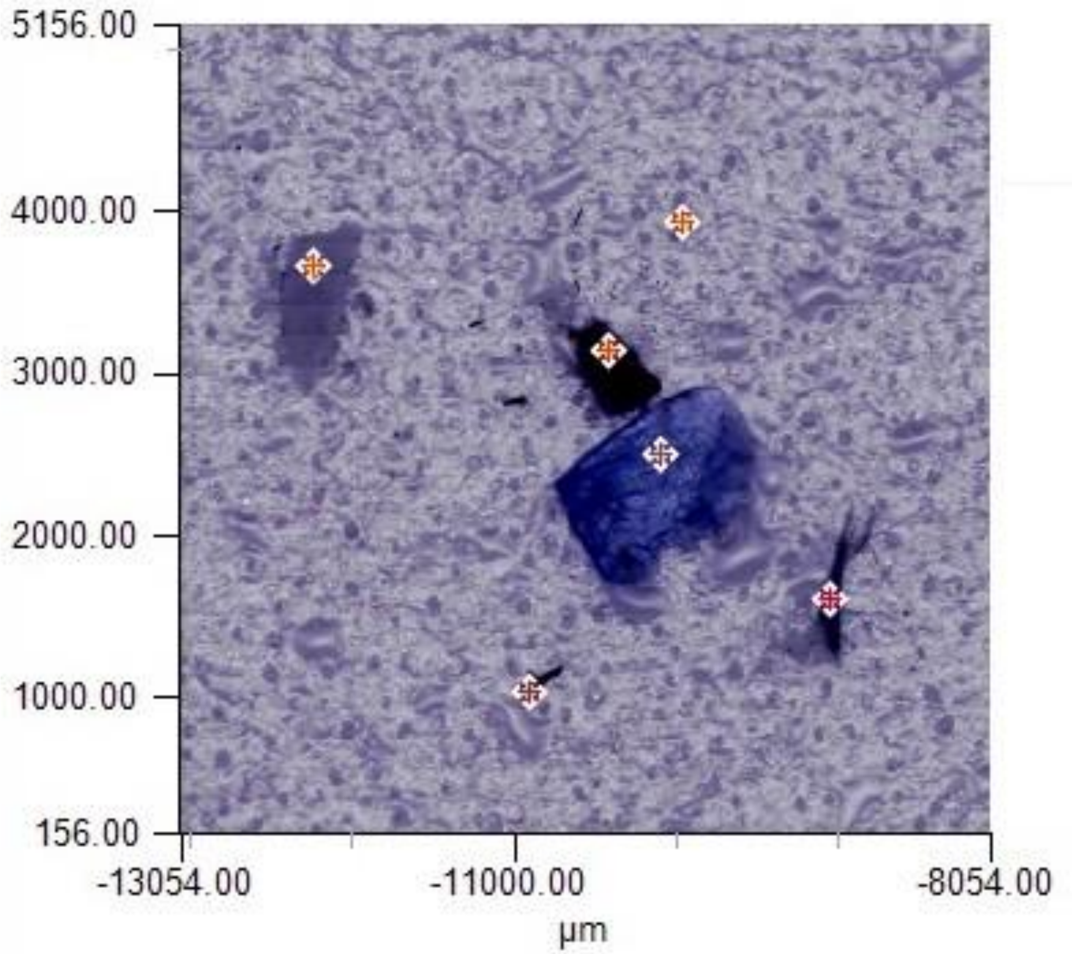
5156.00
4000.00
3000.00
2000.00
1000.00
156.00
-13054.00
-11000.00
-8054.00
µm

MetMC1-1,2

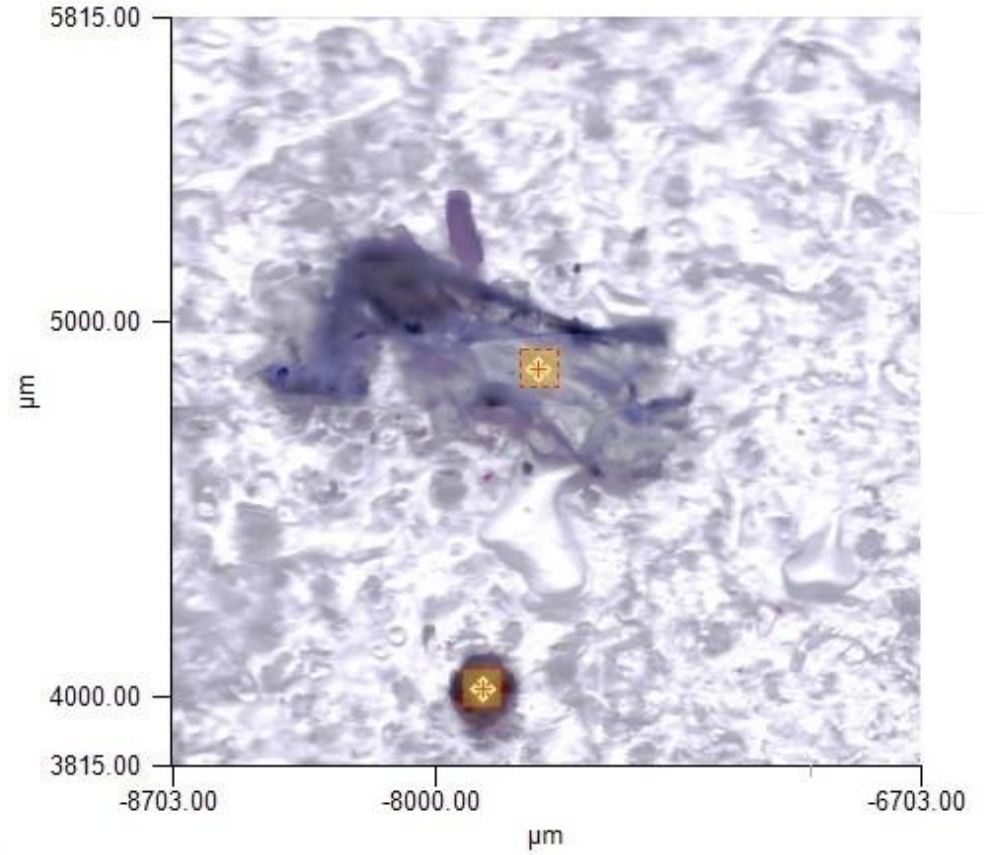

MetMC1-2,1

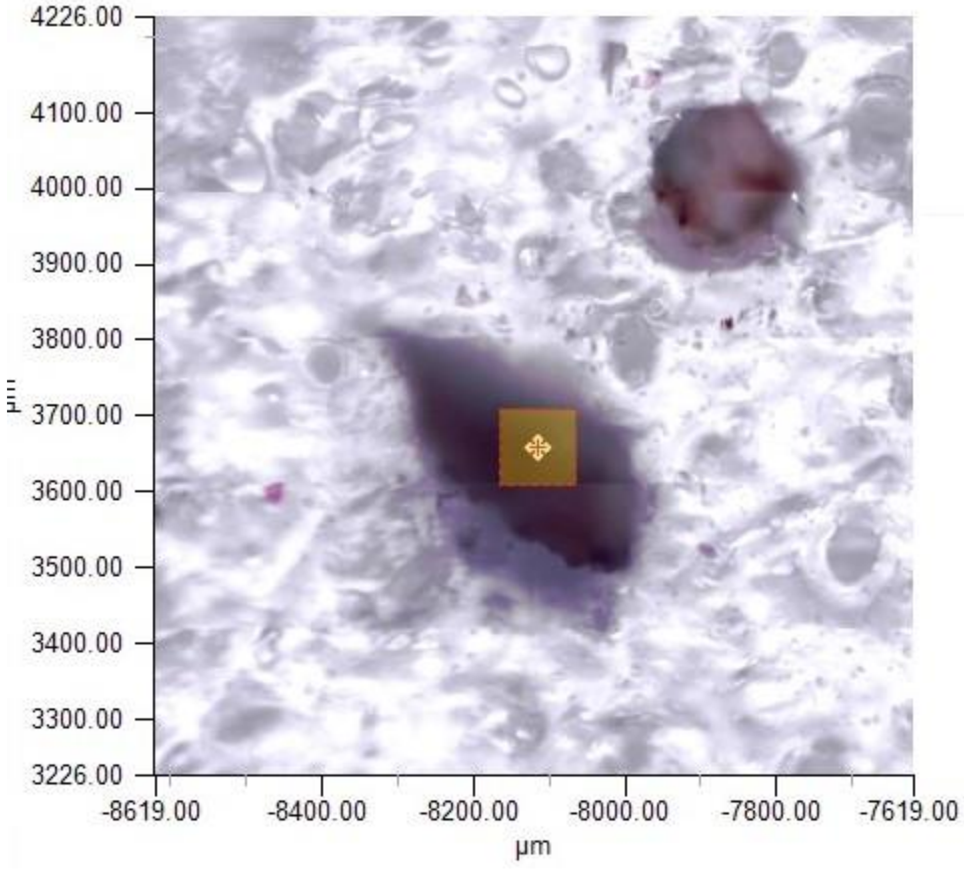
4226.00
4100.00
4000.00
3900.00
3800.00
3700.00
3600.00
3500.00
3400.00
3300.00
3226.00
-8619.00
-8400.00
-8200.00
-8000.00
-7800.00
-7619.00
µm

MetMC4-1

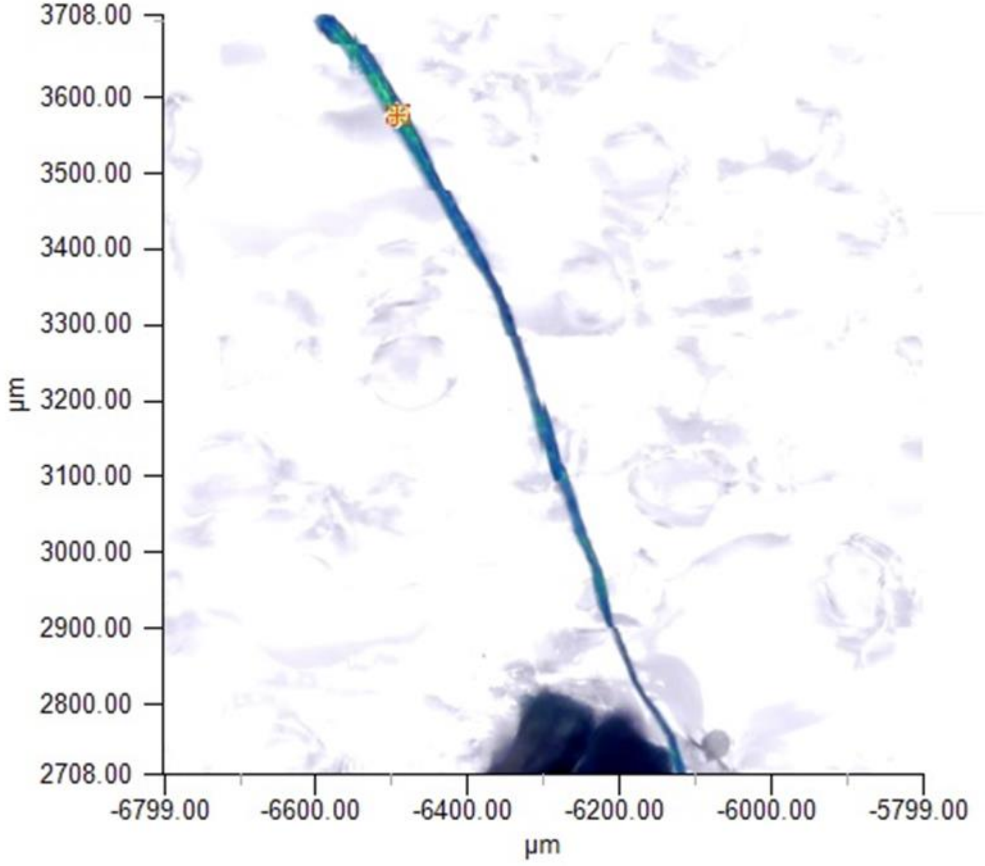
3708.00
3600.00
3500.00
3400.00
3300.00
3200.00
3100.00
3000.00
2900.00
2800.00
2708.00
µm
-6799.00
-6600.00
-6400.00
-6200.00
-6000.00
-5799.00
µm

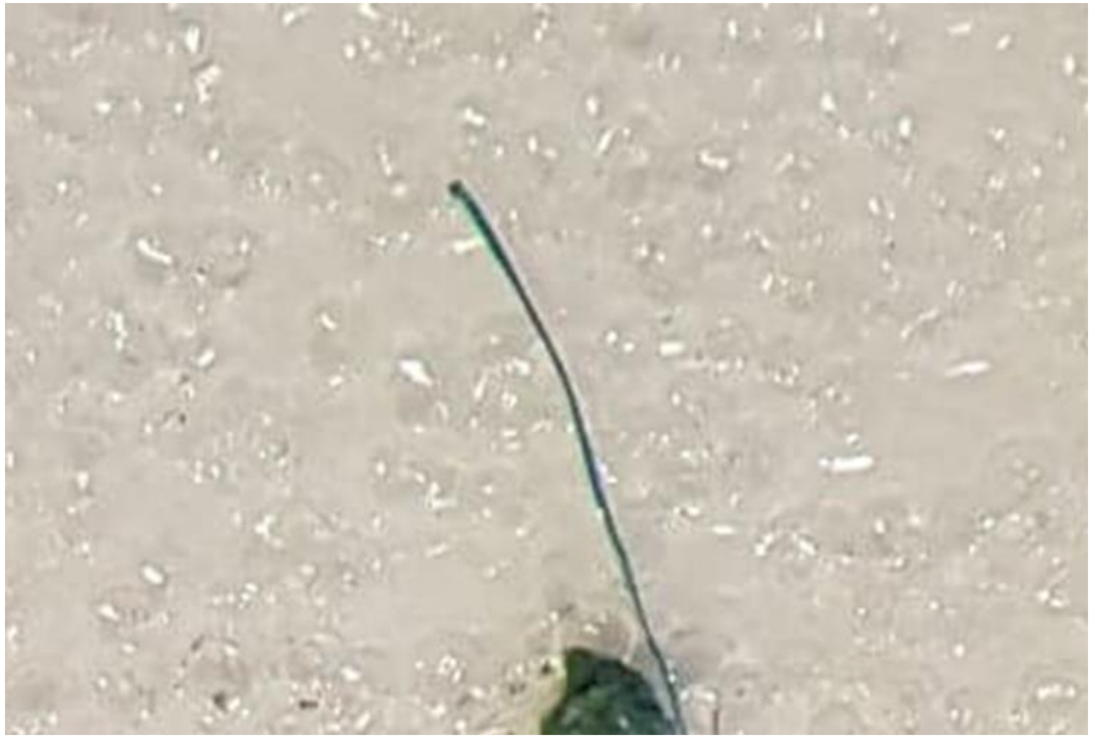

MetMC4-2

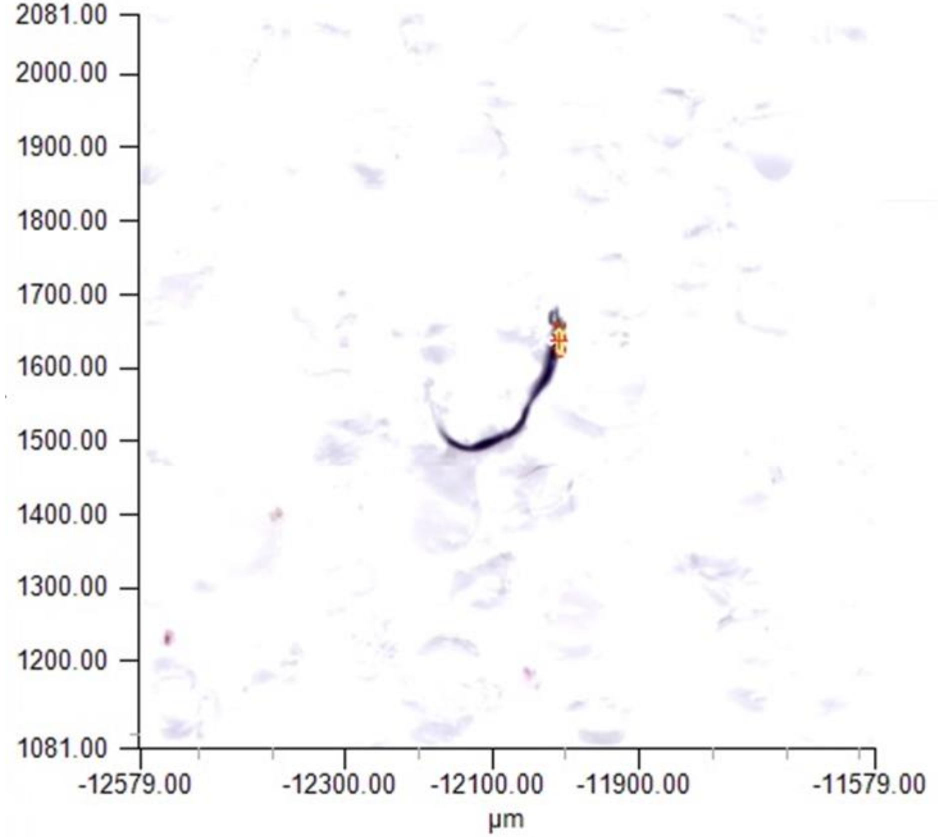

2081.00
2000.00
1900.00
1800.00
1700.00
1600.00
1500.00
1400.00
1300.00
1200.00
1081.00
-12579.00
-12300.00
-12100.00
-11900.00
-11579.00
μm

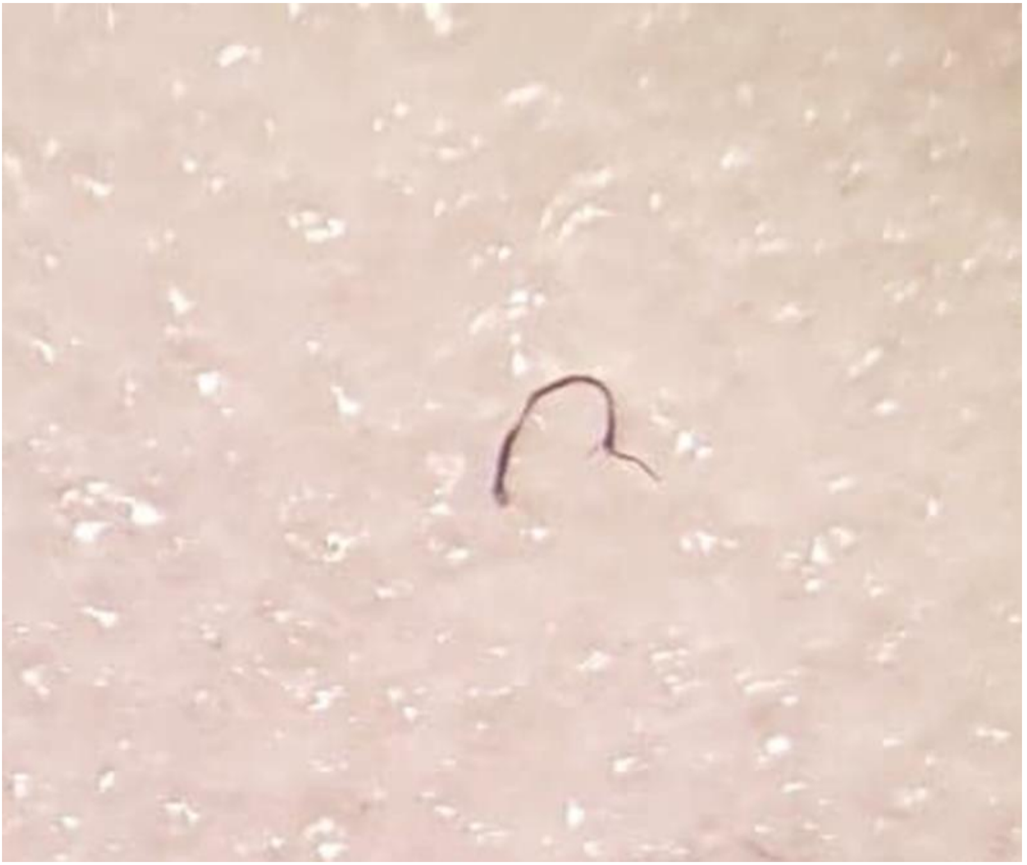

MetC4-3;1,2

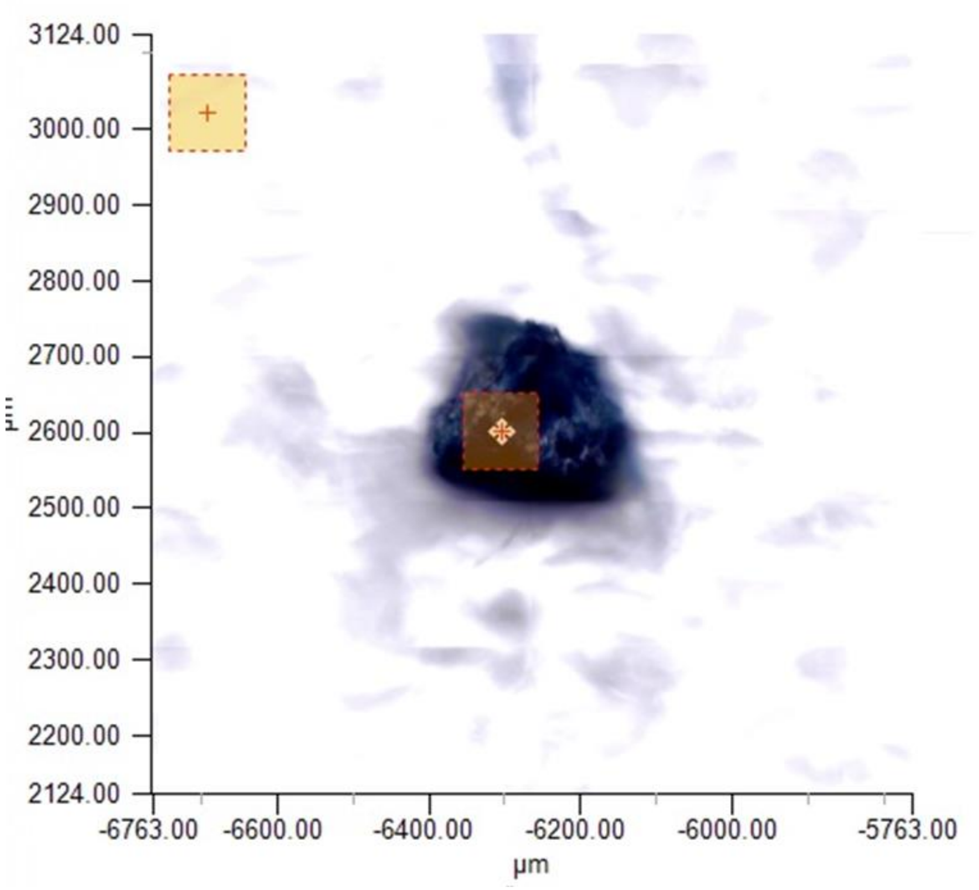
3124.00
3000.00
2900.00
2800.00
2700.00
2600.00
2500.00
2400.00
2300.00
2200.00
2124.00
-6763.00
-6600.00
-6400.00
-6200.00
-6000.00
-5763.00
μm

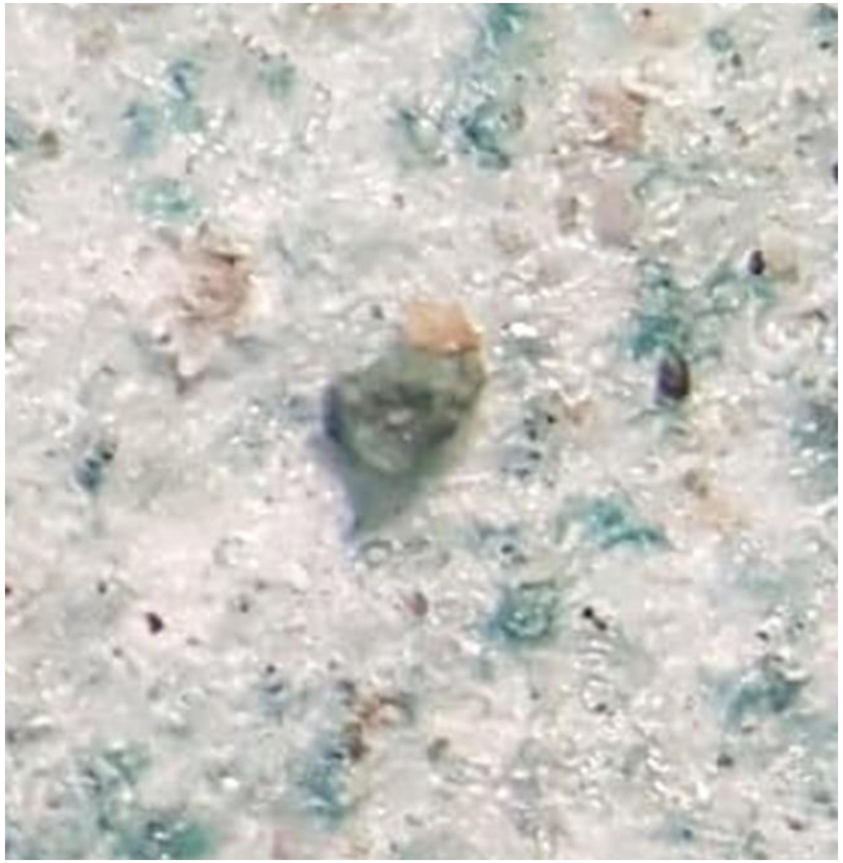

MetC4-1;1,2,3,4,5

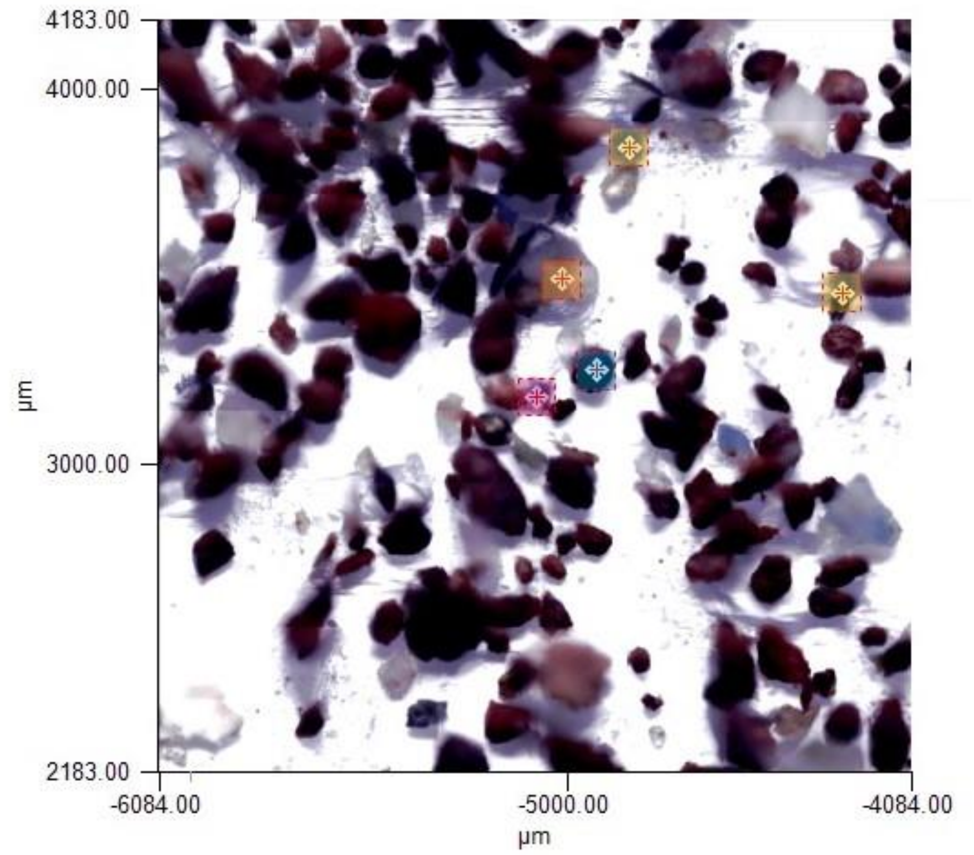

MetC5-1;1,2

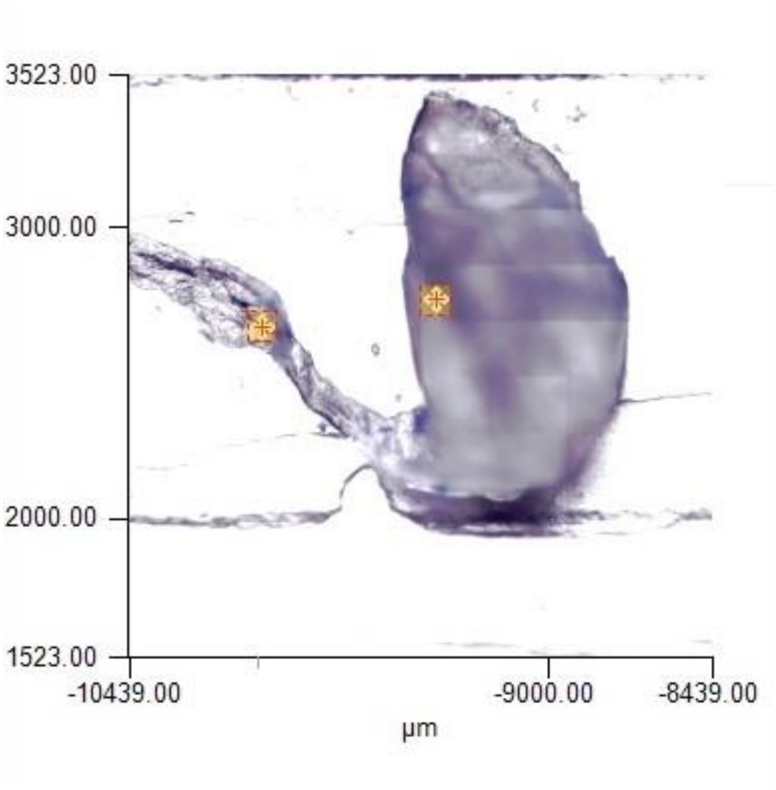

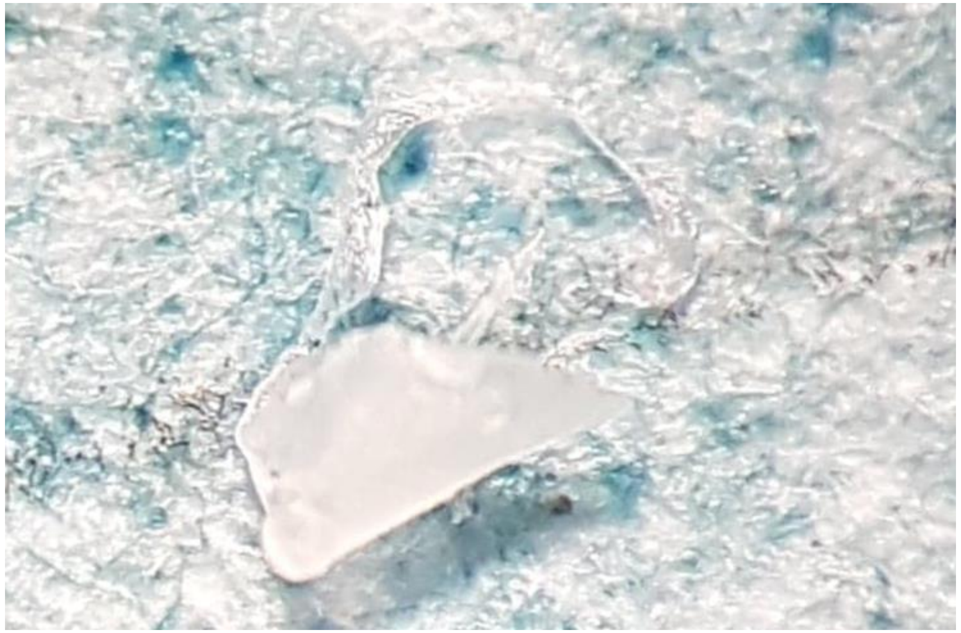

MetC5-2;1

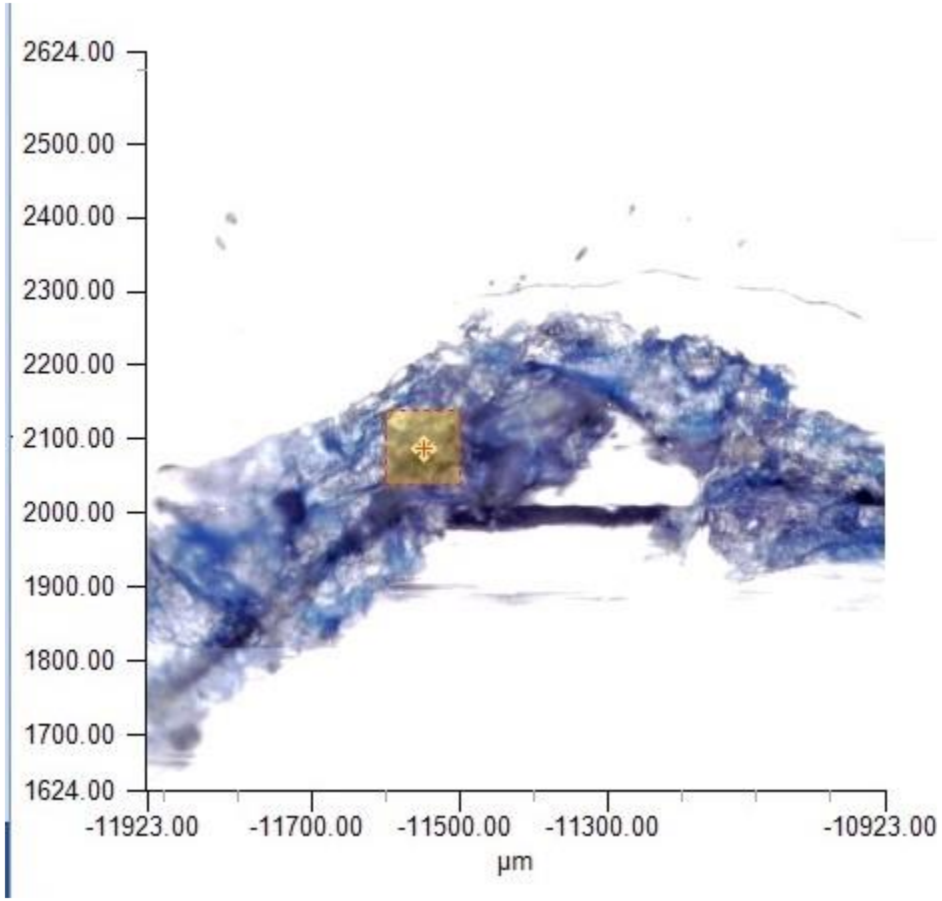
2624.00
2500.00
2400.00
2300.00
2200.00
2100.00
2000.00
1900.00
1800.00
1700.00
1624.00
-11923.00
-11700.00
-11500.00
-11300.00
-10923.00
µm

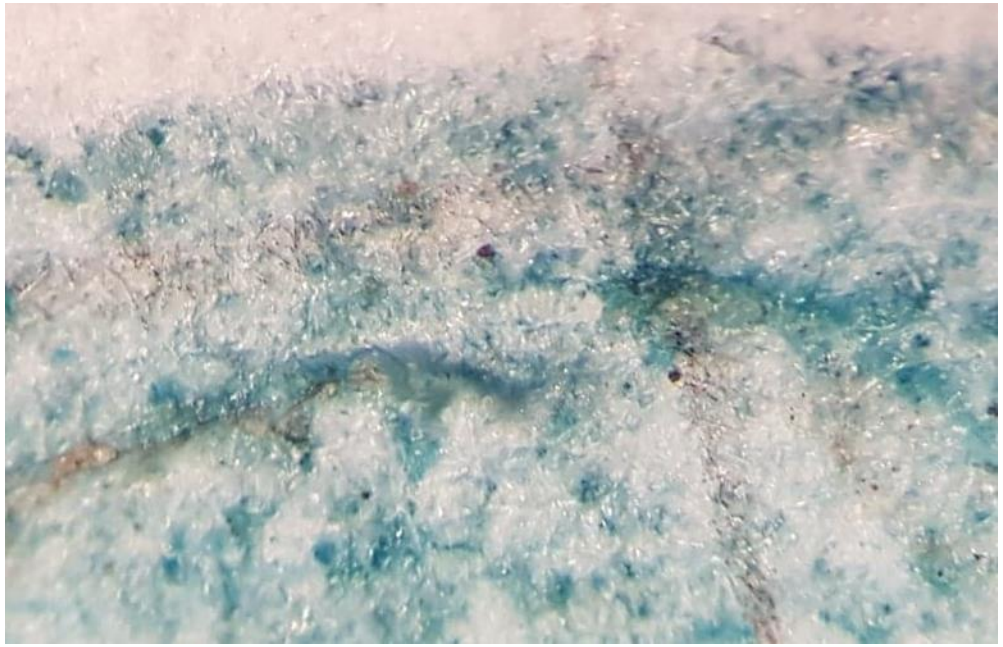

MetC5-3;1

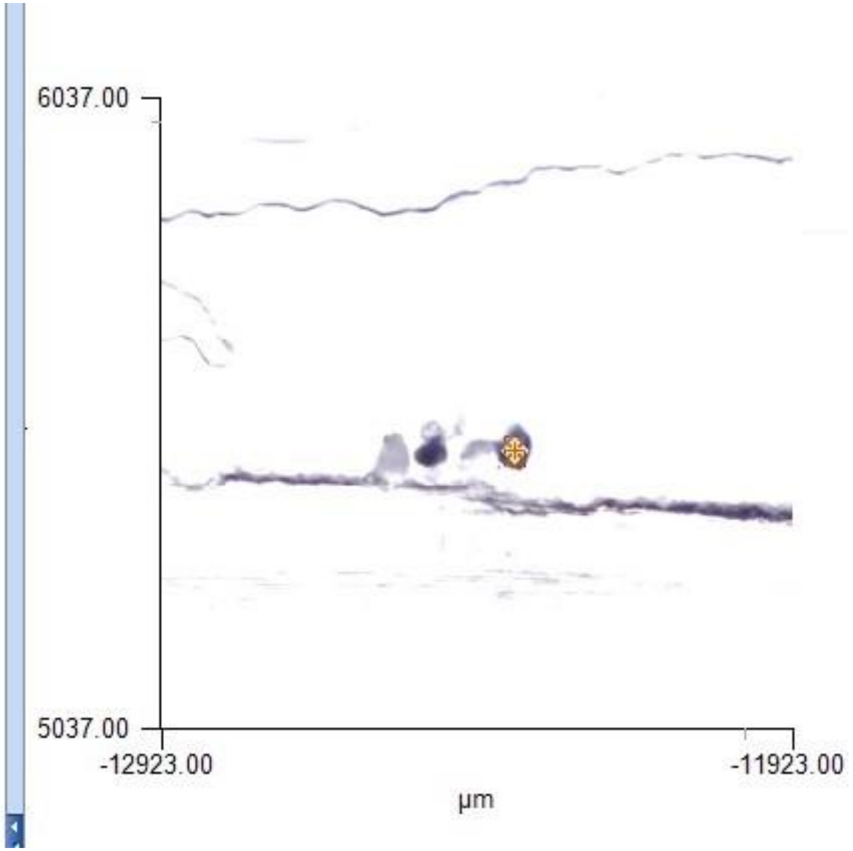

MetC5-4;1,2

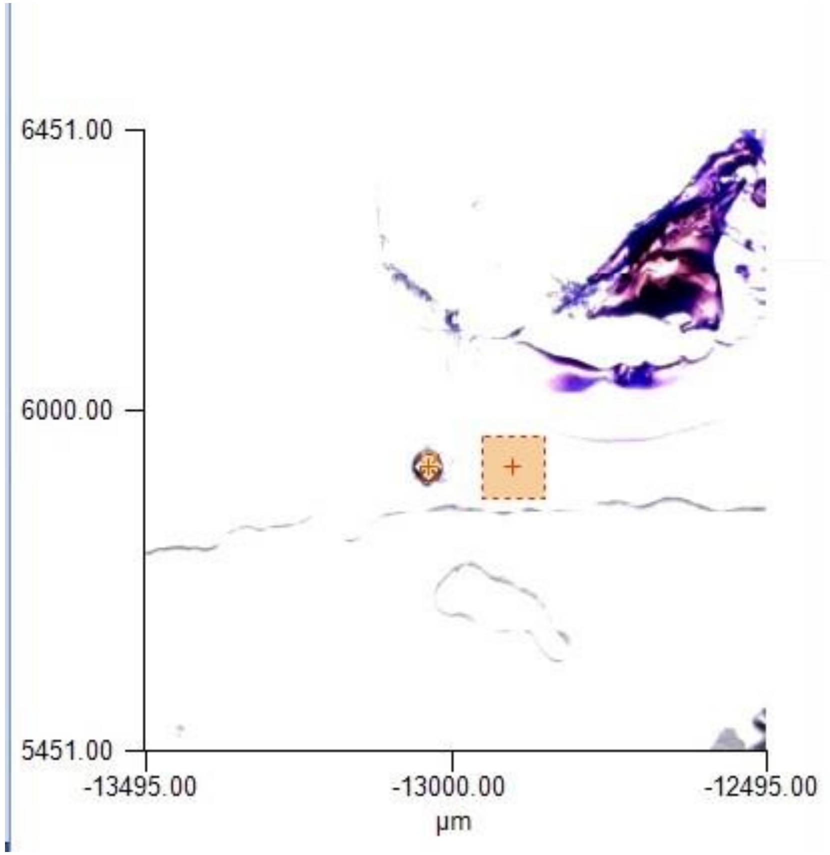

MetC6-1

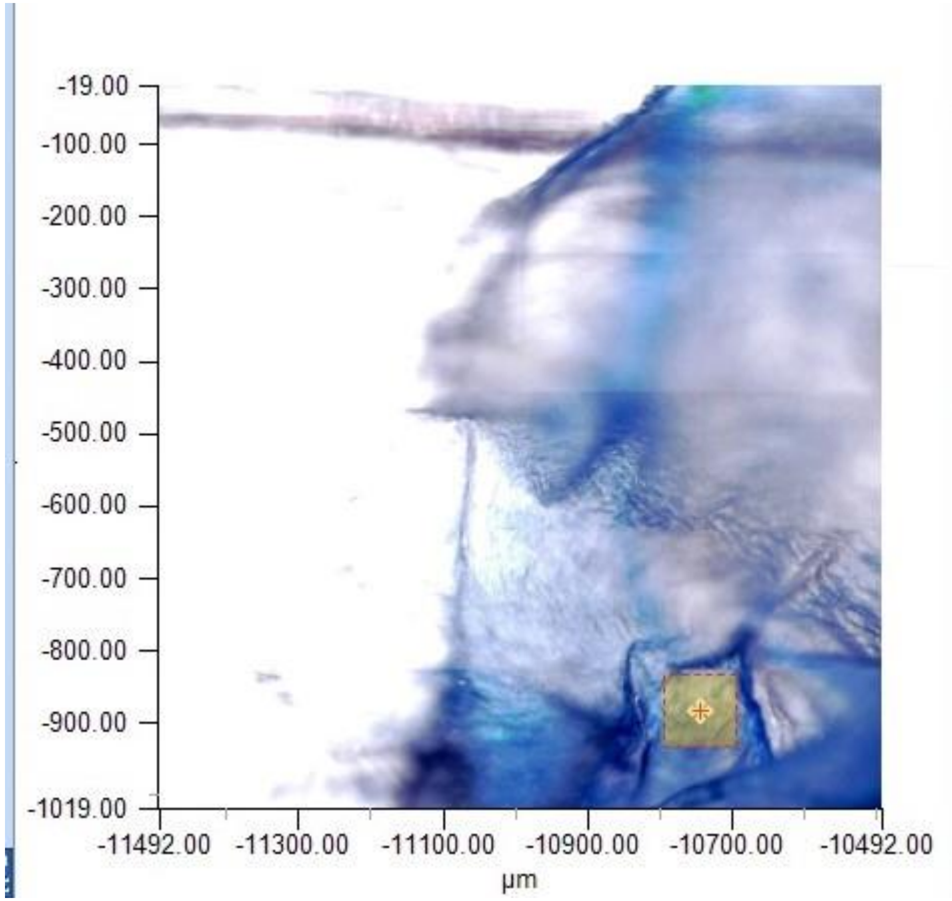
-19.00
-100.00
-200.00
-300.00
-400.00
-500.00
-600.00
-700.00
-800.00
-900.00
-1019.00
-11492.00
-11300.00
-11100.00
-10900.00
-10700.00
-10492.00
µm

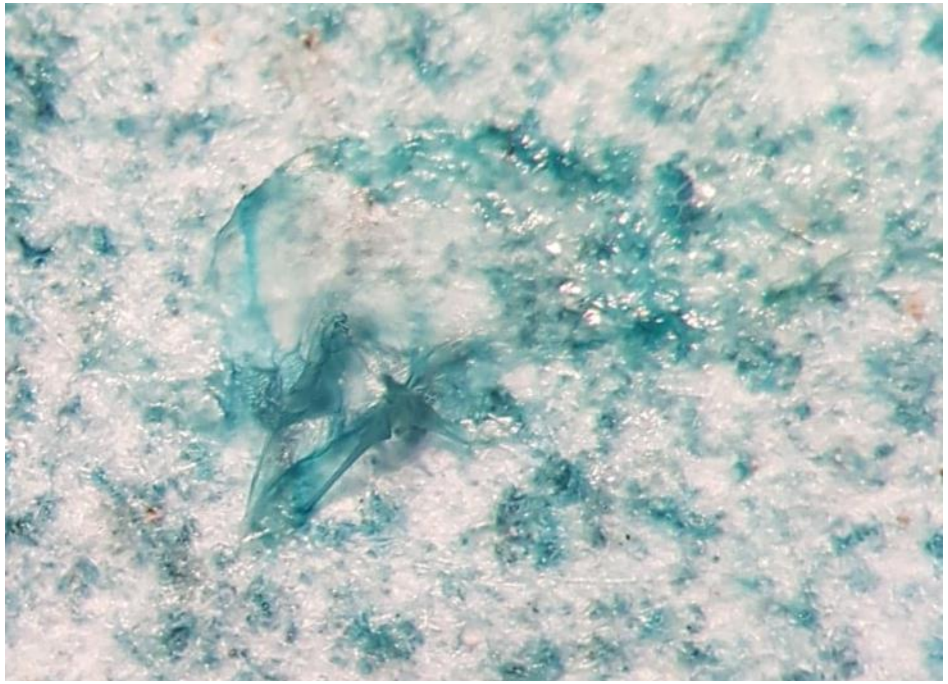

MetC6-2

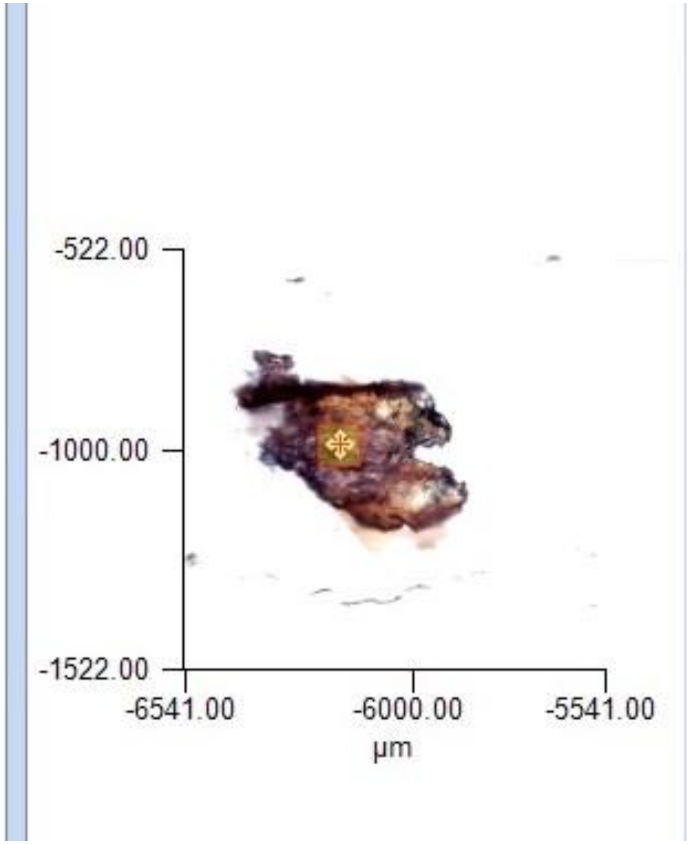

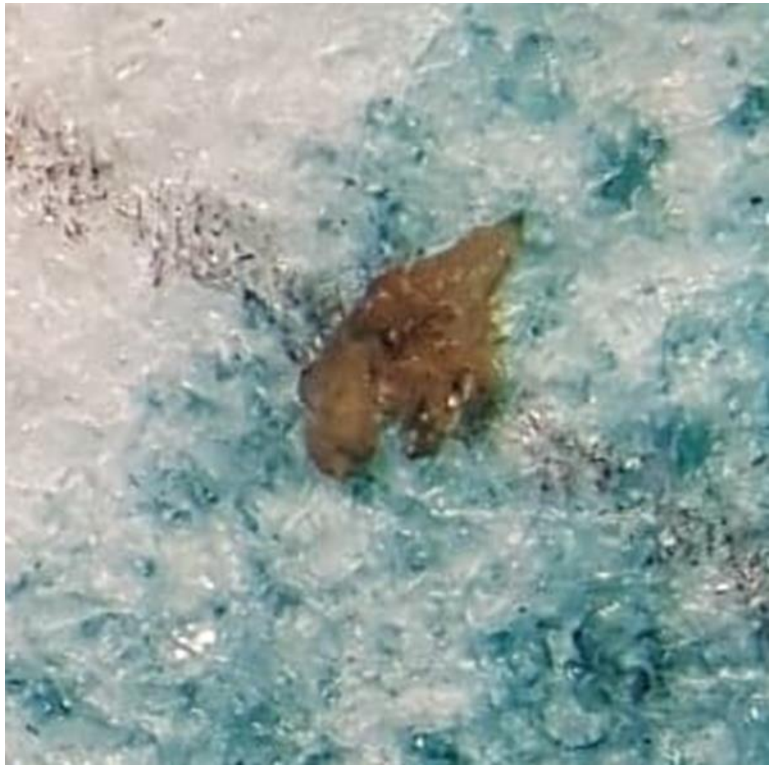

MetC6-3;1,2

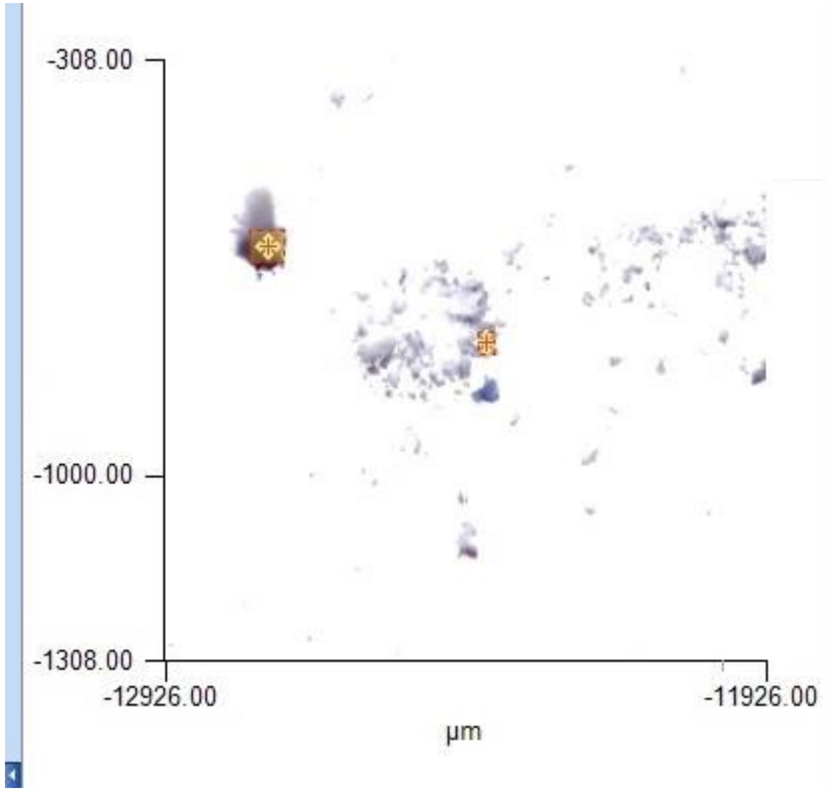

MetC6-4

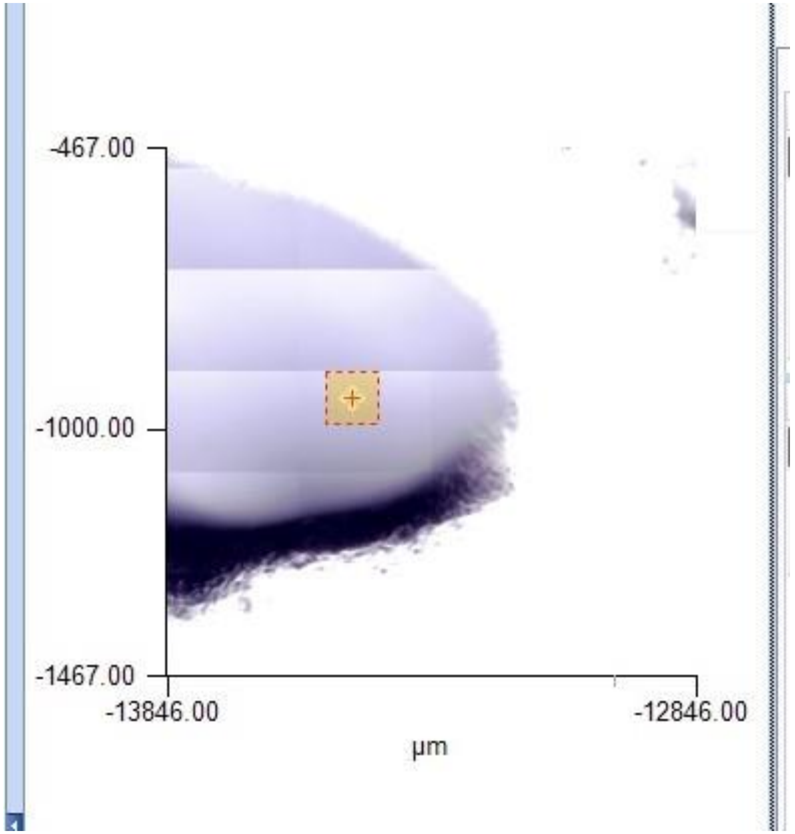

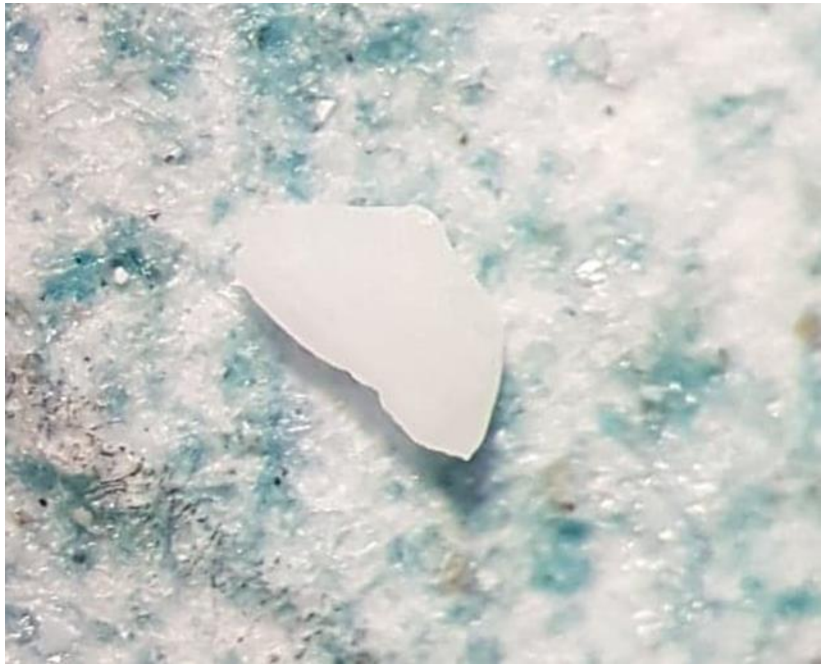

MetC7-1,1,2

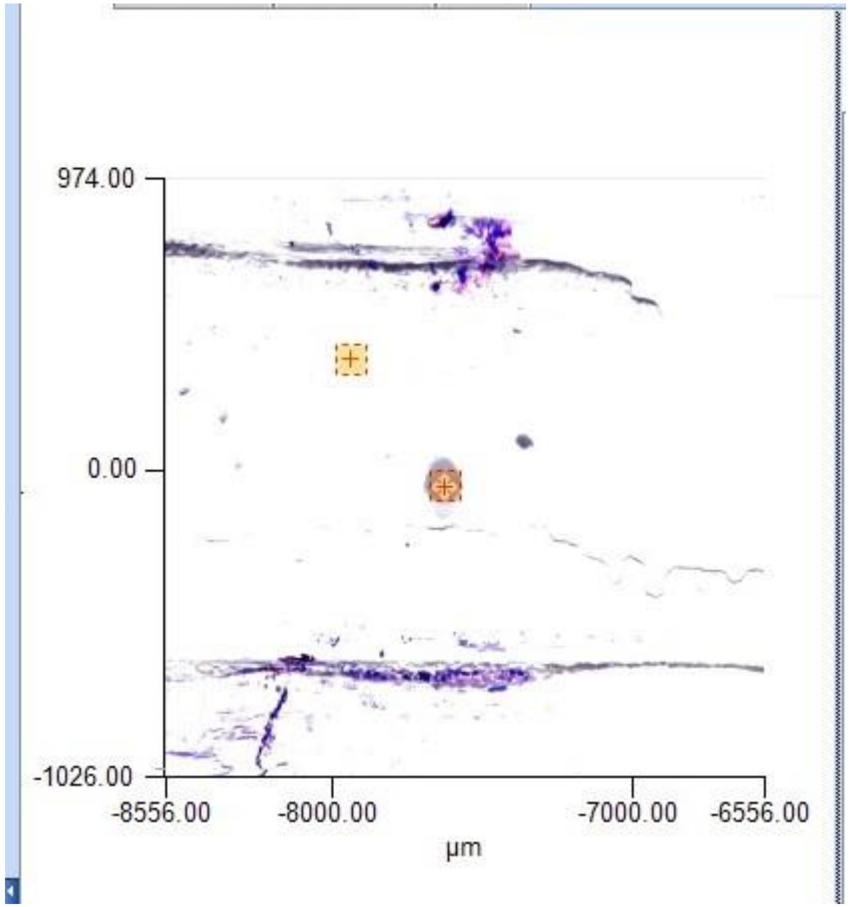

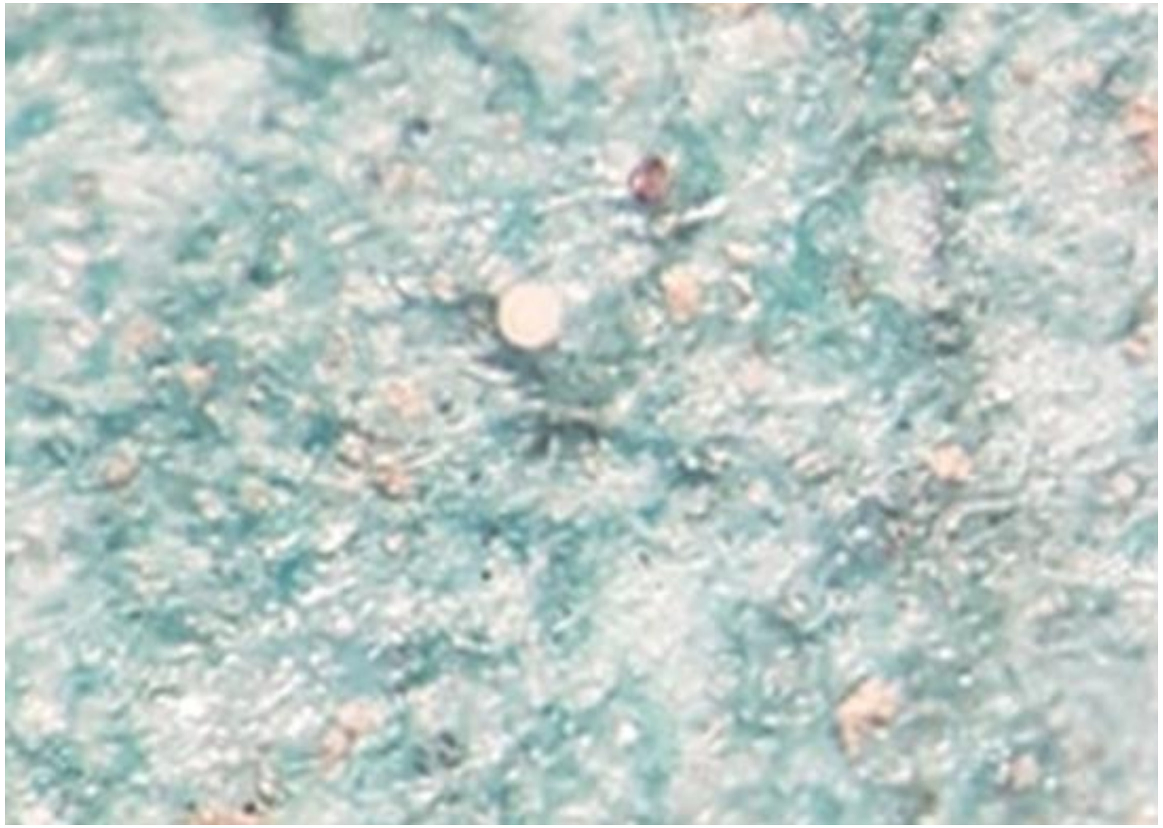

MetC7-2,1,2

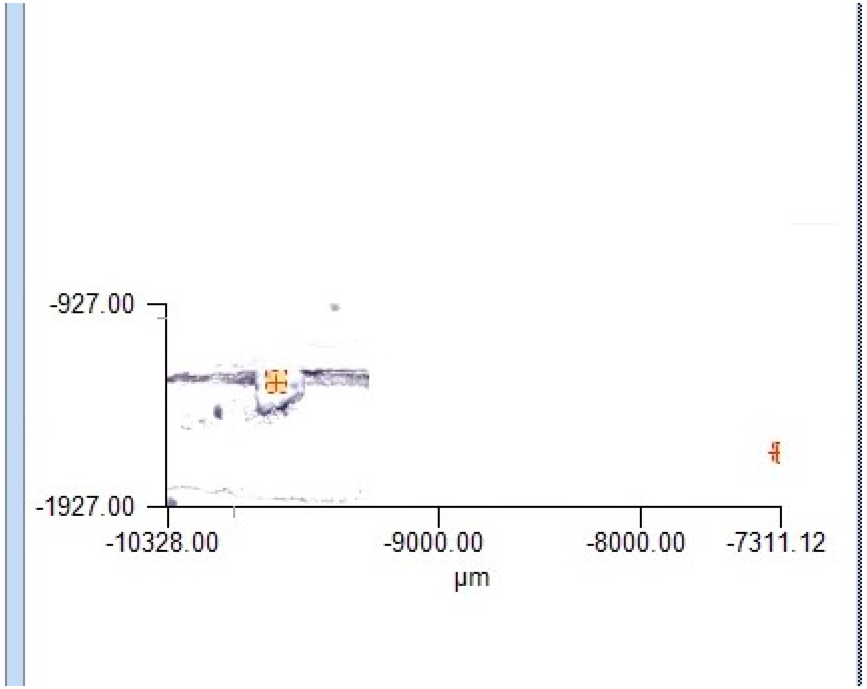

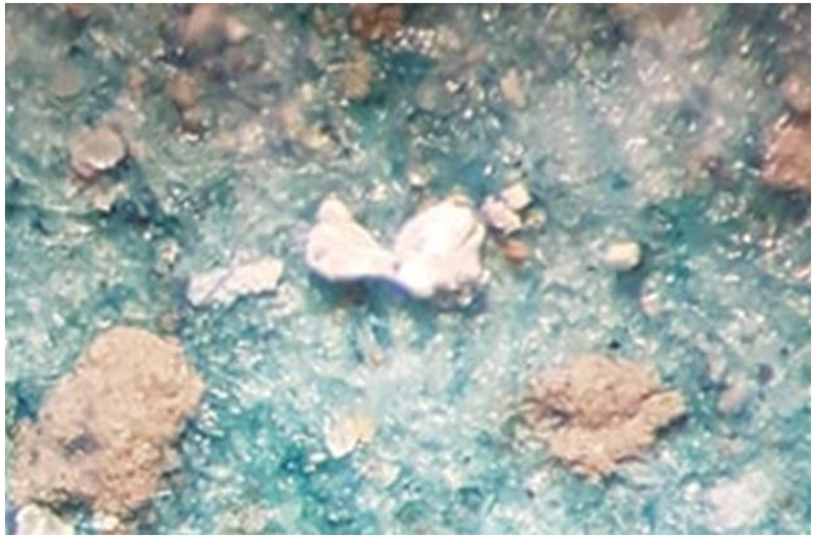

MetC7-3

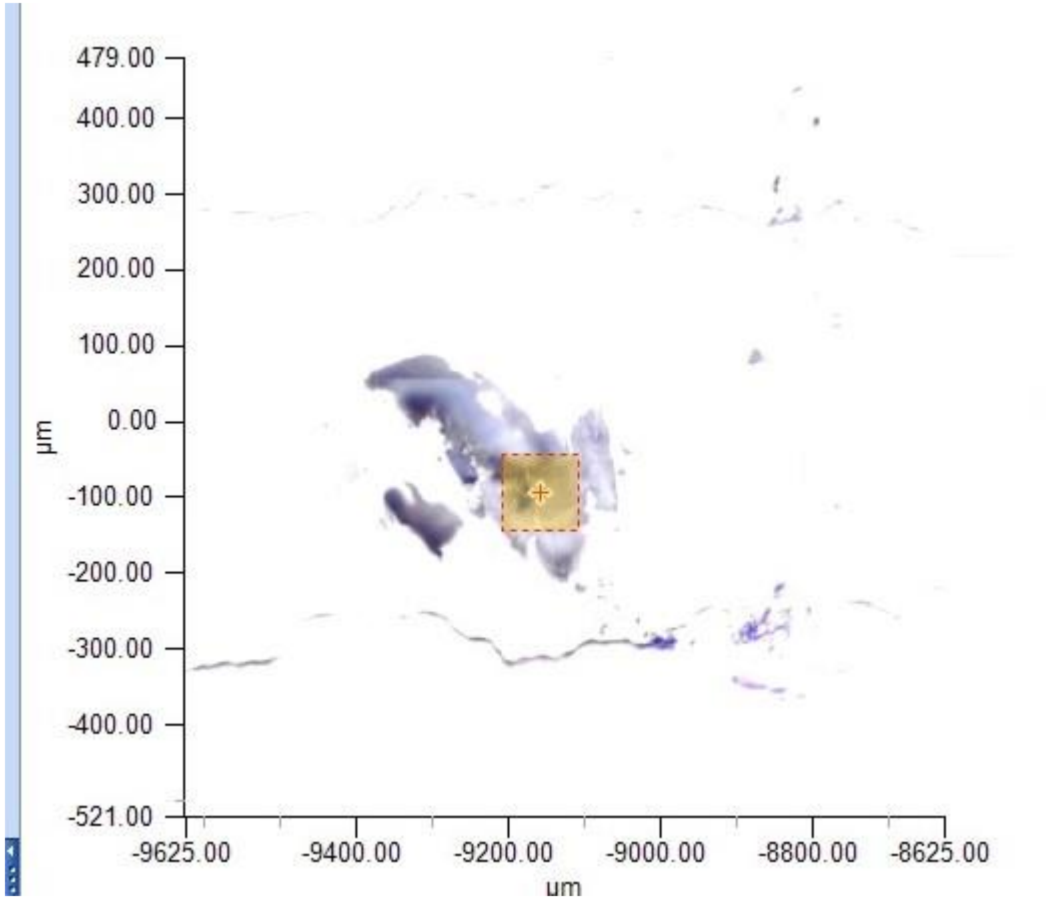

479.00
400.00
300.00
200.00
100.00
0.00
-100.00
-200.00
-300.00
-400.00
-521.00
µm
-9625.00
-9400.00
-9200.00
-9000.00
-8800.00
-8625.00
um

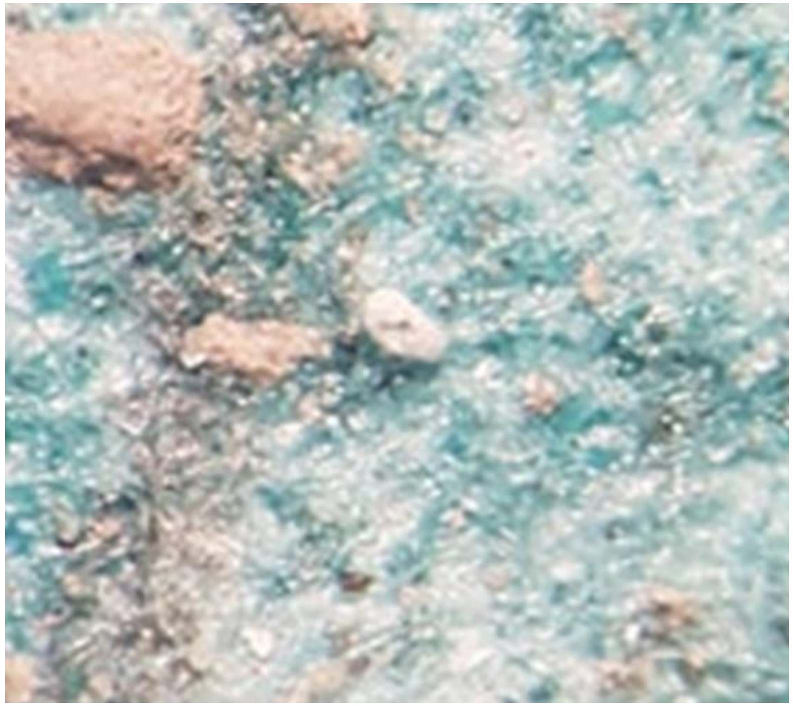

MetC7-4,1,2,3

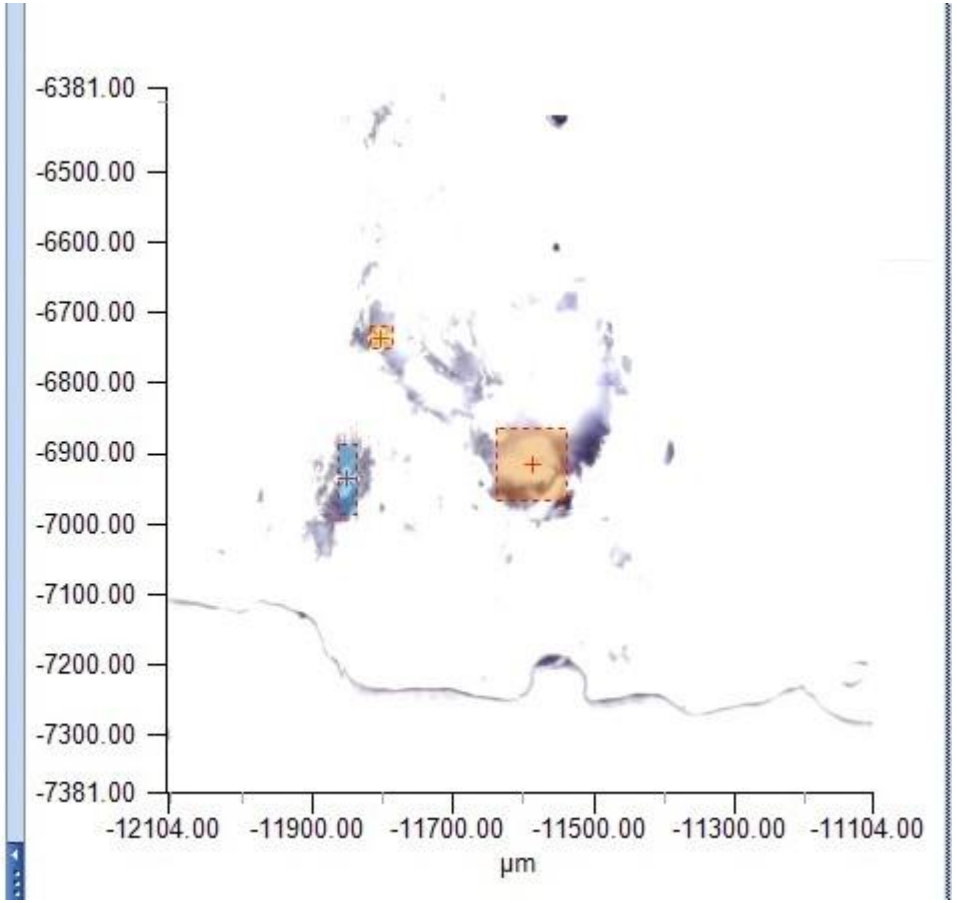

-6381.00
-6500.00
-6600.00
-6700.00
-6800.00
-6900.00
-7000.00
-7100.00
-7200.00
-7300.00
-7381.00
-12104.00
-11900.00
-11700.00
-11500.00
-11300.00
-11104.00
µm

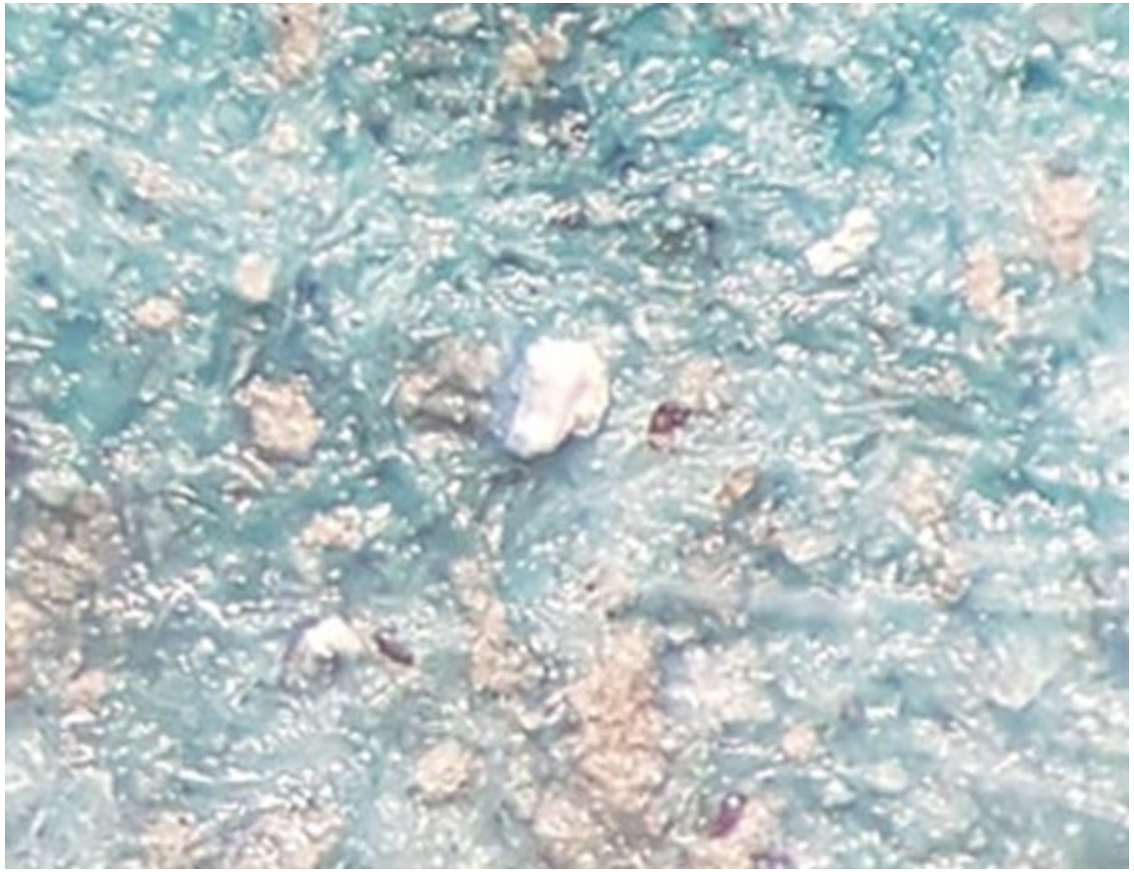

MetC9-1-1,2,3,4

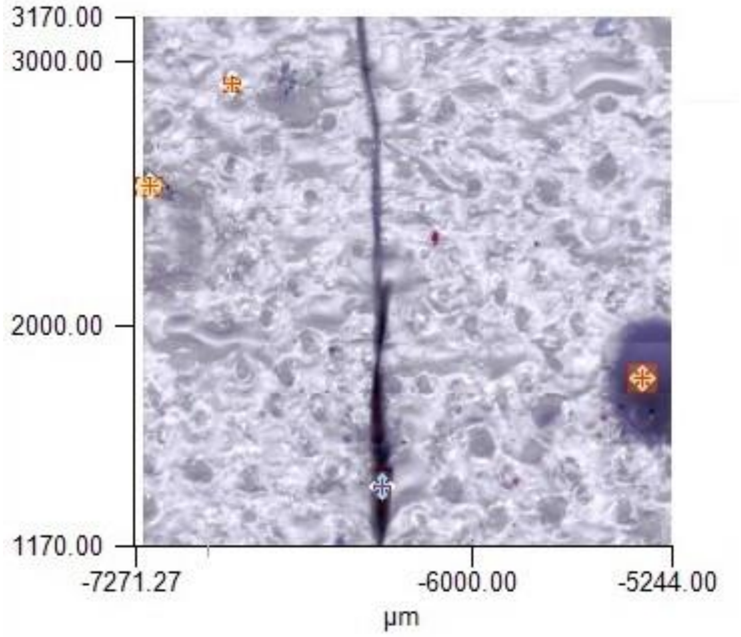

Metc9-2red

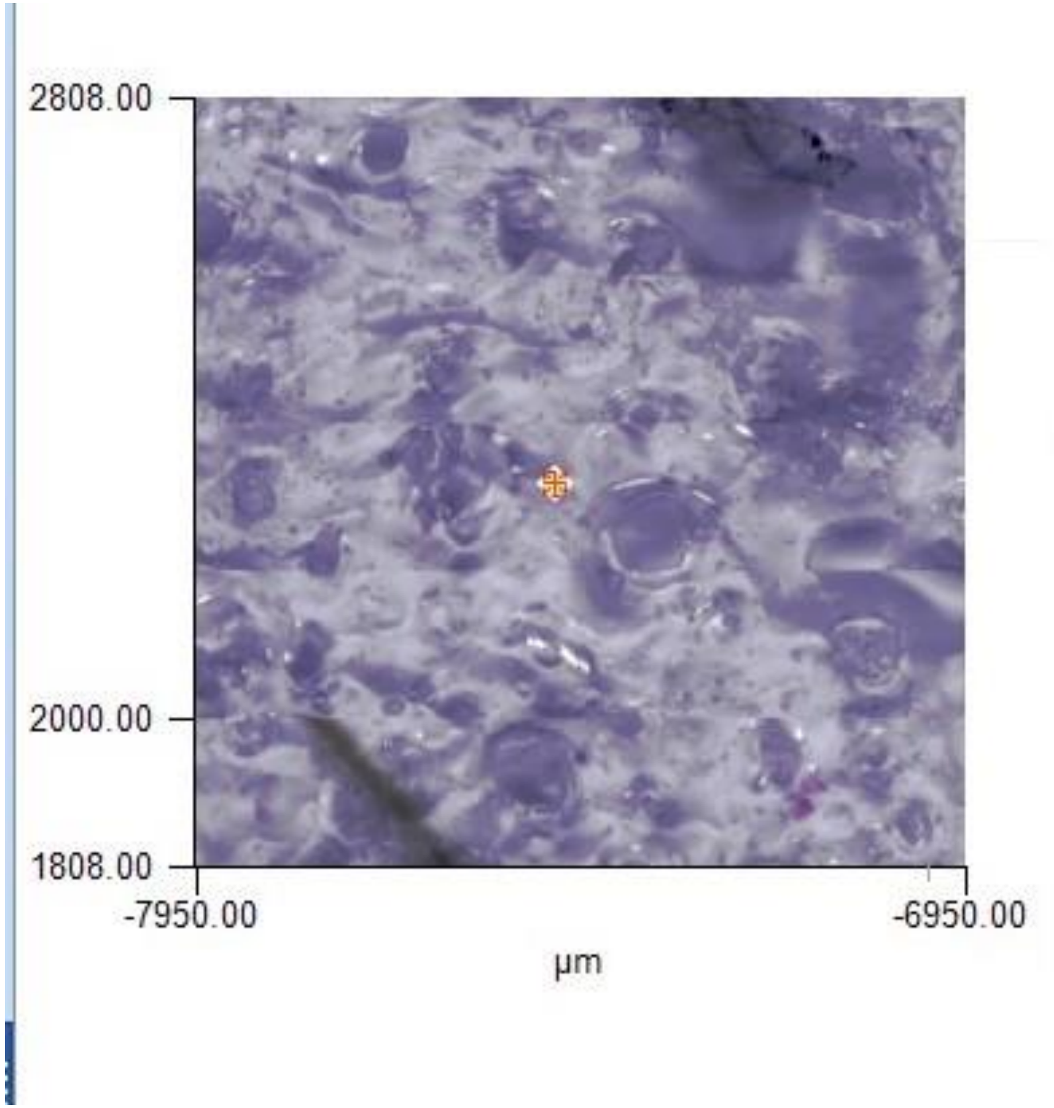

NHBC3-1,2

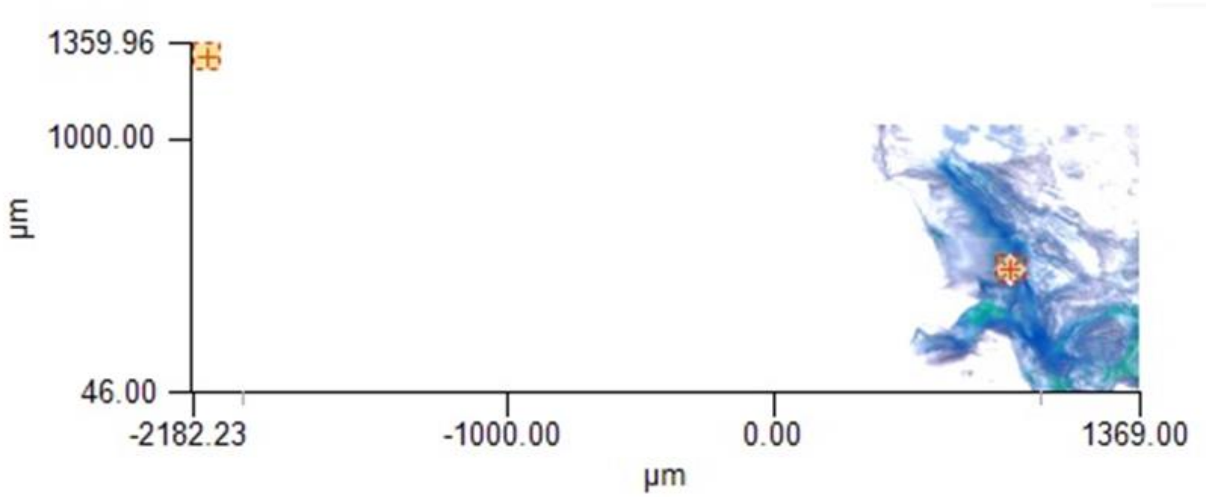

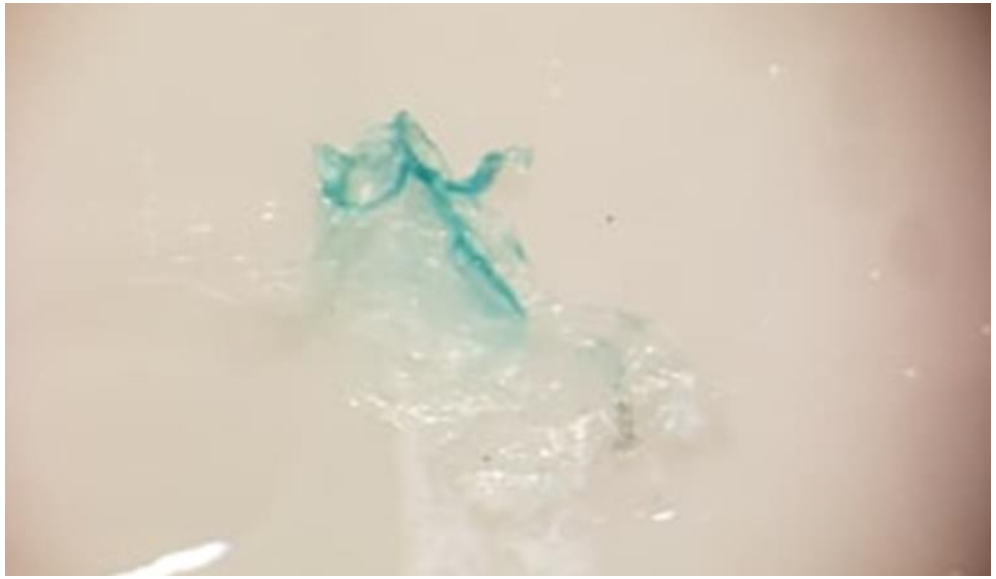

NHBC3-2

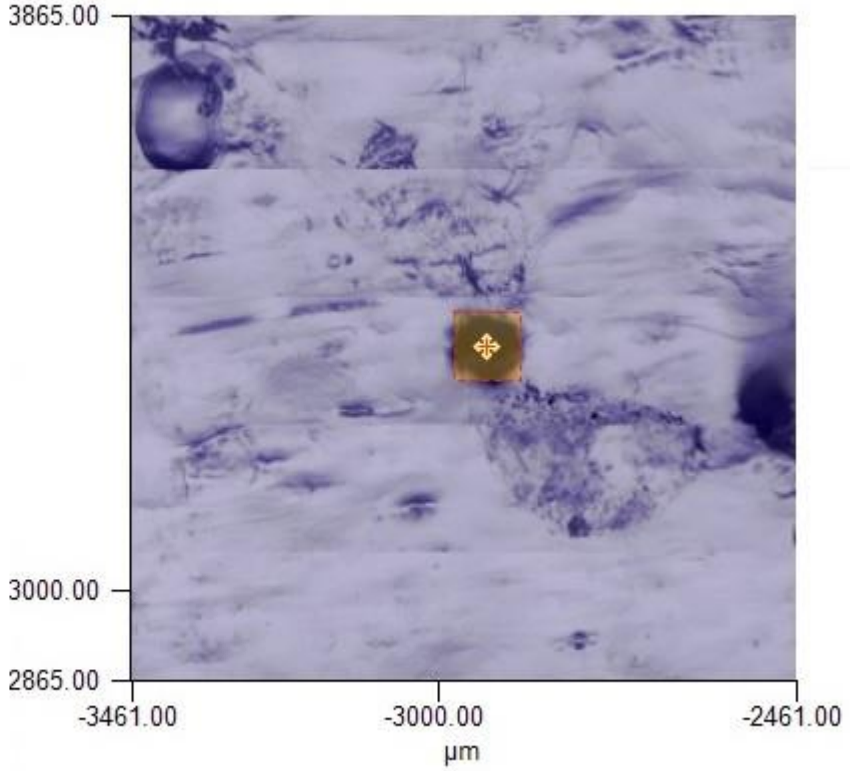

NHBC3-3,1,2

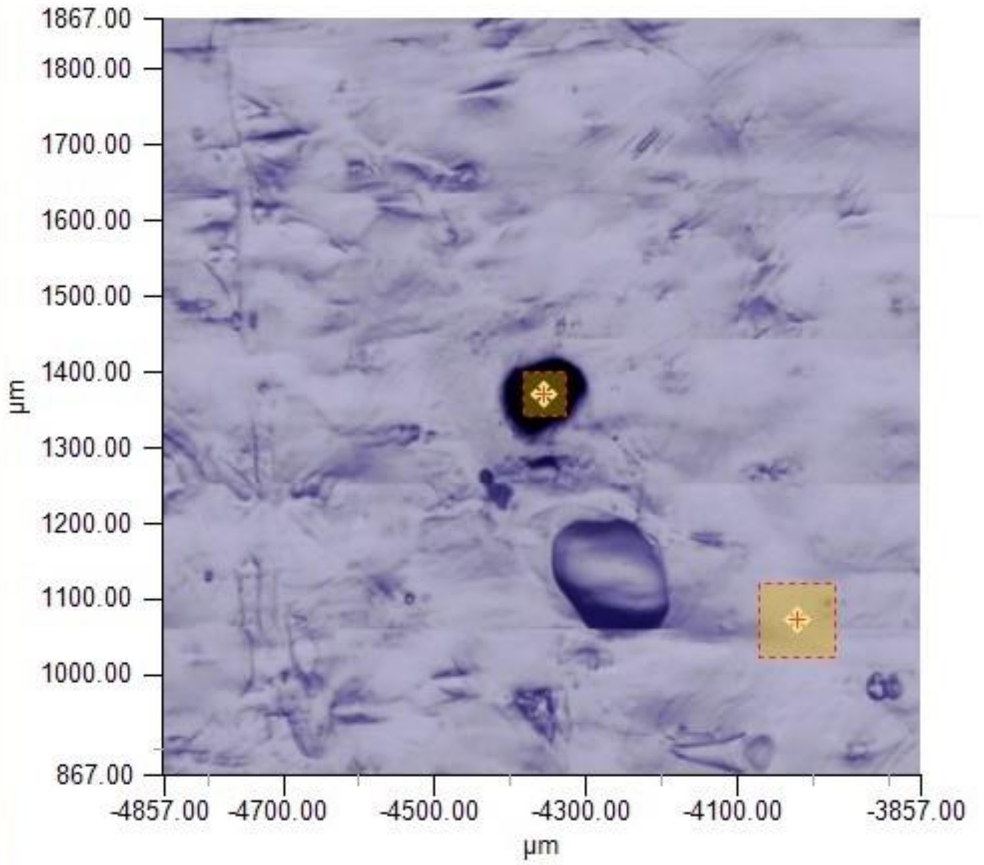

1867.00
1800.00
1700.00
1600.00
1500.00
1400.00
1300.00
1200.00
1100.00
1000.00
867.00
µm
-4857.00
-4700.00
-4500.00
-4300.00
-4100.00
-3857.00
µm

NHBC3-4

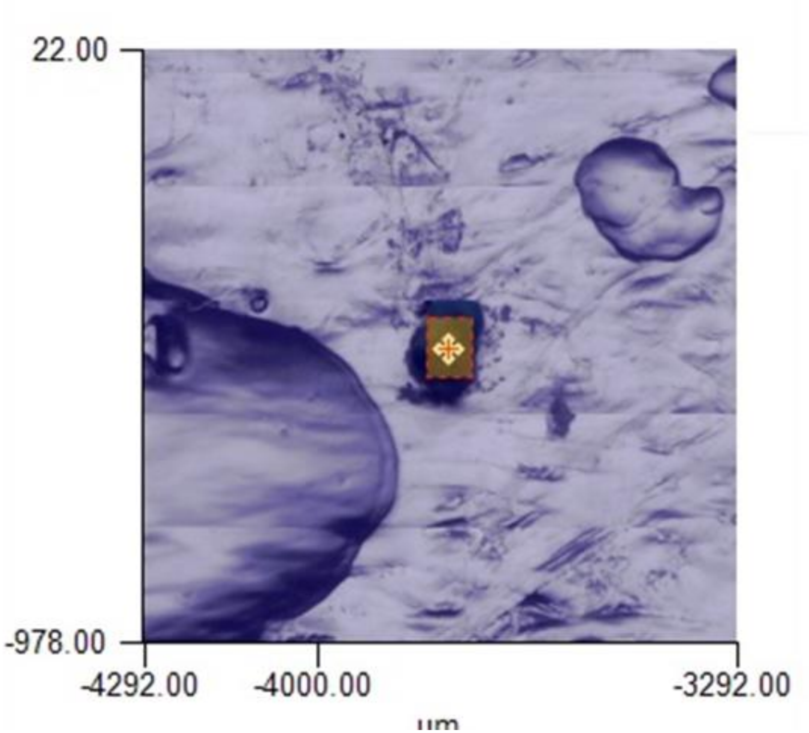

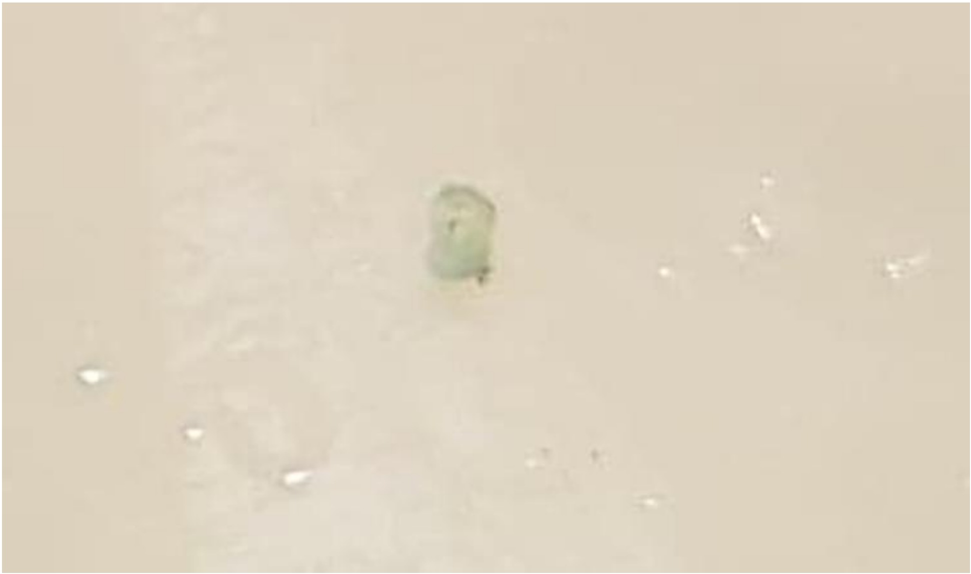

NHBC4-1

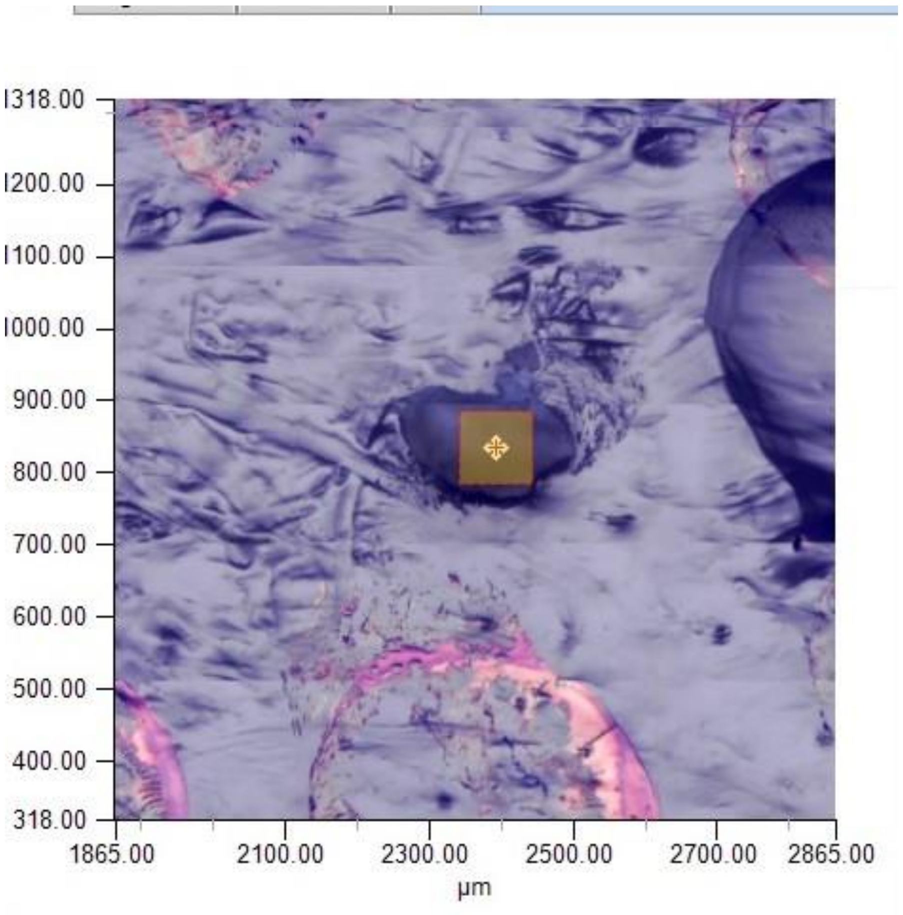

1318.00
1200.00
1100.00
1000.00
900.00
800.00
700.00
600.00
500.00
400.00
318.00
1865.00
2100.00
2300.00
2500.00
2700.00
2865.00
µm

NHBC4-2

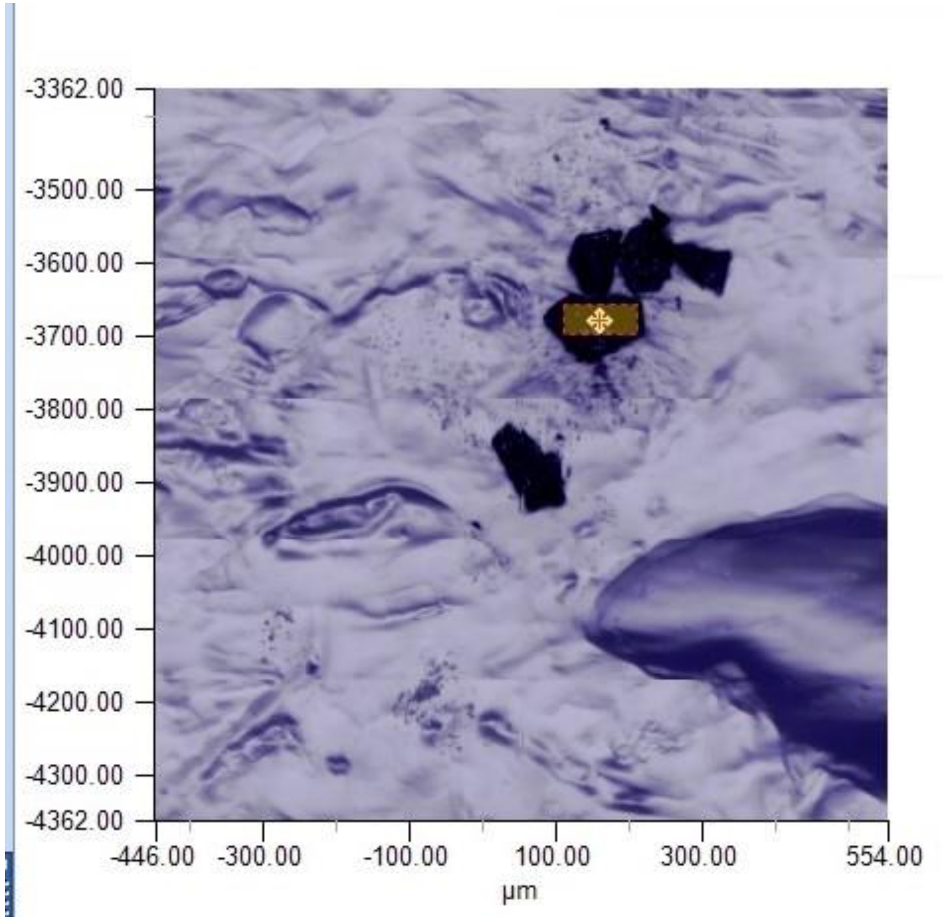

-3362.00
-3500.00
-3600.00
-3700.00
-3800.00
-3900.00
-4000.00
-4100.00
-4200.00
-4300.00
-4362.00
-446.00
-300.00
-100.00
100.00
300.00
554.00
µm

NHBC35-1, 2, 3

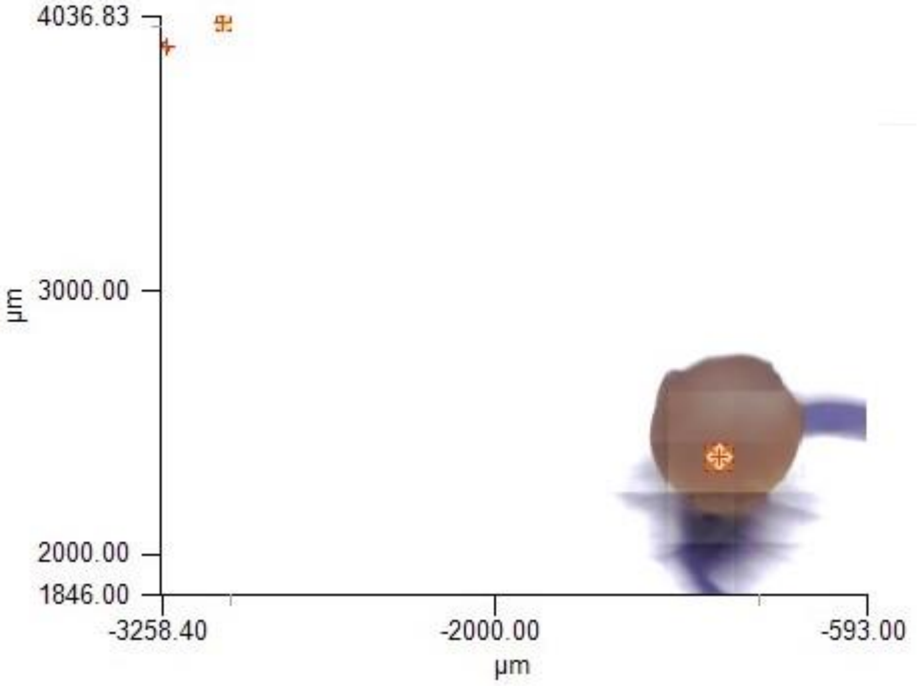

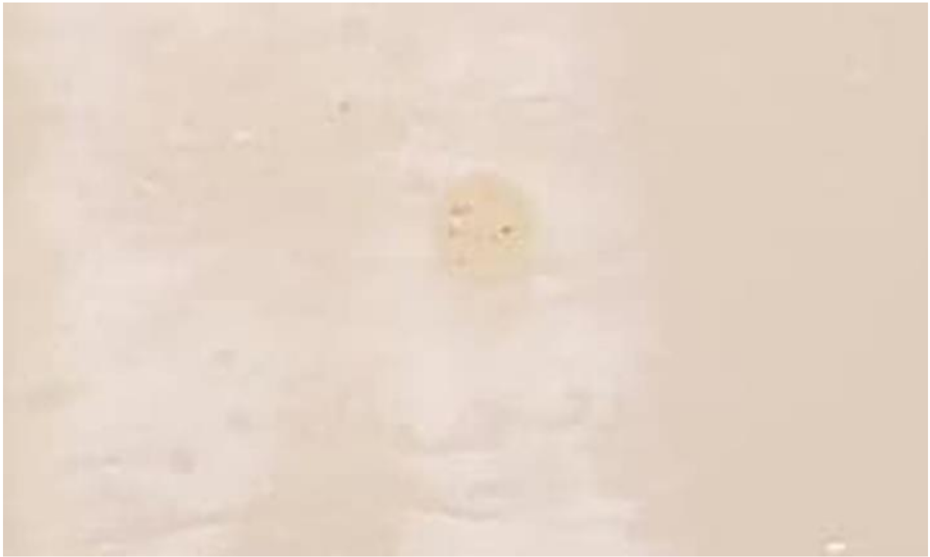

NHBC36-1,2

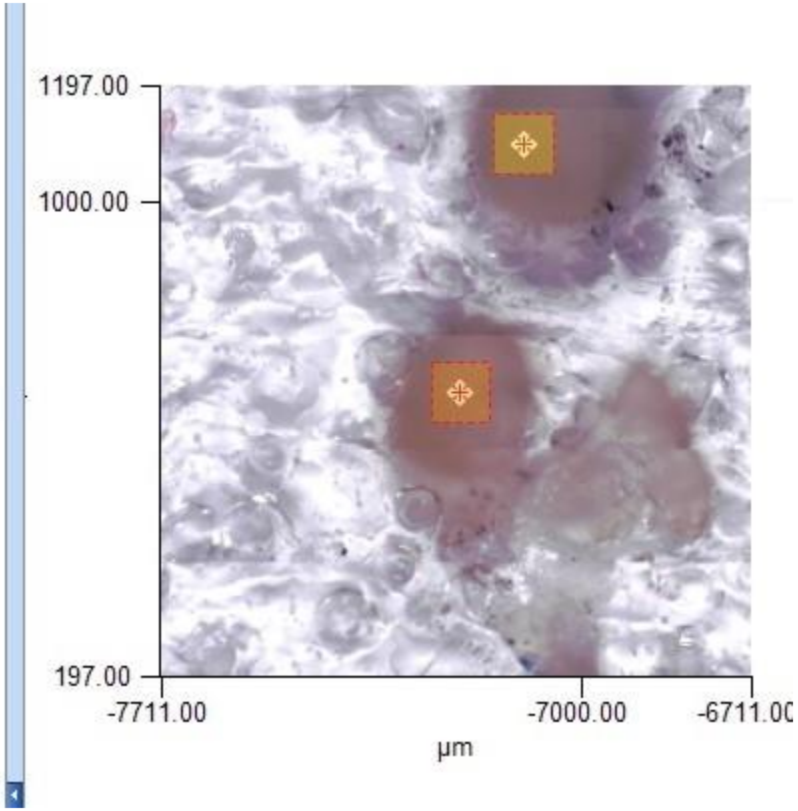

NHBC36-1

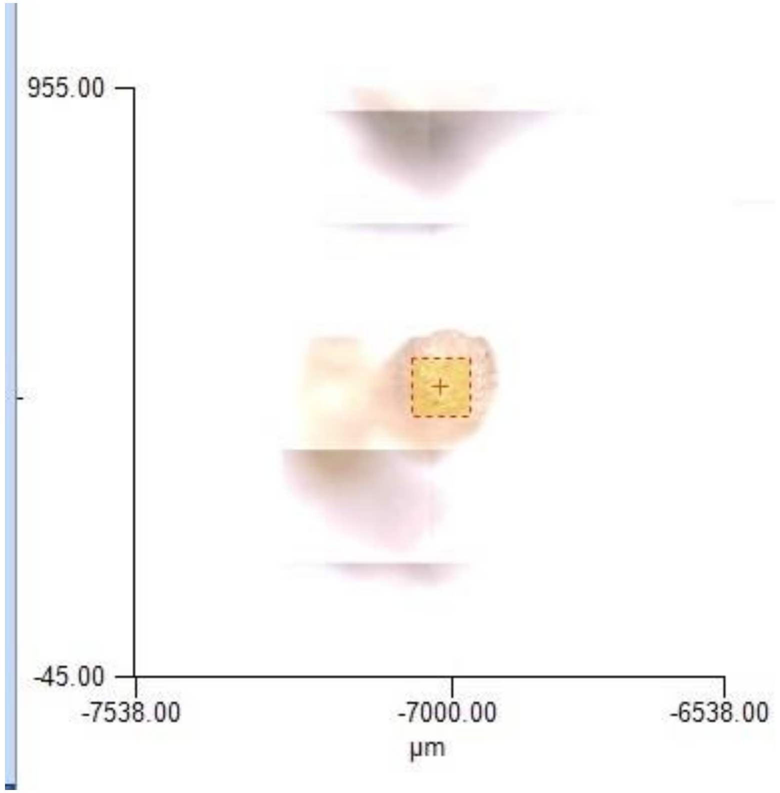

NHBC 40

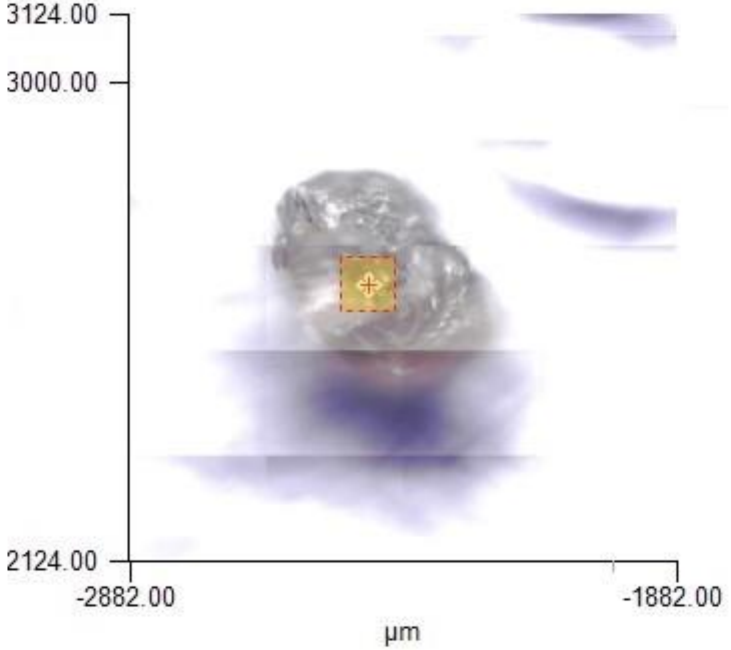

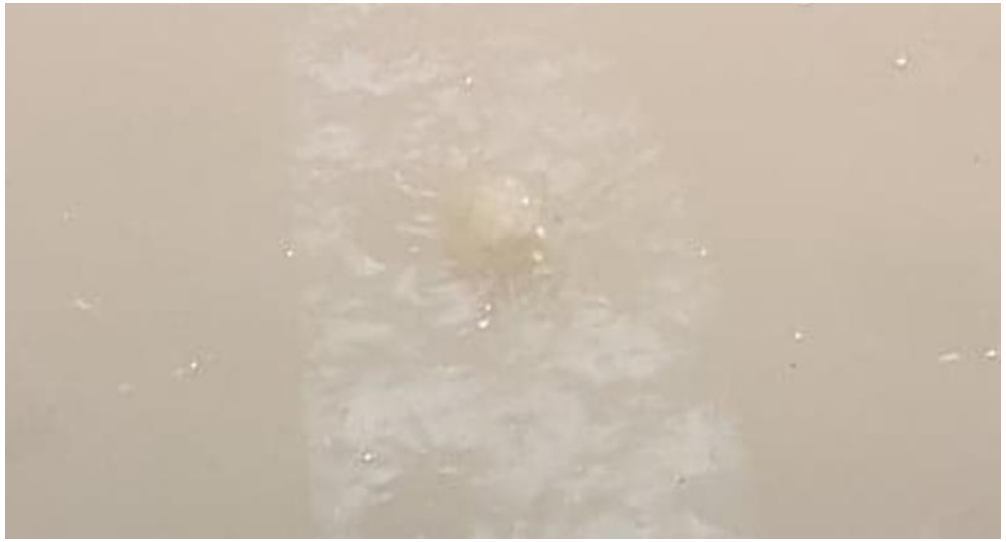

RUC51-1,2

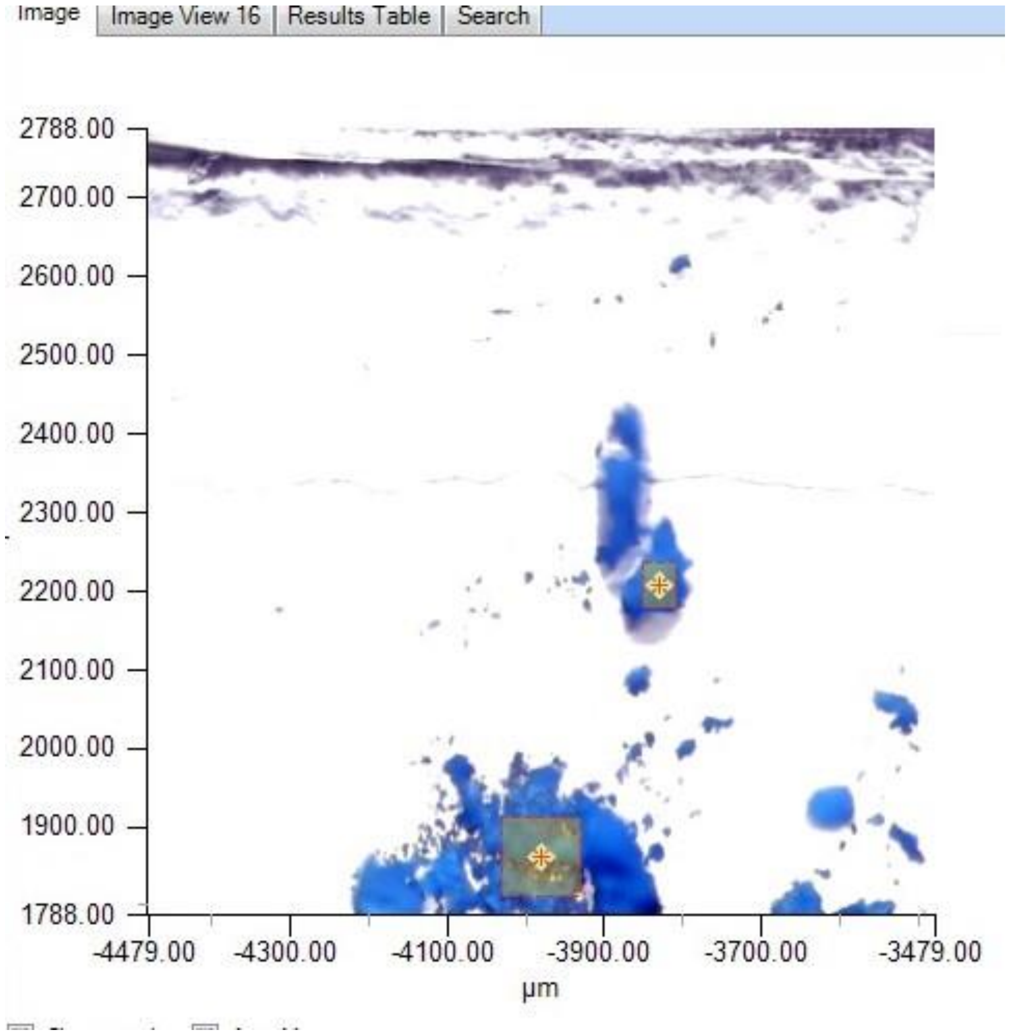
Image
Image View 16
Results Table
Search
2788.00
2700.00
2600.00
2500.00
2400.00
2300.00
2200.00
2100.00
2000.00
1900.00
1788.00
-4479.00
-4300.00
-4100.00
-3900.00
-3700.00
-3479.00
µm

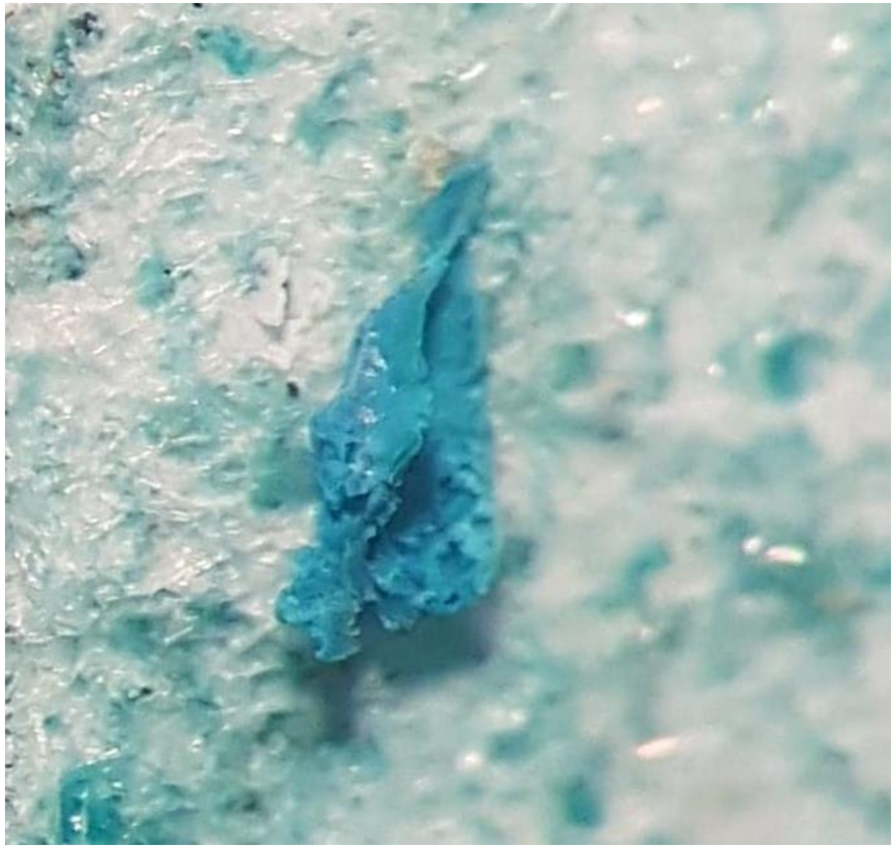

Supplement: S2 Fig — (PDF) [file pone.0232879.s002.pdf]
