# Supplementary material for: Use of sediment dwelling bivalves to biomonitor plastic particle pollution in intertidal regions; A review and study
Source: PLoS One. 2020 May 22;15(5):e0232879. doi: 10.1371/journal.pone.0232879 (PMC7244099; doi:10.1371/journal.pone.0232879)
Supplement: S1 Table — (PDF) [file pone.0232879.s003.pdf]

Table 6. Polymer ID for all particles recovered (supplementary material)

SITE	SAMPLE ID				SHAPE	POLYMER
**BMP**		1 bbis		1	film	HDPE
				2	film	HDPE
				3	frag	HDPE
				4	film	HDPE
**BMP**	G1			1	frag	sand
				2	frag	PS
**BMP**		1		1	frag	HDPE
				2	frag	HDPE
				3	frag	HDPE
				4	frag	HDPE
				5	frag	HDPE
**BMP**		1 bbissec		1	frag	HDPE
				2	frag	PU
**BMP**		5		1	frag	PPC
				2	frag	PPC
**BMP**	G2			1	frag	PS
				2	frag	PS
**BMP**	G3			1	frag	PPC
				2	frag	PPC
				3	frag	PPC
**BMP**		4		1	frag	PPC
**BMP**	G5			1	frag	PPC
				2	frag	PPC
**BMP**		7		1	frag	Nylon
				2	frag	Nylon
**BMP**		8		1	frag	Nylon
				2	frag	Nylon
**BMPALL**				1	frag	PPC
				2	frag	PPC
				3	frag	PPC
				4	frag	PPC
				5	frag	PPC
				6	fiber	HDPE
**CP**		5		1	frag	HDPE
				2	film	PPC
				3	frag	sand
**EB**		1			frag	sand
**EB**		2	1		frag	PPC
			2		fiber	HDPE
**EB**		3			fiber	PPC
**EB**		4			fiber	PPC
**EB**		6	1		frag	sand
**EB**		7	1		bead	unk
**EB**		7	2		frag	unk
**EB**		7	3		frag	HDPE

**EB**	7	4		frag	HDPE
**EB**	7	5	1	frag	HDPE
			2	frag	unk
			3	frag	HDPE
**EB**	7	6		frag	unk
**EB**	8	1	1	frag	HDPE
			2	?	unk
**EB**	8	2	1	frag	HDPE
			2	frag	unk
			3	frag	CYANOX
			4	frag	HDPE
**EB**	8	3	1	frag	HDPE
			2	frag	unk
**EB**	9	2	1	frag	unk
			2	frag	HDPE
			3	frag	PS
**J**		1	1	frag	HDPE
			2	frag	HDPE
			3	frag	HDPE
			4	frag	NS
			5	frag	HDPE
			6	frag	HDPE
**J**			21	frag	CYANOX
			22	frag	CYANOX
**J**	1a		1	frag	CYANOX
**J**		1	31	frag	HDPE
			32	fibre	HDPE
**J**		1	1	frag	CaCO3
			2	frag	CaCO3
**J**		3	1	frag	sand
			2	frag	sand
			3	frag	sand
			4	bead	HDPE
			5	bead	HDPE
**J**		4	1	frag	HDPE
			2	frag	UNK
**J**	7	2	1	frag	PPC
			2	frag	PPC
			3	frag	PPC
			4	frag	PPC
			5	fibre	PPC
			6	fibre	PU
**Met**	1		1	frag	PPC
			2	bead	sand
**Met**	1	2	1	frag	PPC

**Met**	4		1	fibre	HDPE
**Met**	4		2	fibre	HDPE
**Met**	4	3	1	frag	HDPE
			2	frag	HDPE
**Met**	4	1	1	bead	HDPE
			2	frag	HDPE
			3	frag	PPC
			4	frag	PPC
			5	frag	PPC
**Met**	5	1	1	frag	HDPE
			2	fiber	HDPE
**Met**	5	2	1	fiber	HDPE
**Met**	5	3	1	frag	PPC
**Met**	5	4	1	bead	HDPE
			2	frag	HDPE
**Met**	6		1	frag	HDPE
**Met**	6		2	frag	sand
**Met**	6	3	1	frag	HDPE
			2	frag	PU
**Met**	6		4	frag	CaCO3
**Met**	7	1	1	bead	unk
			2	bead	unk
**Met**	7	2	1	frag	PU
			2	frag	HDPE
**Met**	7	3	1	frag	unk
**Met**	7	4	1	bead	HDPE
			2	frag	PMMA
			3	frag	PMMA
**Met**	9	1	1	frag	HDPE
			2	frag	HDPE
			3	frag	PPC
			4	fibre	PPC
**Met**	9	2	1	film	unk
**NHB**	3		1	frag	HDPE
			2	Film	PPC
**NHB**	3		2	bead	HDPE
**NHB**	3	3	1	bead	HDPE
			2	frag	HDPE
**NHB3**	4		1	bead	HDPE
**NHB4**			1	frag	HDPE
**NHB4**			2	frag	HDPE
**NHB**	35		1	frag	NS
			2	frag	NS
			3	bead	PMMA
**NHB**	36		1	bead	PMMA
			2	bead	PMMA

**NHB**	36		1	bead	HDPE
**NHB**	40		1	bead	HDPE
**RU**	5	1	1	frag	HDPE
			2	frag	HDPE
